# Supplementary material for: Gnb5 is a negative regulator of the BACE1-mediated Aβ generation and ameliorates cognitive deficits in a mouse model of Alzheimer’s disease
Source: PLoS Biol. 2025 Jun 30;23(6):e3003259. doi: 10.1371/journal.pbio.3003259 (PMC12233908; doi:10.1371/journal.pbio.3003259)
Supplement: S1 Text — (DOCX) [file pbio.3003259.s001.docx]

**Supplementary Information for**

**Gnb5 is a negative regulator of the BACE1-mediated Aβ generation and ameliorates cognitive deficits in a mouse model of Alzheimer's disease**

**Detailed Supplemental Methods and Materials**

**Western blotting**

Cultured cells and mouse brain tissues were homogenized in ice-cold RIPA lysis buffer for 40 min, followed by centrifugation at 12,000 × g for 10 min at 4°C. The supernatant was collected and protein concentration determined using BCA assay. Samples containing 30 μg protein were resolved on 10% SDS-PAGE gels and electrophoretically transferred to PVDF membranes using ice-cold transfer buffer (25 mM Tris-HCl, 192 mM glycine, 20% methanol) at 250mA for 1.5 h. Membranes were blocked with 5% non-fat milk in TBST for 2 h at room temperature, then incubated with primary antibodies diluted in blocking buffer at 4°C overnight. After three 10-min TBST washes, membranes were incubated with horseradish peroxidase-linked secondary antibodies (1:5,000) for 1 h at room temperature. Protein bands were visualized using Bio-Rad ECL reagents (Cat# 1705061) and quantified with ImageJ software by measuring integrated gray density values. Primary antibodies used for Western blotting include: Gnb5 (Rabbit ,1:2,000, Abcam, ab185206), App (Rabbit, 1:3,000, BBI LIFE, D260097-0025), BACE1 (Rabbit, 1:2,000, Cell Signaling Technology, 5606s), Nicastrin (Rabbit, 1:1,000, Cell Signaling Technology, 5665s), β-CTF (Rabbit, 1:2,000, Abcam, ab32136), α-Tubulin (Rabbit, 1:5,000, Protein-tech, 11224-1-AP), HA (Mouse, 1:1,000, Invitrogen, 26183), Myc (Mouse, 1:2,000, Cell Signaling Technology, 2276), GFP (Rabbit, 1:2,000, Cell Signaling Technology, 2956s), Tuj1 (Mouse, 1:2,000, BIOLEGEND, 801201), Gfap (Rabbit, 1:5,000, Cell Signaling Technology, 80788S), Iba1 (Rabbit, 1:2,000, Cell Signaling Technology, 17198S), Rgs7 (Rabbit, 1:500, ABclonal, A6720), Akt (Rabbit, 1:1,000, Cell Signaling Technology, 9272), p-Akt (Rabbit, 1:2,000, Cell Signaling Technology, 4060).

**Immunofluorescence**

Mice were transcardially perfused with 60 mL ice-cold phosphate buffered saline (PBS). Brains were dissected and post-fixed in 4% paraformaldehyde (PFA, w/v) for 24 h at 4°C, followed by sequential dehydration in sucrose gradients (20%, 25%, 30% in PBS) with 24 h incubation at each concentration. Coronal brain sections (15 μm thickness) were prepared using a cryostat and systematically mounted onto pre-treated glass slides for standardized histological procedures. After antigen retrieval with citrate buffer (pH 6.0), sections were blocked with 5% BSA/0.3% Triton X-100 in PBS for 1 h at room temperature. Primary antibodies diluted in blocking buffer were applied overnight at 4°C. After three 10-min PBS washes, sections were incubated with species-matched Alexa Fluor-conjugated secondary antibodies (1:500; Invitrogen) for 2 h at room temperature. Nuclei were counterstained with DAPI (1 μg/mL, Sigma-Aldrich) according to standard protocols. Imaging was performed using an Olympus FV1000MPE upright two-photon microscope. Primary antibodies used for immunostaining include: Gnb5 (Rabbit, 1:1,000, Abcam, ab185206), Aβ (Mouse, 1:1,000, Abcam, ab126649), NeuN (Mouse, 1:1,000, Abcam, ab104224), Gfap (Mouse, 1:500, Cell Signaling Technology, 3670S) and Iba1 (Guinea pig, 1:200, SYSY, 234308).

**Quantitative RT-PCR**

Total RNA was isolated from mouse tissues and cultured cells using TRIzol reagent (Invitrogen) following the manufacturer's protocol. RNA concentration and purity were determined by spectrophotometry (A260/A280 ratio > 1.8). Reverse transcription was performed with 2 μg total RNA using ReverTra Ace qPCR RT Master Mix (Toyobo, FSQ-201) in a 20 μL reaction volume. Quantitative PCR amplification was carried out using LightCycler 96 SYBR Green Master Mix (Roche, 4913914001). All reactions were performed in triplicate with no-template controls. GAPDH mRNA levels were quantified as the endogenous reference for normalization. Primer sequences are provided in Table A.

**Primary neurons, astrocytes, microglia culture procedures**

Primary neurons were isolated from embryonic day 16.5 (E16.5) pregnant mice. Briefly, cortical tissues were dissected from fetal mice, followed by meninges removal and enzymatic dissociation. Dissociated neurons were seeded at appropriate densities onto poly-L-lysine (0.25 mg/mL)-coated culture dishes and maintained in a humidified incubator (37°C, 5% CO₂). Four hours after seeding, the medium was replaced with neuron-specific Neurobasal medium supplemented with B27 (GIBCO) and 1% penicillin/streptomycin (Invitrogen). From day 6 onward, cytosine arabinoside (2.5 μg/mL final concentration) was added to inhibit glial cell proliferation. Half of the medium was refreshed every 3 days.

Primary astrocytes were cultured in astroglial medium (Dulbecco’s modiﬁed eagle medium/Nutrient mixture F-12 (DMEM/F12)) (1:1) (GIBCO) with 10% fetal bovine serum (FBS). Upon reaching ~90% confluency, cultures were subjected to orbital shaking (220 rpm, 1 hour) to remove non-astrocytic cells, thereby enhancing astrocyte purity.

Primary microglia were isolated from postnatal day 0–3 C57BL/6J mice. After meninges removal, brains were minced and digested with 0.25% trypsin. The suspension was filtered through a 70-μm nylon mesh, pelleted, and resuspended in DMEM containing 10% FBS. Cells were seeded onto poly-L-lysine (0.1 mg/mL)-coated flasks. After 3 days, the medium was replaced with DMEM supplemented with 25 ng/mL GM-CSF and 10% FBS. Primary microglial cells were harvested by shaking (200 rpm, 20 min) after 10-12d in culture, and every 3 days thereafter.

At the same time, to verify the purity of different types of cells, we additionally used Western blot analysis to assess the expression of different cell marker proteins (Tuj1 for neurons, Gfap for astrocytes, and Iba1 for microglia) as an indication of cell type purity.

**Cell culture and plasmids transfection**

HEK293T cells were originally from ATCC (Manassas, Virginia, USA) and maintained in the laboratory. These cells were cultured in DMEM supplemented with 10% FBS at 37°C under 5% CO₂. Expression plasmids were constructed as follows: Gnb5 (1-353aa), Gnb5 (1-193aa) and Gnb5 (194-353aa) generated by inserting target genes into pCMV-HA vector; Gnb5 (1-102aa), Gnb5 (103-193aa), Gnb5 (28-102aa) and Gnb5 (28-102aa&S81L) generated by inserting target genes into pCMV-HA-mCherry vector. For transfection, cells at 70-80% confluency were transfected with 2 μg plasmid DNA using TurboFect reagent (Thermo Fisher Scientific, R0531) according to the manufacturer's lipid:DNA ratio recommendations. Medium was replaced with fresh complete DMEM 5 h post-transfection, followed by 24-48 h incubation for transgene expression prior to analysis.

**Rotarod Test**

Mice were tested on an AccuRotor Rota Rod system (Accuscan, Columbus, OH) with a 30-mm diameter rotating dowel positioned 63 cm above the base platform. During testing, the stationary rod was gradually accelerated from 4 to 60 rpm over a 300-sec period. Each session consisted of four consecutive trials with 15-min inter-trial intervals. The latency to fall (seconds) was recorded automatically by infrared sensors. The mean latency to fall across four consecutive trials was calculated for each mouse and recorded as the daily performance. This protocol was repeated across three consecutive days with consistent daily timing (± 1 h). For quantitative behavioral analysis, the arithmetic mean of daily performance measures was computed across the observation period and established as the definitive phenotyping metric for each mouse. All behavioral testing was conducted by experimenters blinded to group assignments.

**Supplementary Table**

| **Table A：Primer sequences used in this study.** | | |
| --- | --- | --- |
| **Gene** | **Primer** | **Primer sequences** |
| Gnas | Forward | CAGAGCCTCCATTGGGGTC |
|  | Reverse | GCTTCTCGCTCAACTGGGG |
| Gnal | Forward | ATGGGCCTATGCTACAGCCT |
|  | Reverse | CTTTCCTCGCCTCTTTAGCCG |
| Gnai1 | Forward | GGTTTACAGACACGTCCATCAT |
|  | Reverse | GCCTGCATATTCTGGGTAGCAT |
| Gnai2 | Forward | CAGAGGAACAAGGGATGCTTC |
|  | Reverse | TAAGCGGCTGAGTCATTGAGC |
| Gnai3 | Forward | GAGCGGAGCAAGATGATCGAC |
|  | Reverse | CGTCCTCTGAATAGCCGTCC |
| Gnao1 | Forward | TGCACGAGTCTCTCATGCTCT |
|  | Reverse | AGATGGTCAAGGGTGACTTCT |
| Gnat1 | Forward | GATGCCCGCACTGTGAAAC |
|  | Reverse | CCAGCGAATACCCGTCCTG |
| Gnat2 | Forward | GGATGGCTACTCACCCGAAG |
|  | Reverse | TGCATAGTCAATGCCTAGTGTG |
| Gnaz | Forward | ATGTCGGCAAAGCTCAGAGG |
|  | Reverse | CCCAGCAGGAGAAGTTTGATTTC |
| Gnaq | Forward | GGTCGGGCTACTCTGACGA |
|  | Reverse | ACTTGTATGGGATCTTGAGCGT |
| Gna11 | Forward | CAACGCGGAGATCGAGAAACA |
|  | Reverse | GCCTGCATGGCGGTAAAGAT |
| Gna12 | Forward | CGGCTGGTCAAGATCCTGC |
|  | Reverse | GCGTCCACAAGAACCCTCG |
| Gna13 | Forward | GTCCAAGGAGATCGACAAATGC |
|  | Reverse | CCAGCACCCTCATACCTTTGA |
| Gna14 | Forward | AGTGGGAAAAGCACCTTTATCAA |
|  | Reverse | TCCCTGATGATCTGGGCATTT |
| Gna15 | Forward | CGCCAGAATCGACCAGGAG |
|  | Reverse | GTAGCCCACACCGTGAATGA |
| Gngt1 | Forward | TCAACATCGAAGACCTGACAGA |
|  | Reverse | ACACAGCCTCCTTTGAGTTCC |
| Gngt2 | Forward | CAGGACCTCAGTGAGAAGGAG |
|  | Reverse | CCTGCTTGGGCCTCTACATAAT |
| Gng2 | Forward | ACCGCCAGCATAGCACAAG |
|  | Reverse | AGTAGGCCATCAAGTCAGCAG |
| Gng3 | Forward | GCACTATGAGTATTGGTCAAGCA |
|  | Reverse | GTGGGCATCACAGTATGTCATC |
| Gng4 | Forward | GGCATGTCTAATAACAGCACCA |
|  | Reverse | CACTGGGATGATGAGGGGG |
| Gng5 | Forward | CTCAACCGCGTGAAGGTTTC |
|  | Reverse | GGTCTGAAGGGATTCGTACTTG |
| Gng7 | Forward | TCAGGTACTAACAACGTCGCC |
|  | Reverse | CAGTAGCCCATCAGGTCTGAC |
| Gng8 | Forward | TCGCATGAAGGTGTCGCAG |
|  | Reverse | CTTGTCGCGGAAGGGATTCTC |
| Gng11 | Forward | CCTGCCCTTCACATCGAGG |
|  | Reverse | TTGTCTCTGCAACTTGACTTCTT |
| Gng12 | Forward | ATGTCCAGCAAGACGGCAAG |
|  | Reverse | GAGGTCGGTATGCCCATCAG |
| Gng13 | Forward | AGAGCCTCAAGTACCAACTGG |
|  | Reverse | CTTCTCTACCCAAGGGTTGTTC |
| Gnb1 | Forward | CTCCTGACACCAGACTGTTTG |
|  | Reverse | CATCCCTTCTCGGACATCCC |
| Gnb2 | Forward | TACACCACTAACAAGGTCCACG |
|  | Reverse | CAGATGTTGTCCAAACCCCCA |
| Gnb3 | Forward | AAGAAGCAGATTGCTGATGCC |
|  | Reverse | GTCCCCTTAATGTCCTCCGTG |
| Gnb4 | Forward | CAGGAGGCTGAACAGCTTCG |
|  | Reverse | GGCCCACGGAGTCCATATTA |
| Gnb5 | Forward | ATGTGCGATCAGACCTTCCTG |
|  | Reverse | GGAGCAGTAGTTGAGTTGTTGAG |
| Bace1 | Forward | GGAACCCATCTCGGCATCC |
|  | Reverse | TCCGATTCCTCGTCGGTCTC |
| Gapdh | Forward | AGGTCGGTGTGAACGGATTTG |
|  | Reverse | TGTAGACCATGTAGTTGAGGTCA |
