# Supplementary figures and images for: Gnb5 is a negative regulator of the BACE1-mediated Aβ generation and ameliorates cognitive deficits in a mouse model of Alzheimer’s disease
Source: PLoS Biol. 2025 Jun 30;23(6):e3003259. doi: 10.1371/journal.pbio.3003259 (PMC12233908; doi:10.1371/journal.pbio.3003259)

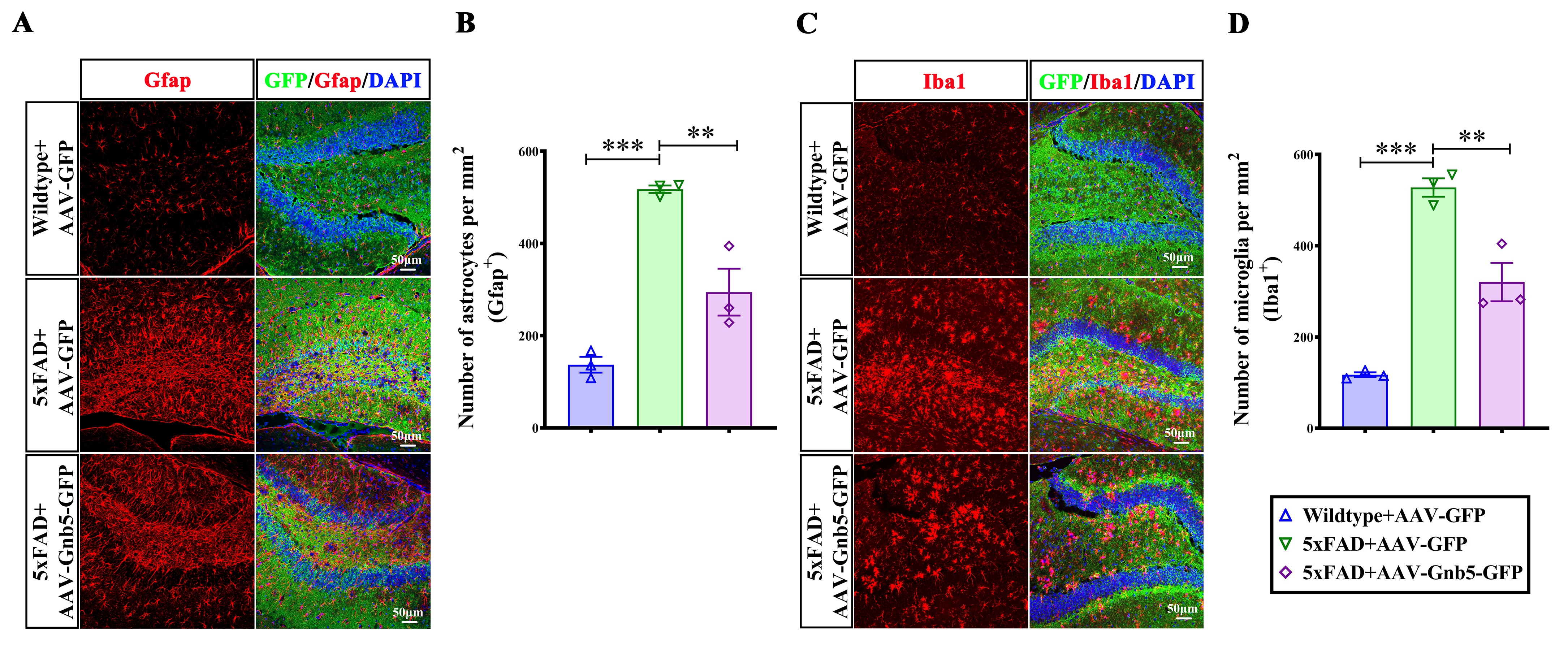

Supplement: S3 Fig — (B, D) Statistical analysis of the number of astrocytes (B) or microglia (D) in the hippocampus of 6-month-old AAV-GFP or AAV-Gnb5-GFP injected Wild-type and 5xFAD mice; n = 3, respectively. (TIF) [file pbio.3003259.s004.tif]

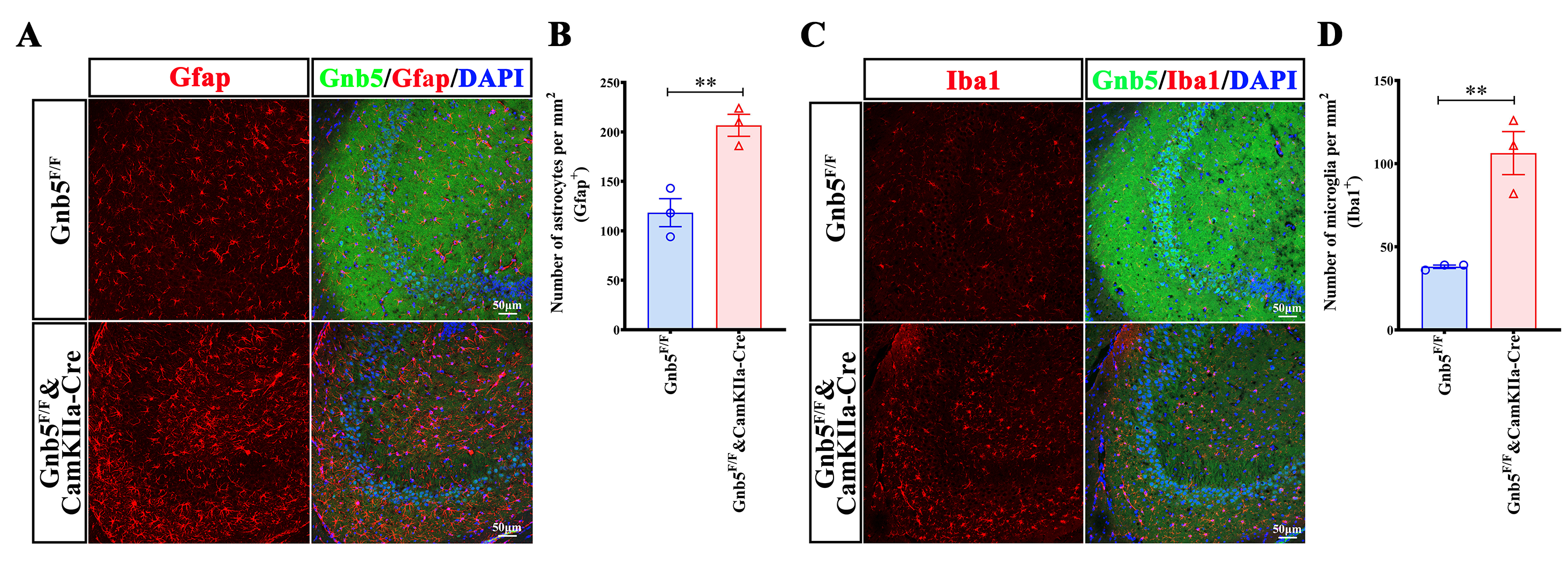

Supplement: S4 Fig — (B, D) Statistical analysis of the number of astrocytes (B) or microglia (D) in the hippocampus of 6-month-old Gnb5F/F and Gnb5-CCKO mice; n = 3, respectively. (TIF) [file pbio.3003259.s005.tif]

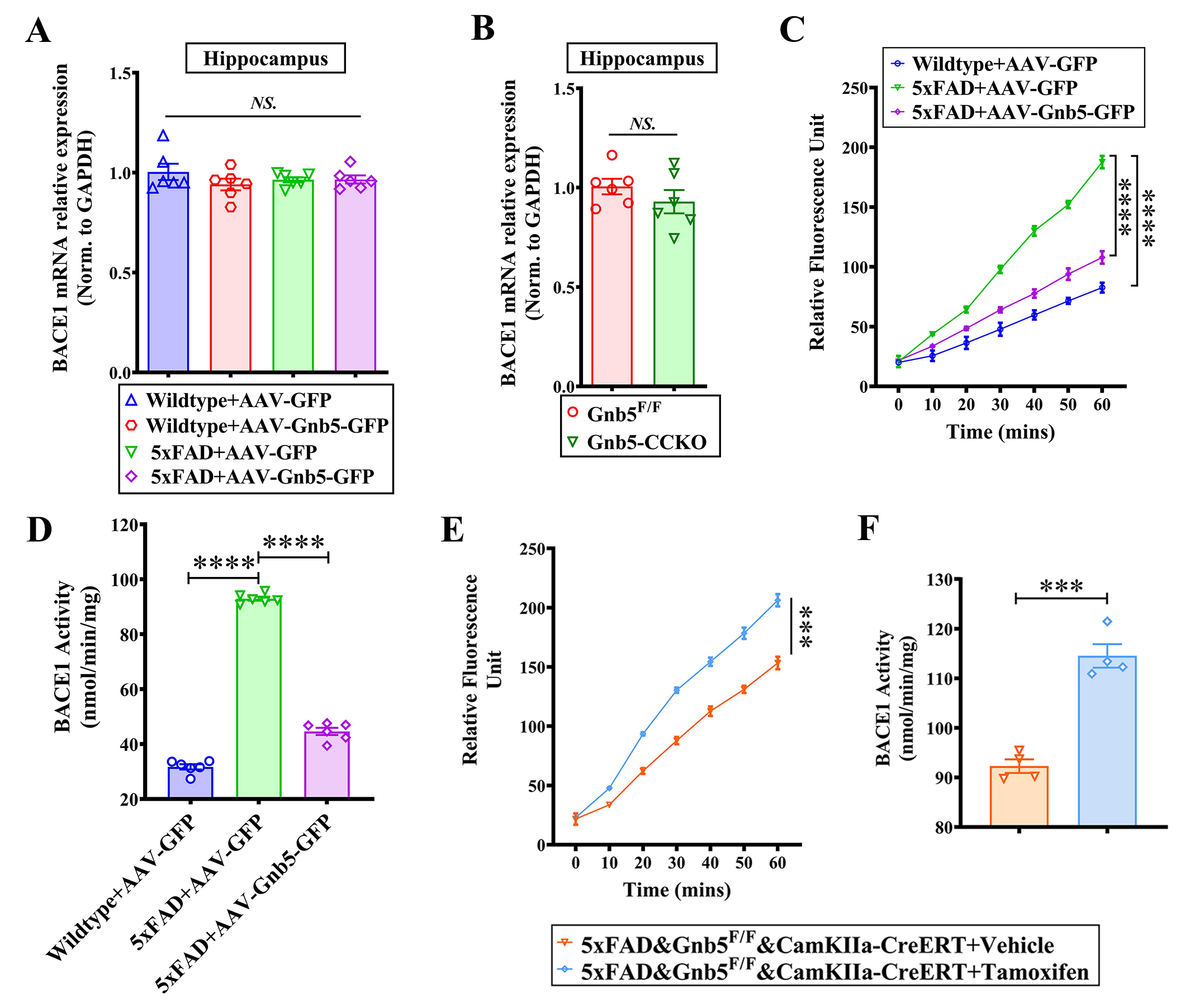

Supplement: S5 Fig — (A) Overexpression of Gnb5-AAV in the hippocampus to detect BACE1 mRNA expression; n = 6, respectively; (B) The mRNA expression level of BACE1 in the hippocampus of Gnb5-CCKO mice; n = 6, respectively; (C) Fluorescence intensity of BACE1-mediated fluorogenic products was measured in hippocampal tissues from 5xFAD mice receiving AAV-mediated Gnb5 overexpression; Kinetic readings were recorded at 10-min intervals over a 60-min assay period; Information including the average (Avg), SEM, and sample size; (D) Quantitative analysis of BACE1 enzymatic activity (nmol/min/mg) from panel C; n = 6 mice/group; (E) Tamoxifen-inducible Gnb5 knockdown was performed in hippocampal tissues of 5xFAD mice, with fluorescence intensity of BACE1-generated products quantified every 10 min for 60 min; Information including the Avg, SEM, and sample size; (F) Quantitative analysis of BACE1 enzymatic activity (nmol/min/mg) from panel E; n = 4 mice/group. (TIF) [file pbio.3003259.s006.tif]

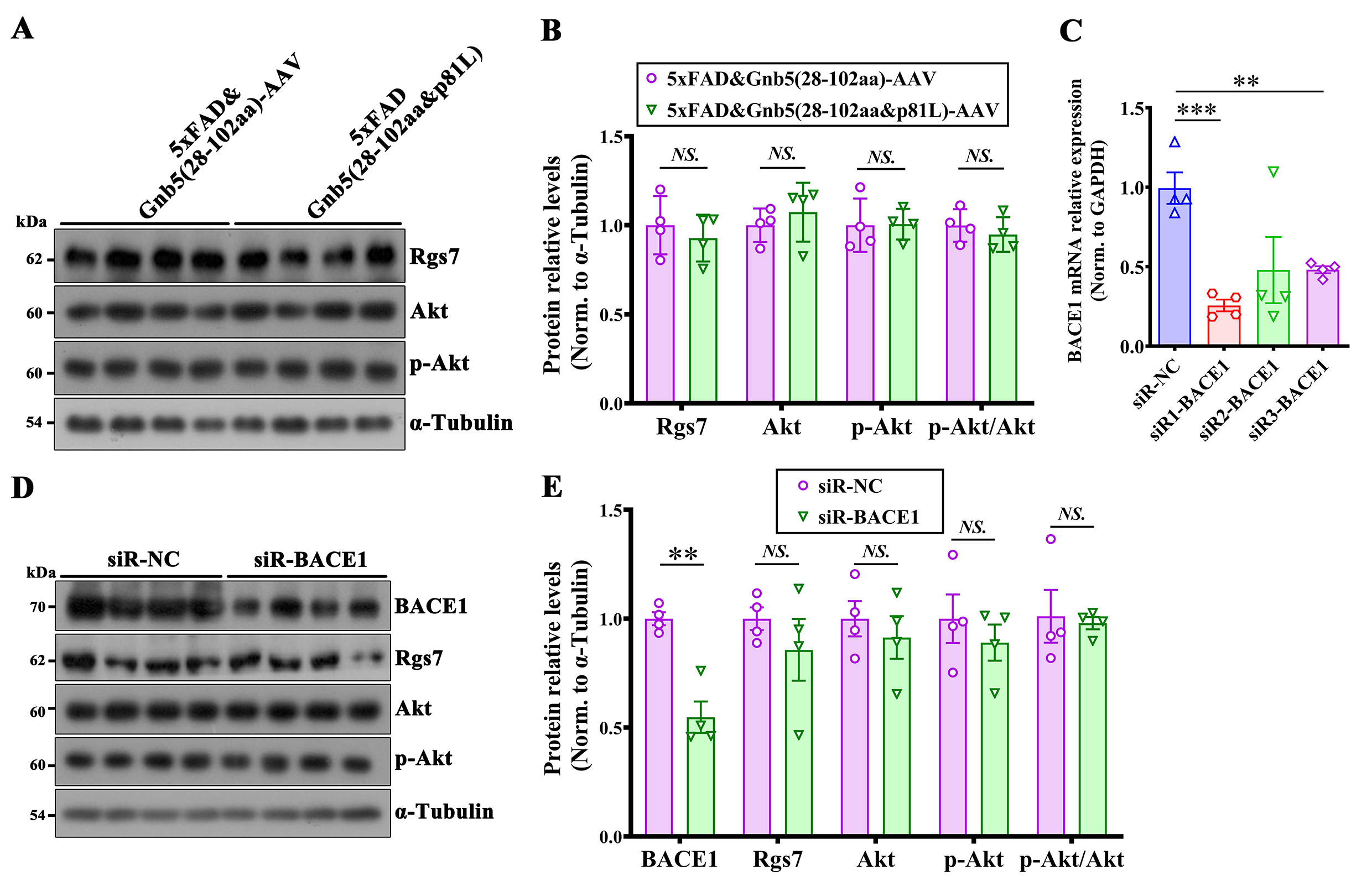

Supplement: S6 Fig — (B) The effects of viral-delivered Gnb5 truncated fragments or mutant truncated fragments on Rgs7, Akt and p-Akt expression in 5xFAD mice; Quantitative analysis of the western blot data shown in panel A; n = 4, respectively; (C) qPCR analysis was performed to evaluate the knockdown efficiencies of three BACE1-targeting siRNAs in HEK293T cells; n = 4, respectively; (E) HEK293T cells transfected with siR1-BACE1 to assess the protein levels of BACE1, Rgs7, Akt and p-Akt; Quantitative analysis of the western blot data shown in panel D; n = 4, respectively. (TIF) [file pbio.3003259.s007.tif]
