# Supplementary material for: Gnb5 is a negative regulator of the BACE1-mediated Aβ generation and ameliorates cognitive deficits in a mouse model of Alzheimer’s disease
Source: PLoS Biol. 2025 Jun 30;23(6):e3003259. doi: 10.1371/journal.pbio.3003259 (PMC12233908; doi:10.1371/journal.pbio.3003259)

Unedited blot and gel images

Figure 1

Figure 1.G- Gnb5 (Hippocampus, 3 months)

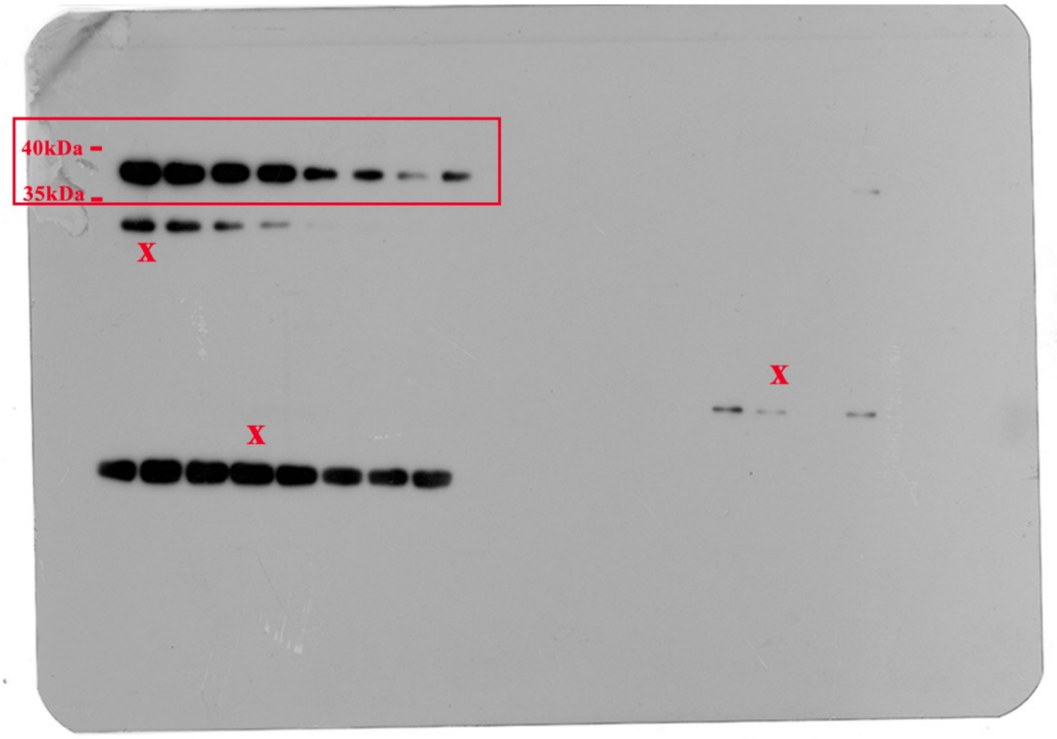

Figure 1.G- APP (Hippocampus, 3 months)

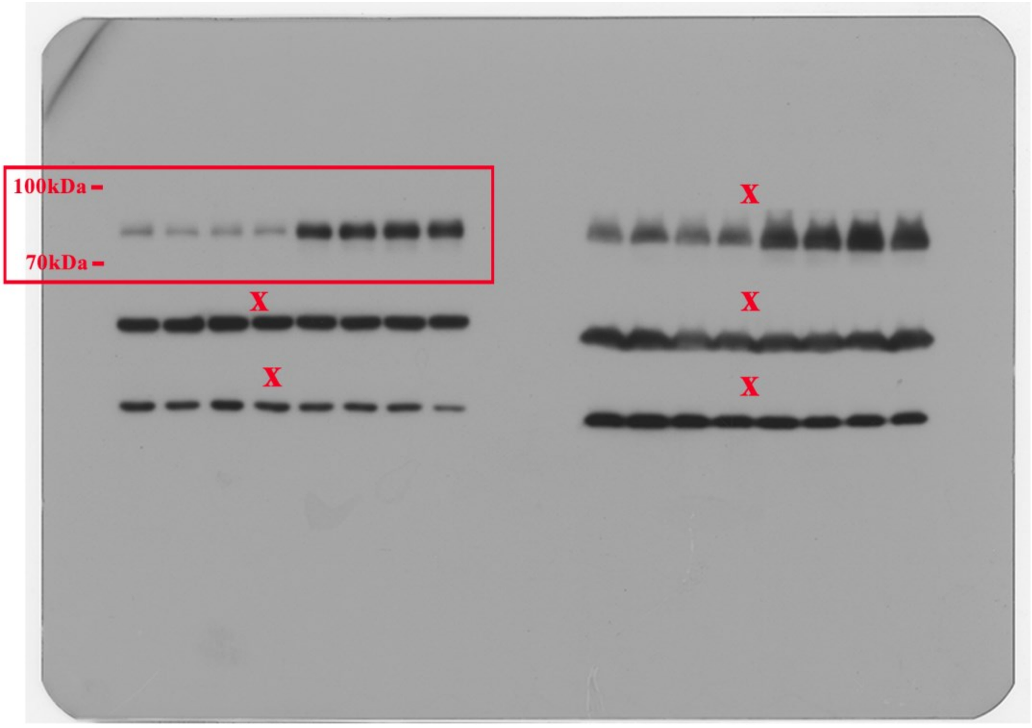

Figure 1.G- α-Tubulin (Hippocampus, 3 months)

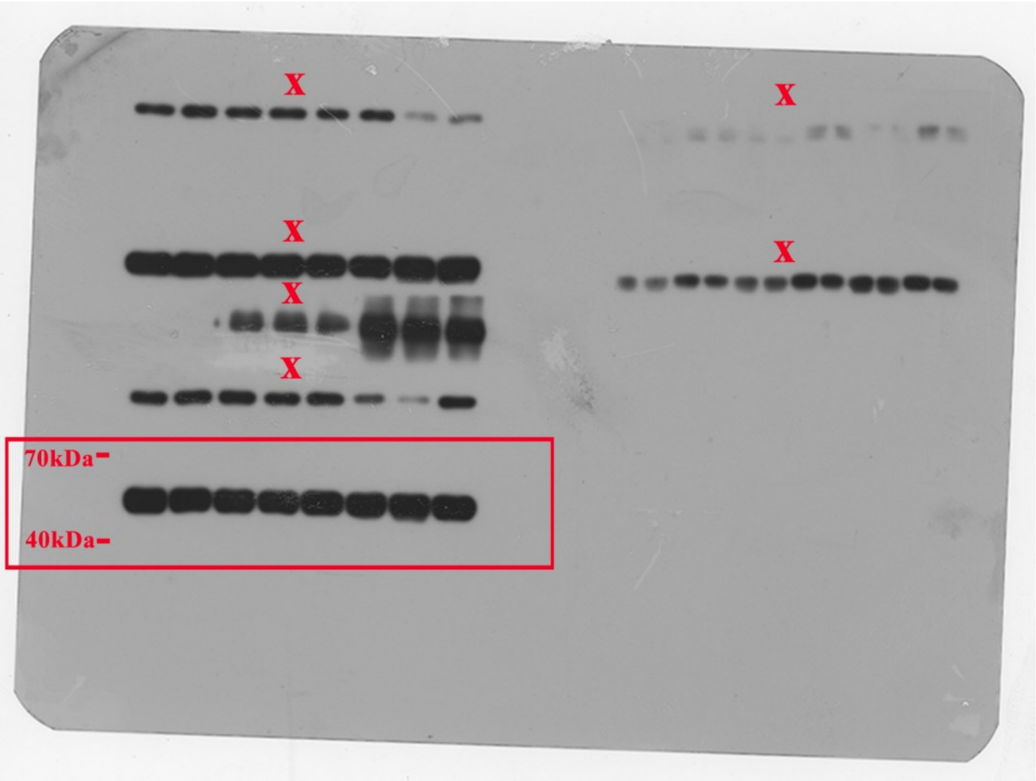

Figure 1.I- Gnb5 (Hippocampus, 6 months)

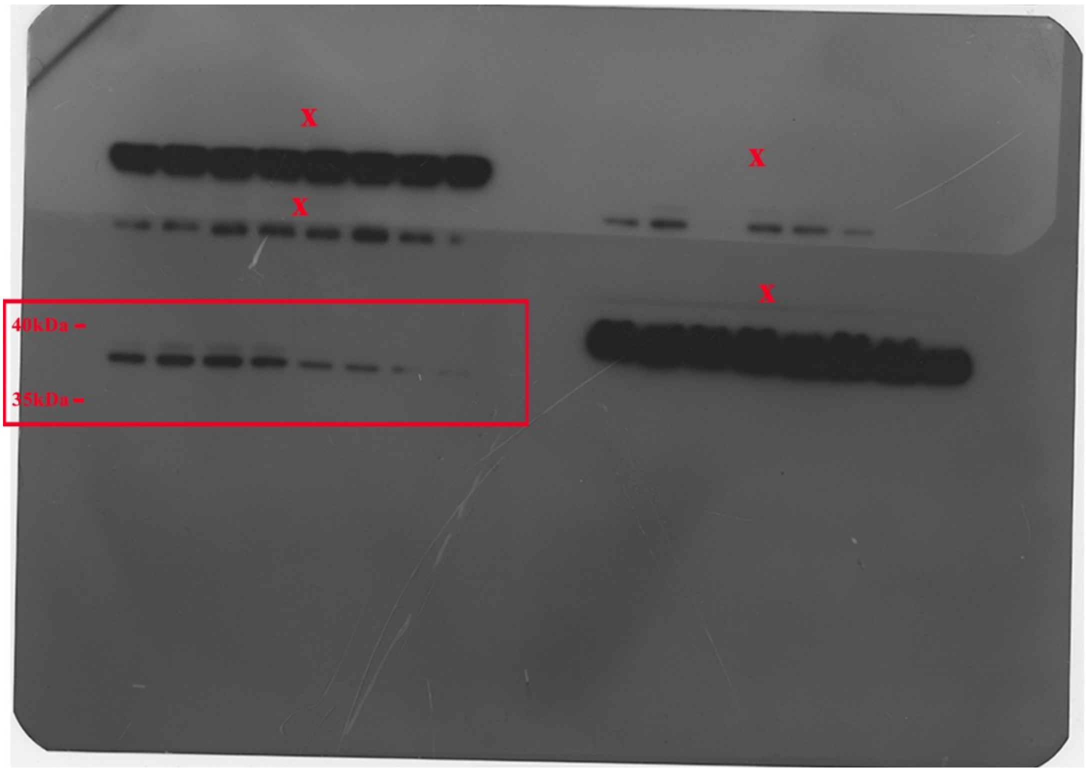

Figure 1.I- APP (Hippocampus, 6 months)

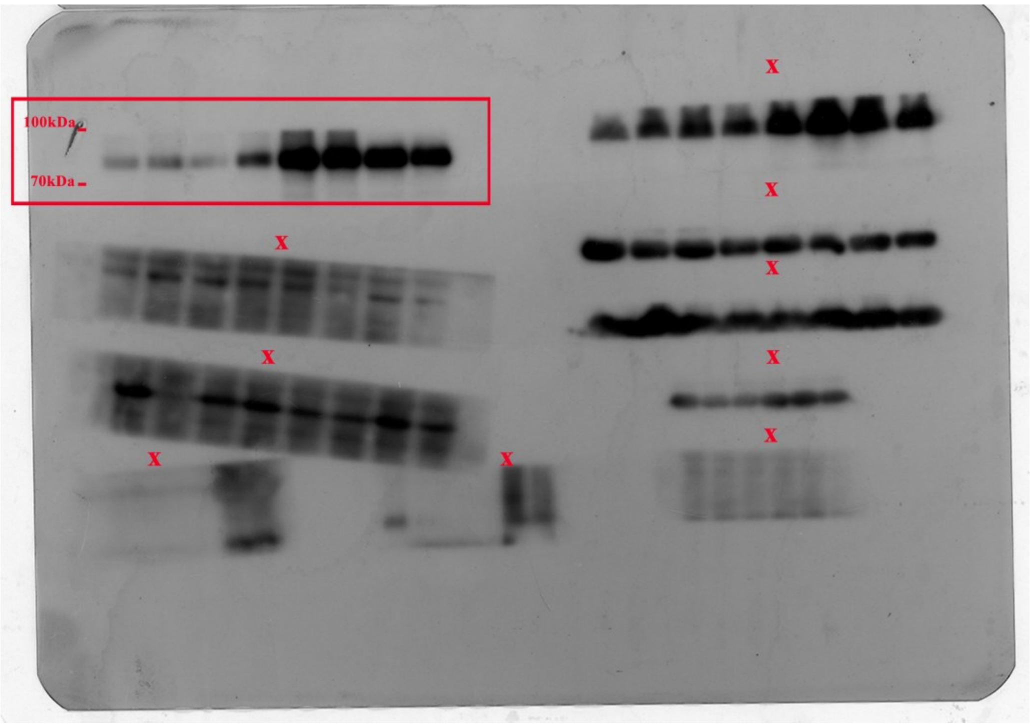

Figure 1.I- α-Tubulin (Hippocampus, 6 months)

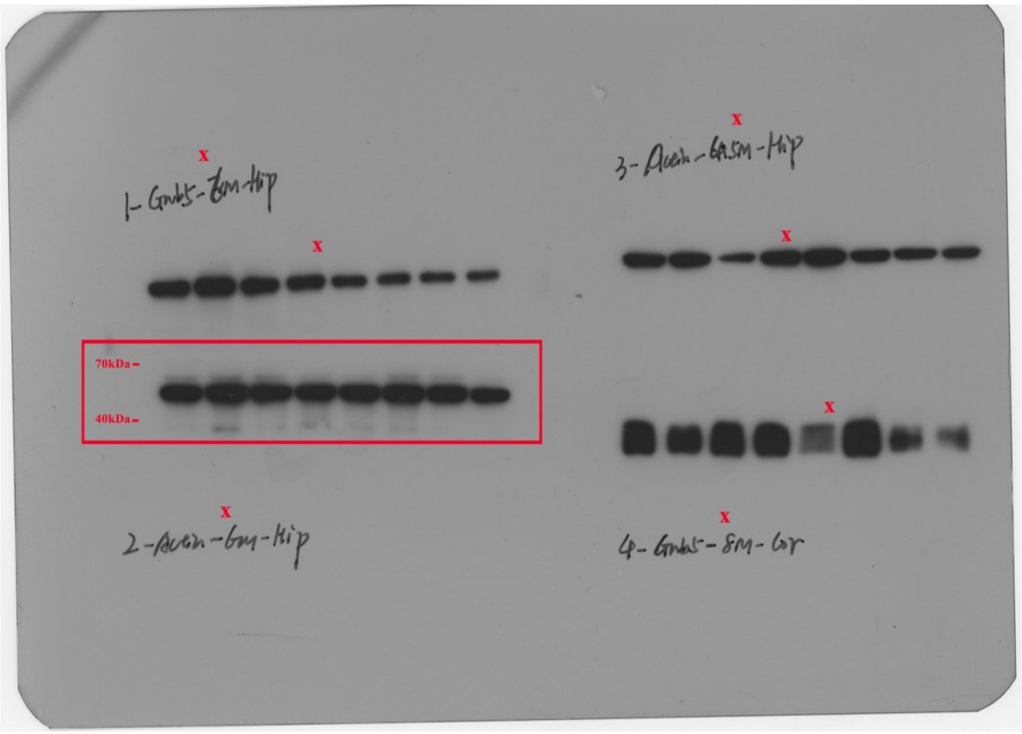

Figure 2

Figure 2.C- Gnb5 (Hippocampus, 3 months)

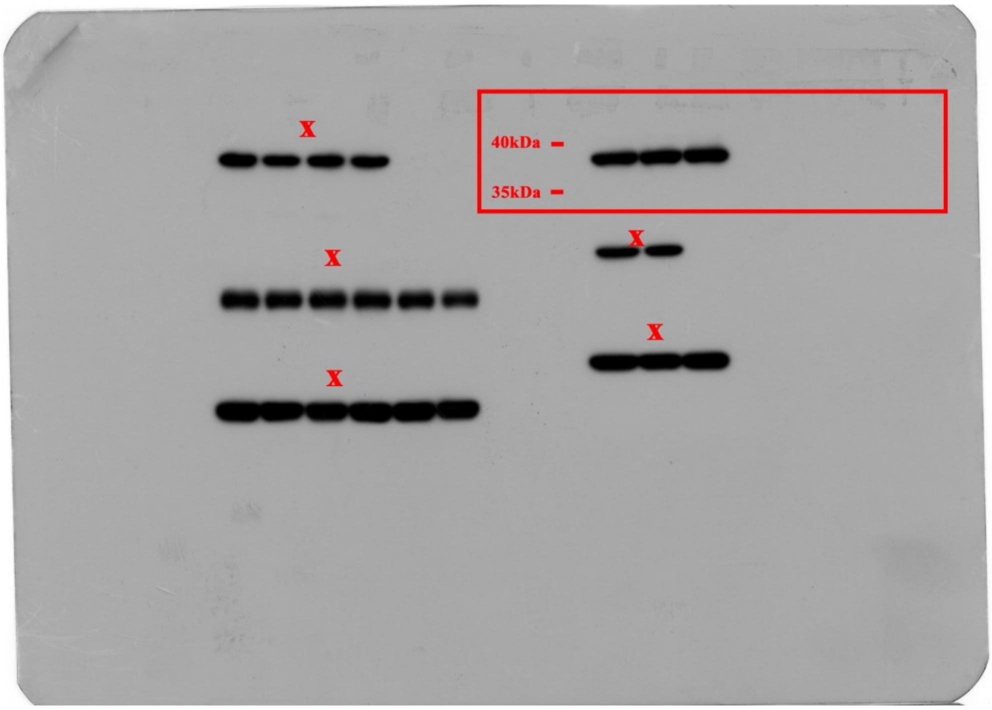

Figure 2.C- α-Tubulin (Hippocampus, 3 months)

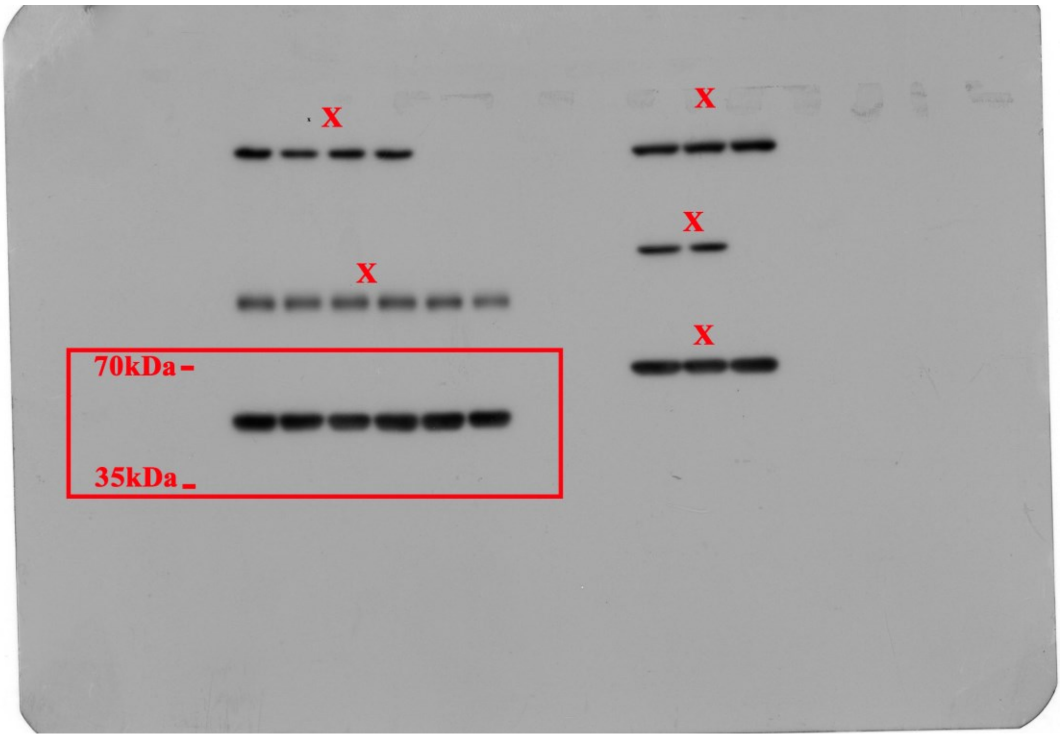

Figure 3

Figure 3.B- Gnb5 (Hippocampus, 6 months)

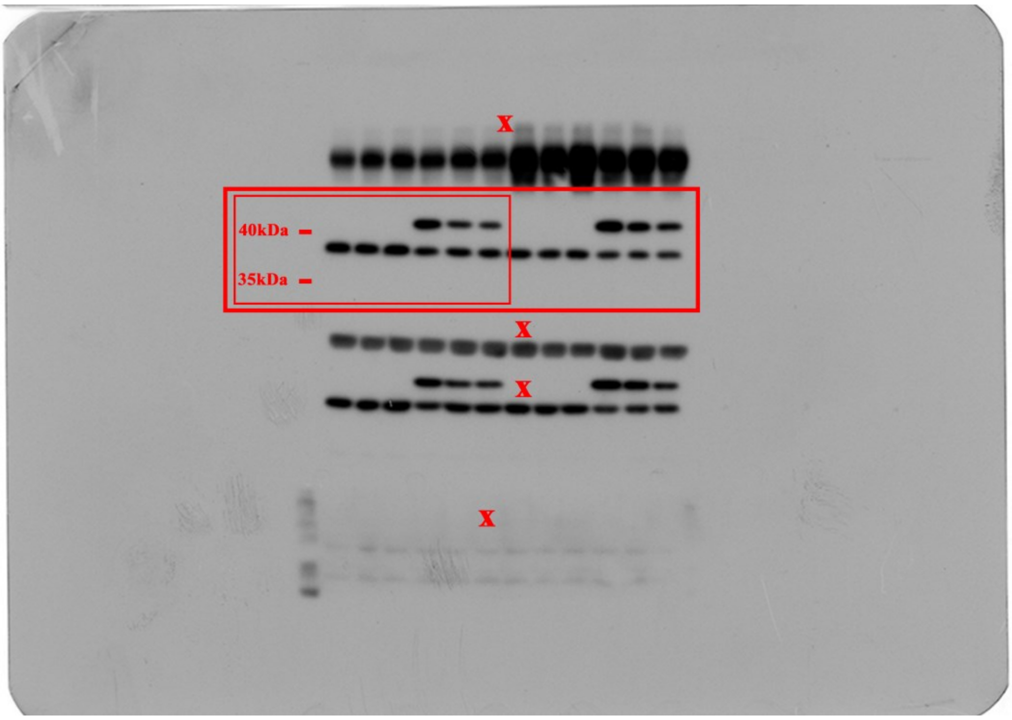

Figure 3.B- α-Tubulin (Hippocampus, 6 months)

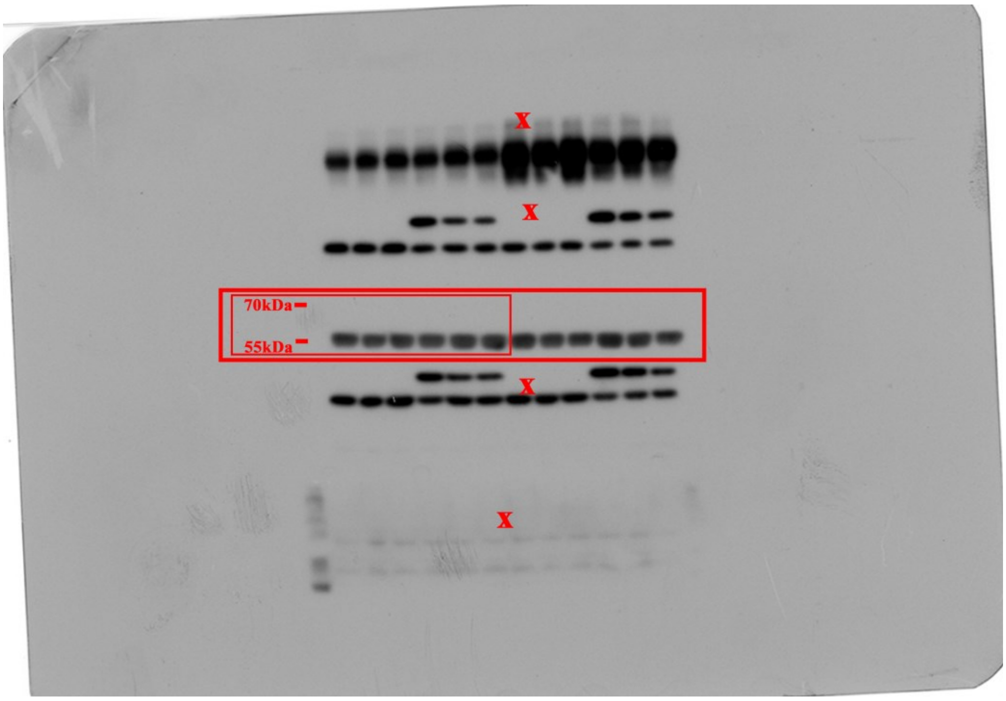

Figure 5

Figure 5.A- Gnb5 (Hippocampus, 6 months)

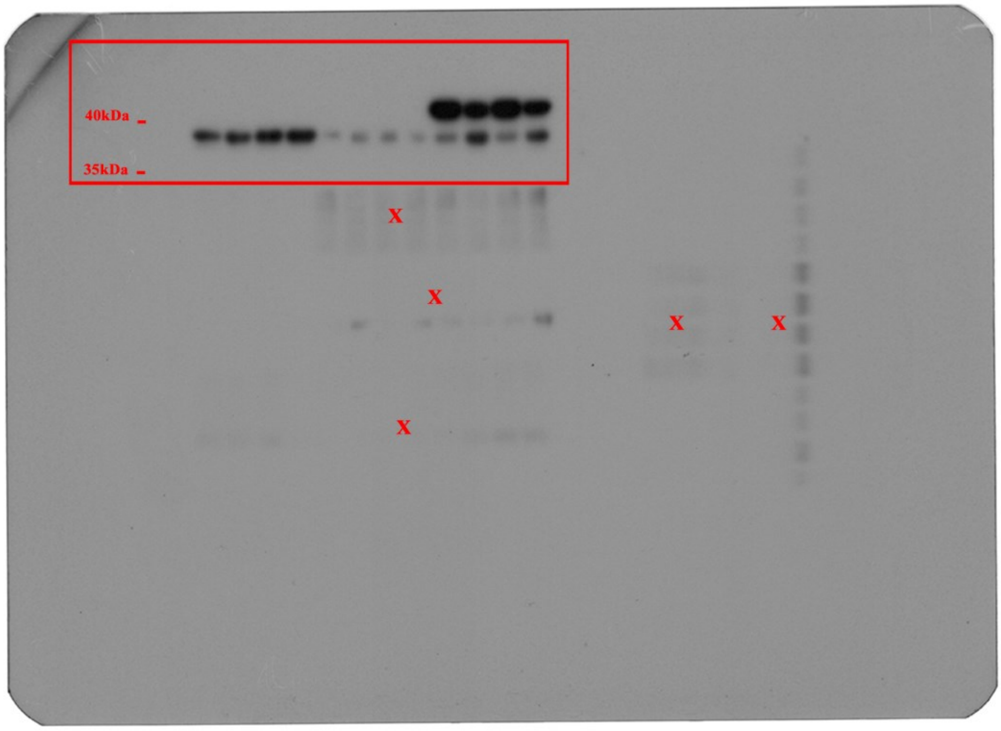

Figure 5.A- APP (Hippocampus, 6 months)

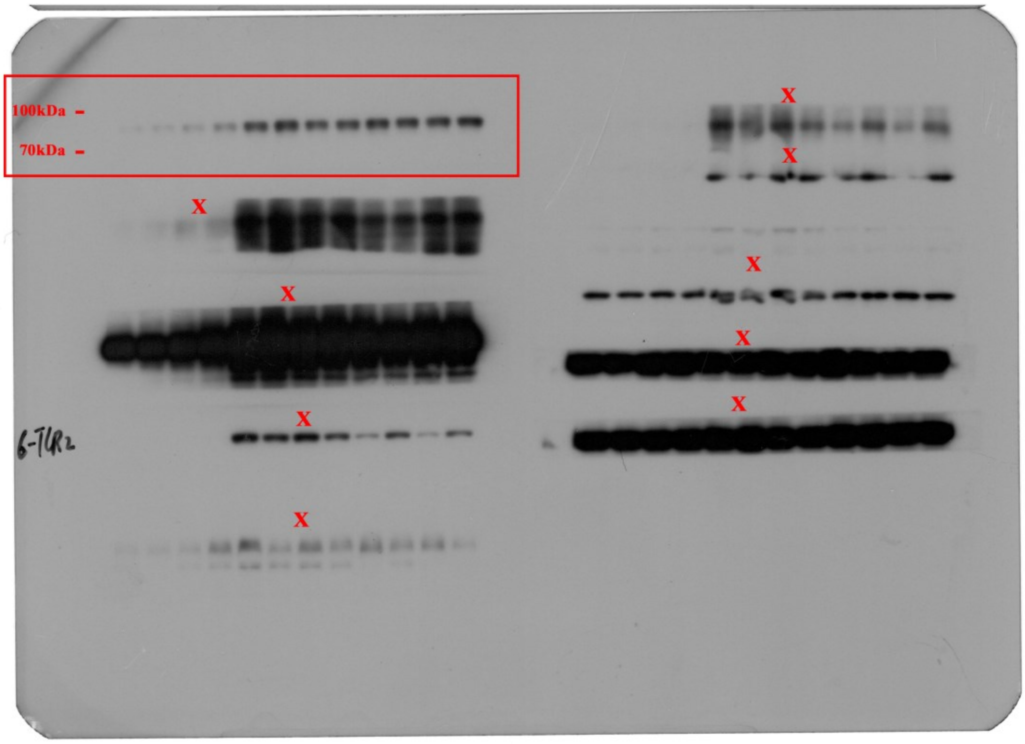

Figure 5.A- Nicastrin (Hippocampus, 6 months)

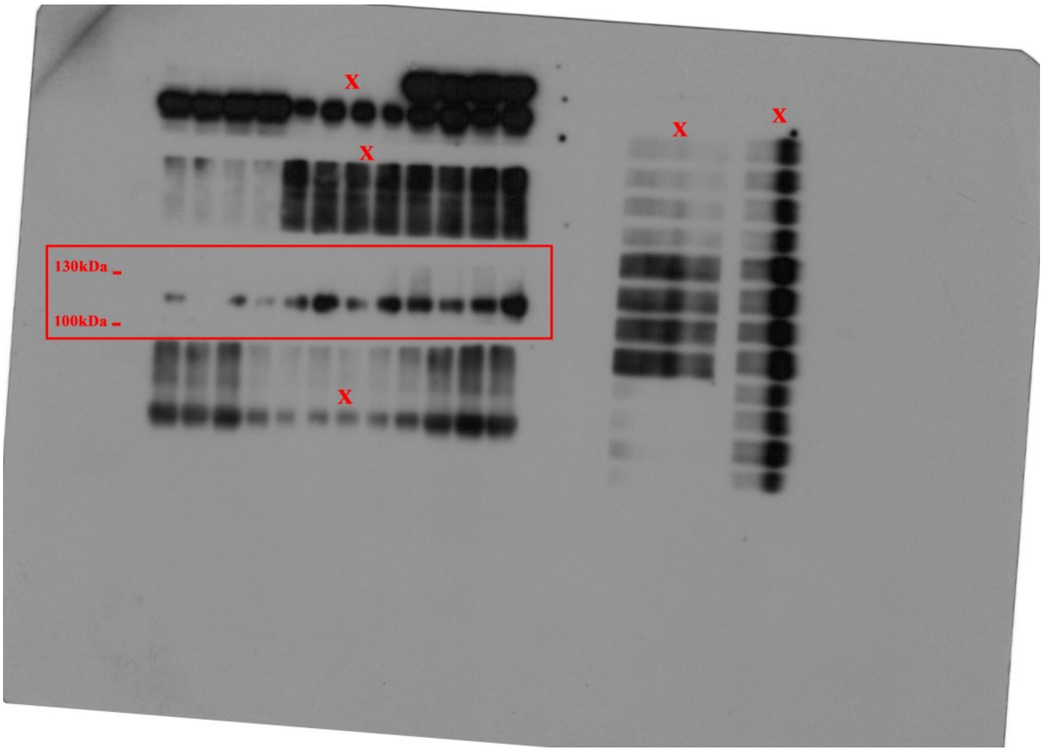

Figure 5.A- BACE1 (Hippocampus, 6 months)

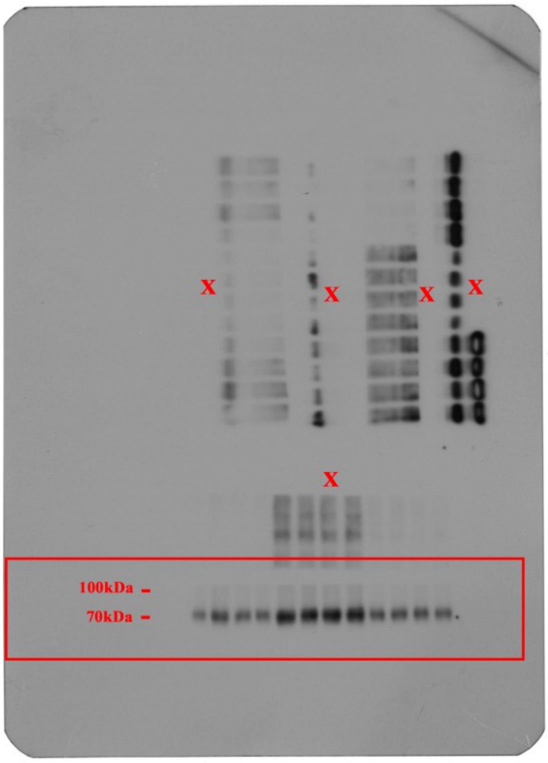

Figure 5.A- β-CTF (Hippocampus, 6 months)

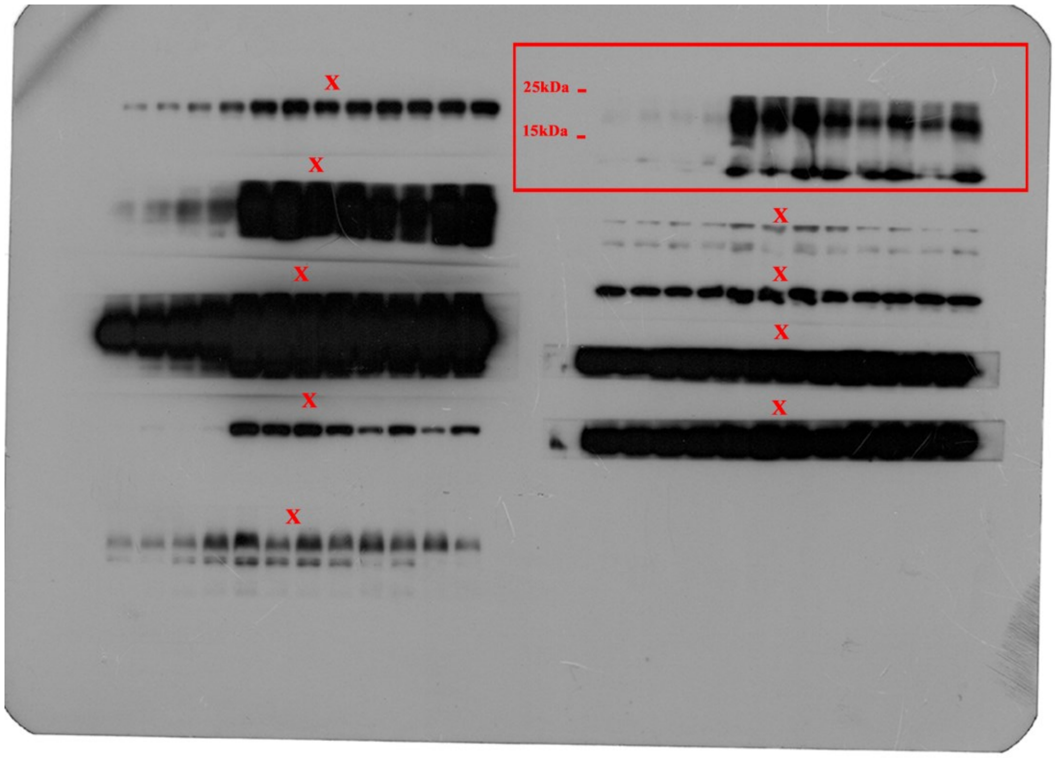

Figure 5.A- α-Tubulin (Hippocampus, 6 months)

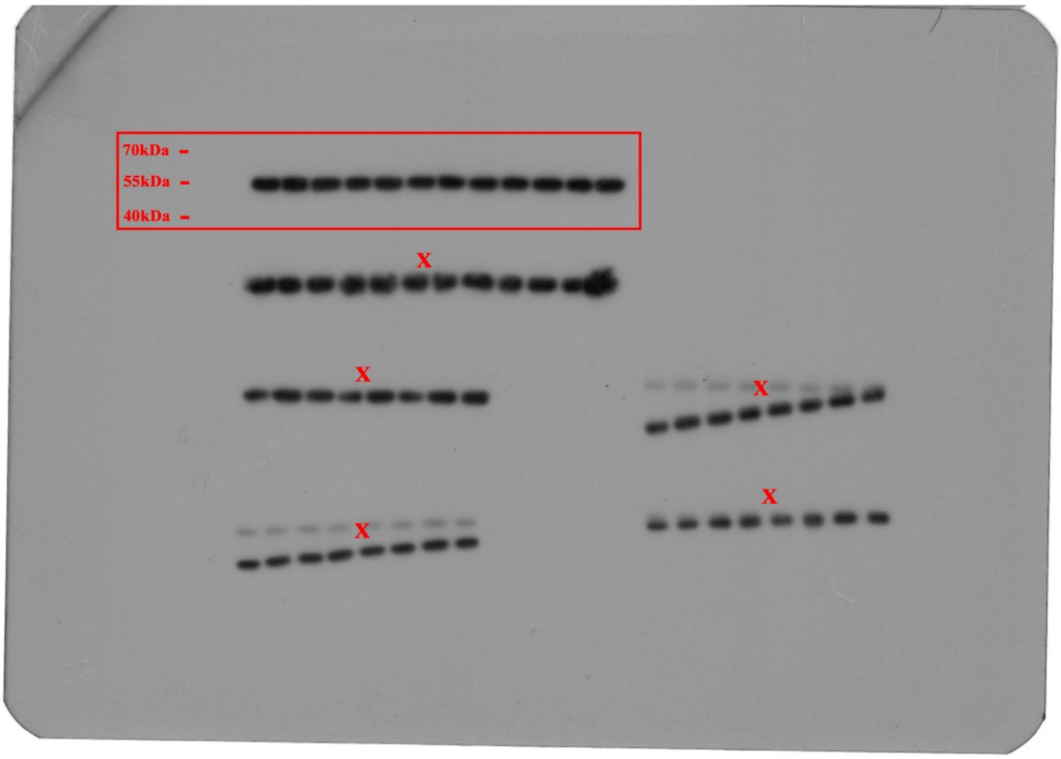

Figure 5.C- Gnb5 (Hippocampus, 3 months)

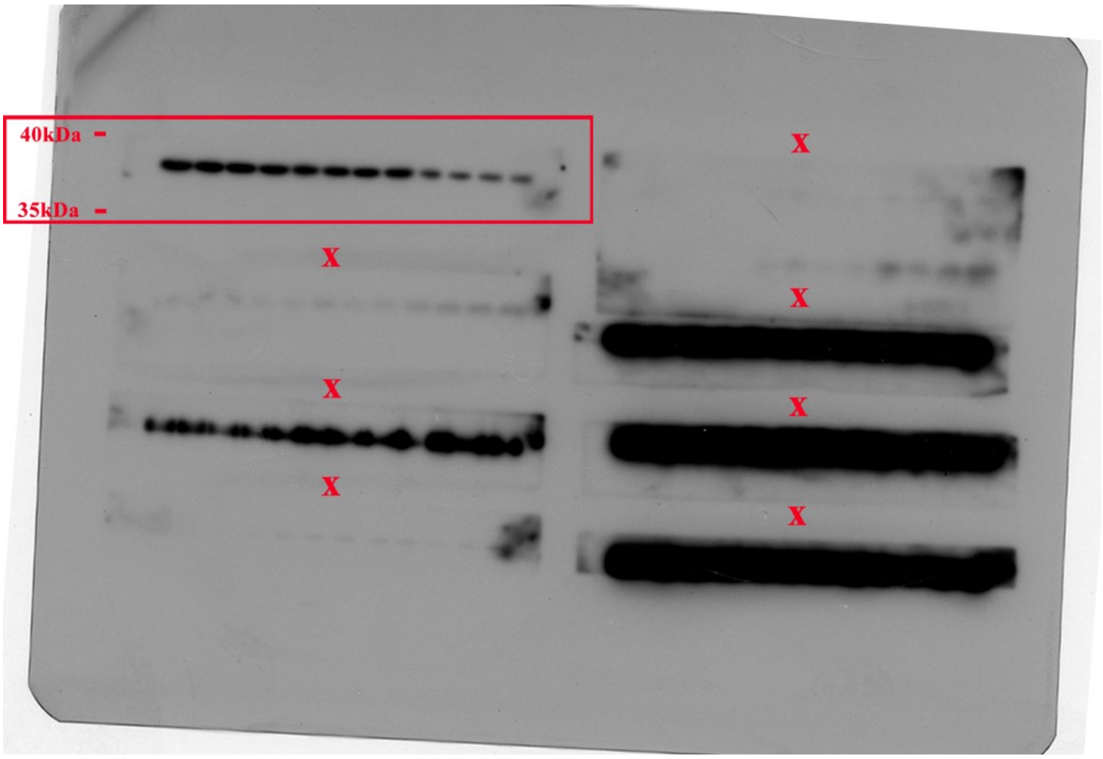

Figure 5.C- BACE1 (Hippocampus, 3 months)

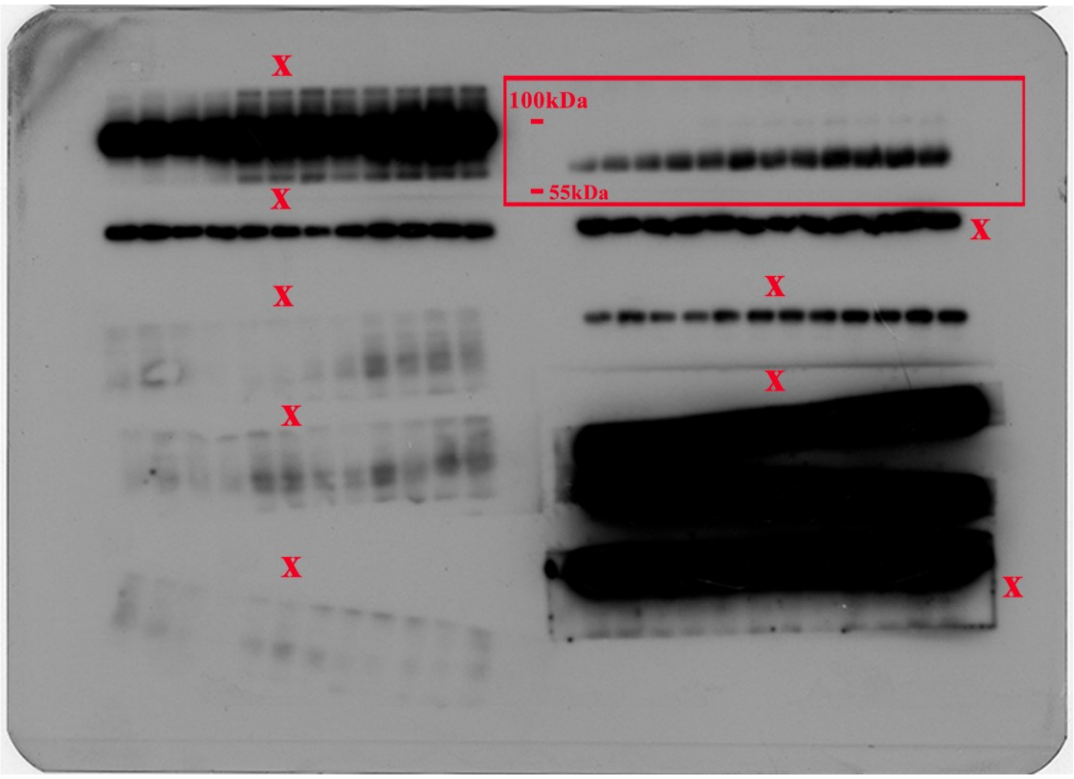

Figure 5.C- β-CTF (Hippocampus, 3 months)

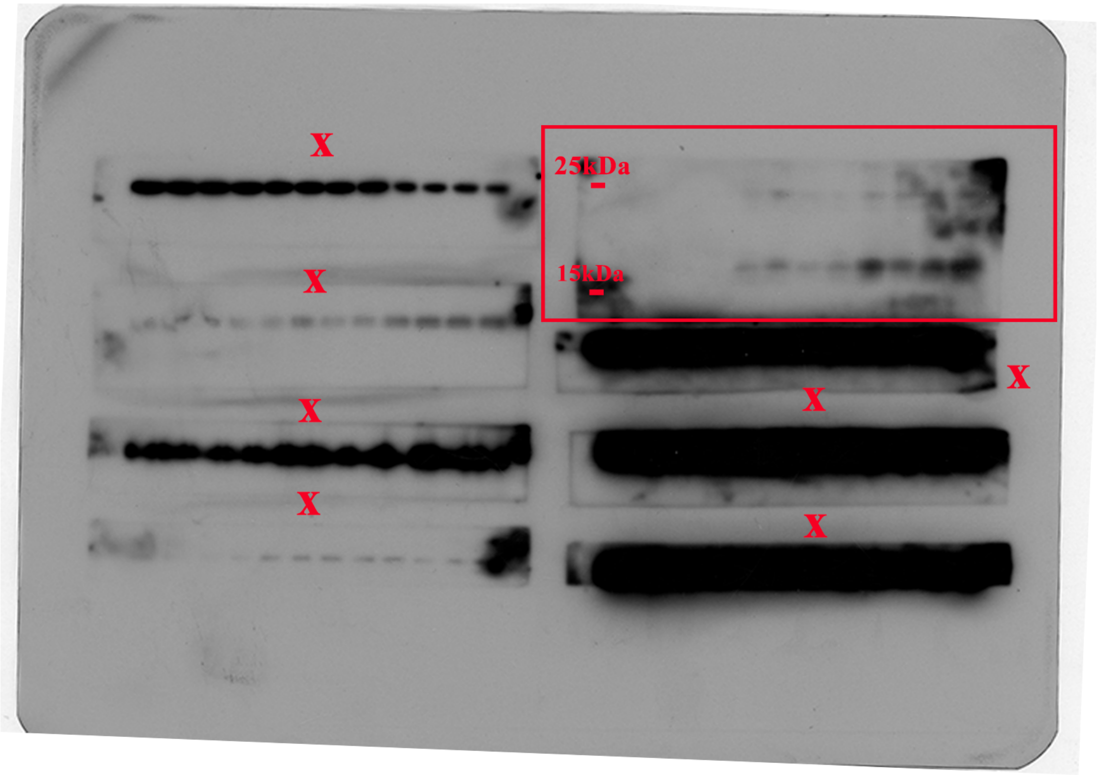

Figure 5.C- Nicastrin (Hippocampus, 3 months)

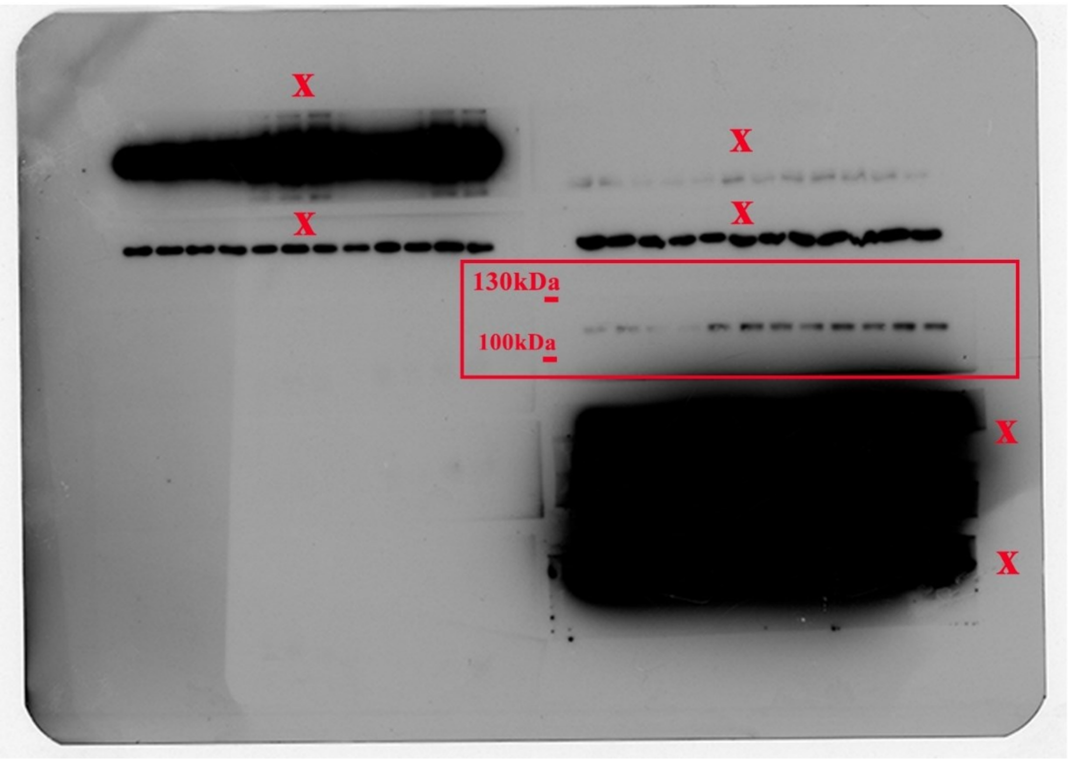

Figure 5.C- APP (Hippocampus, 3 months)

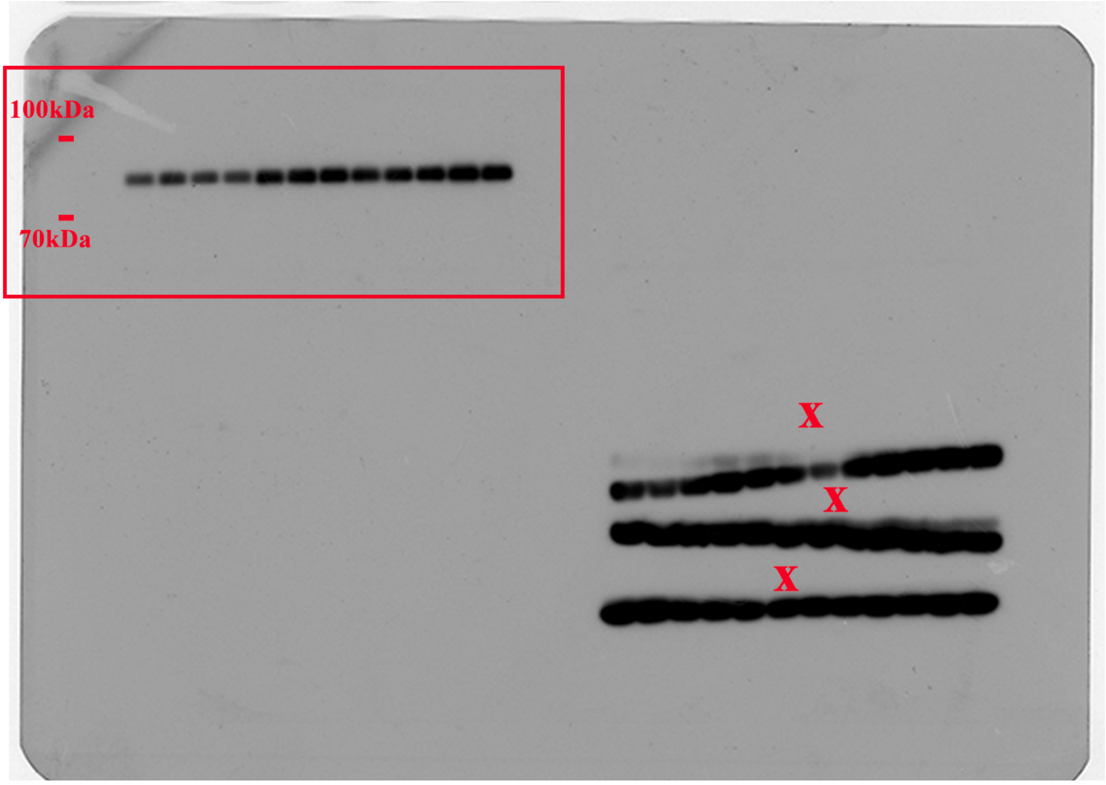

Figure 5.C- α-Tubulin (Hippocampus, 3 months)

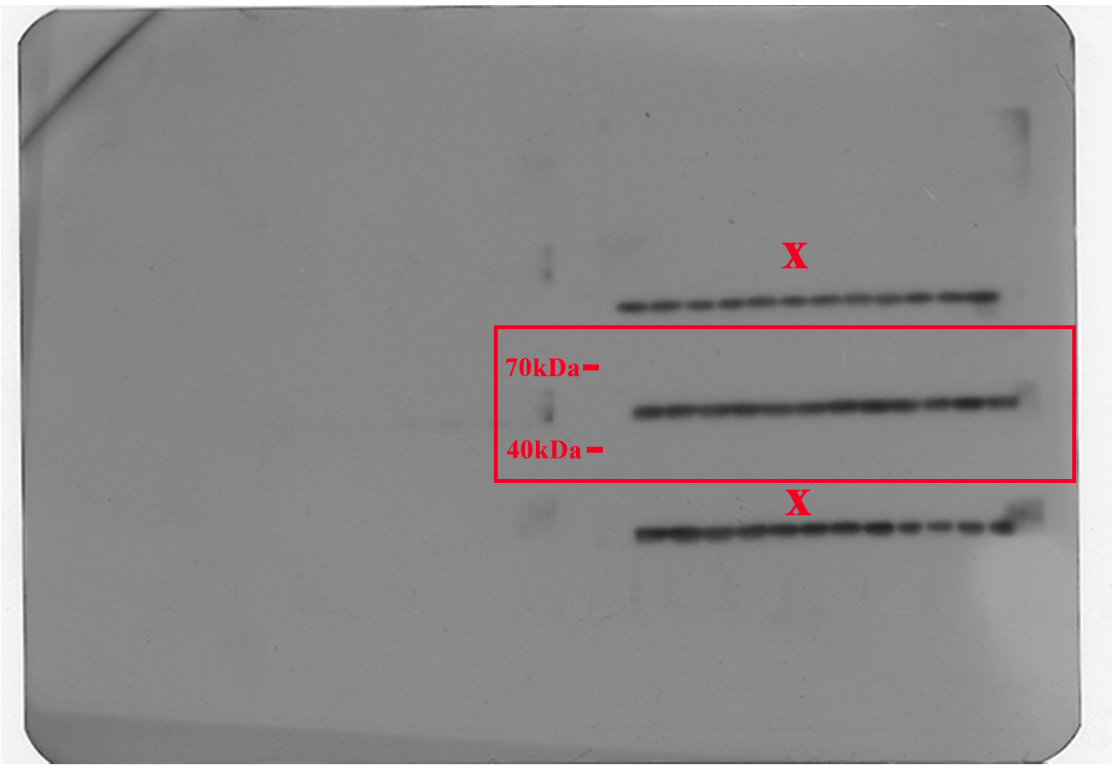

Figure 5.E- Gnb5 (Hippocampus, 6 months)

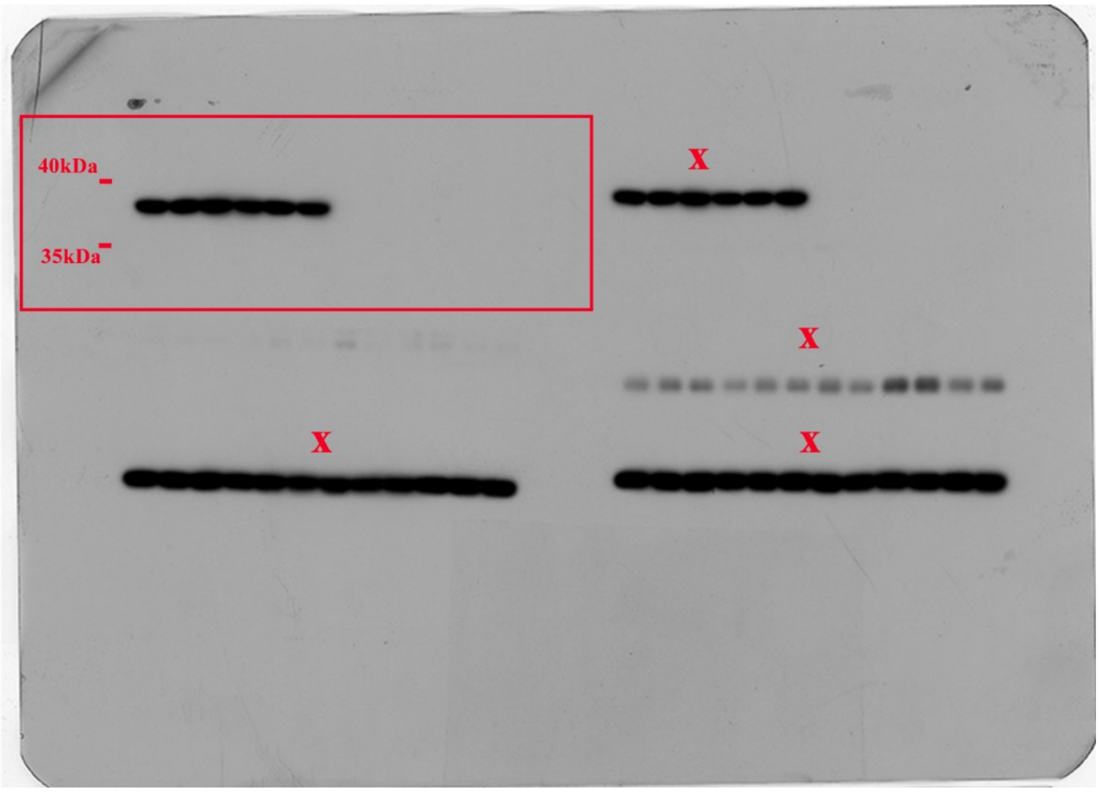

Figure 5.E- APP (Hippocampus, 6 months)

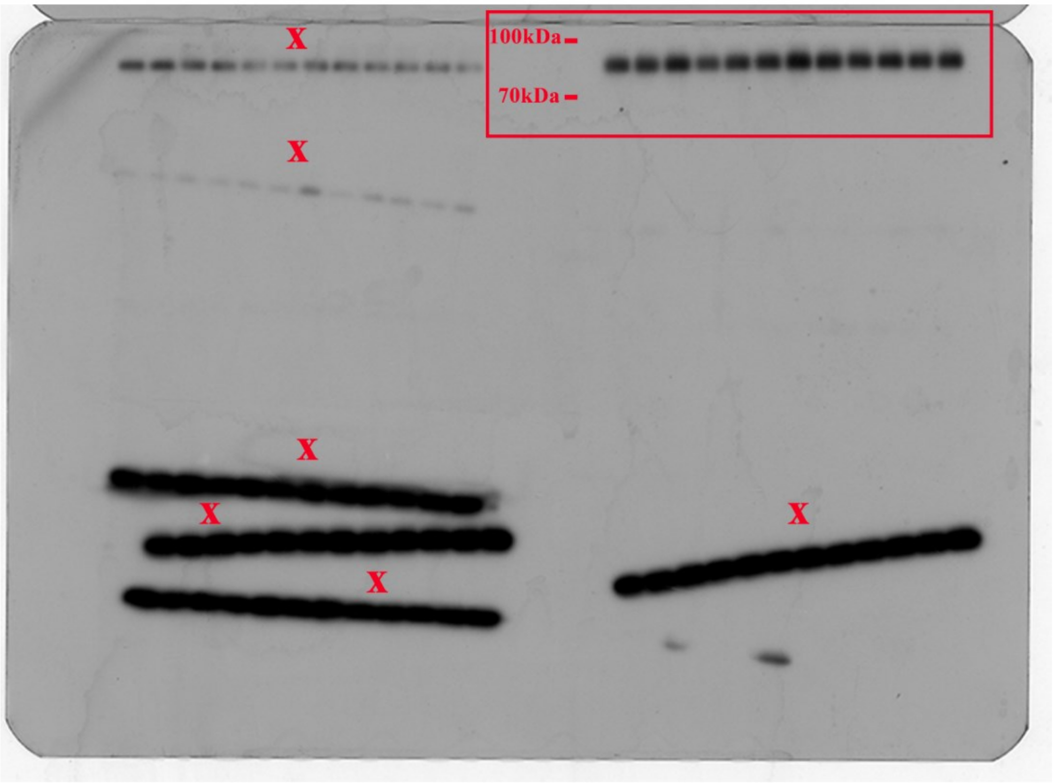

Figure 5.E- Nicastrin (Hippocampus, 6 months)

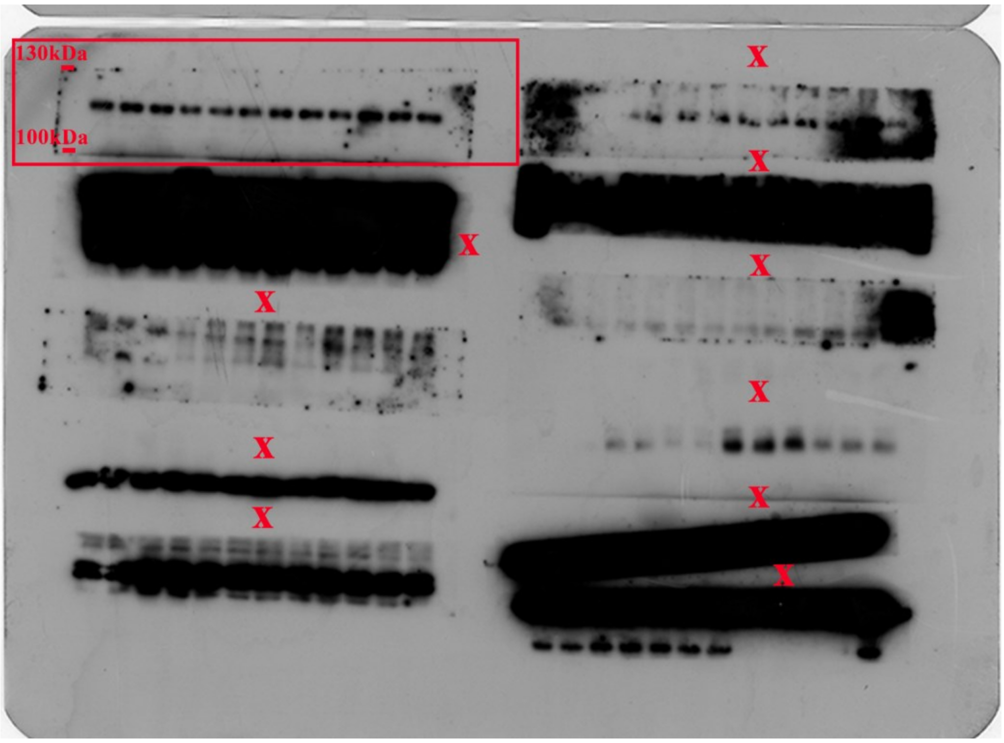

Figure 5.E- BACE1 (Hippocampus, 6 months)

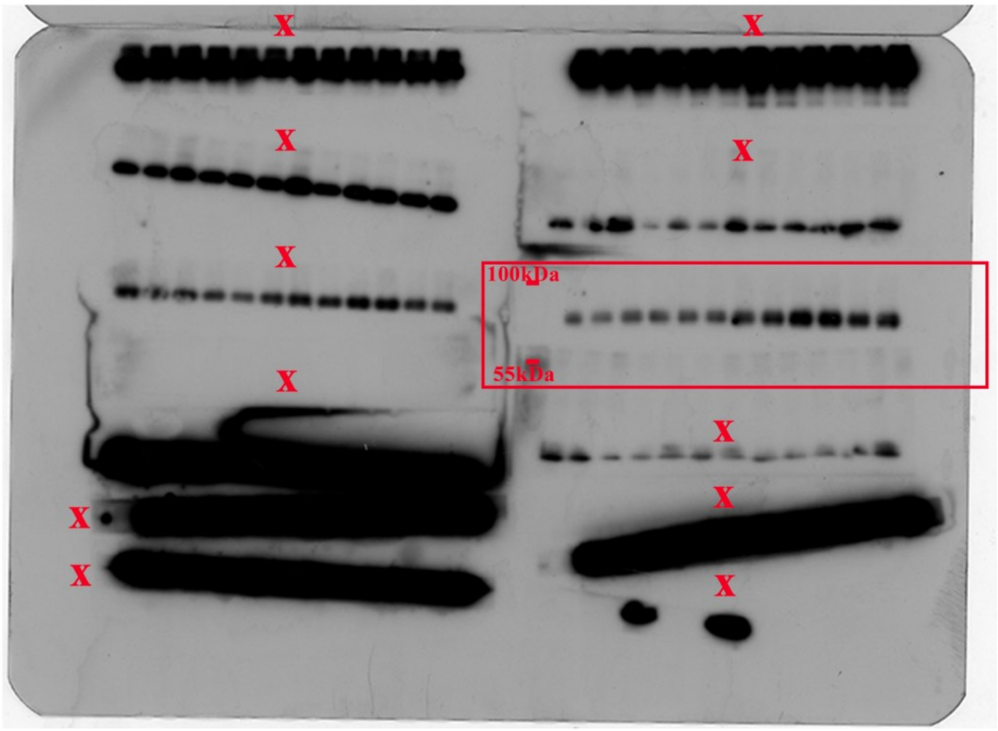

Figure 5.E- β-CTF (Hippocampus, 6 months)

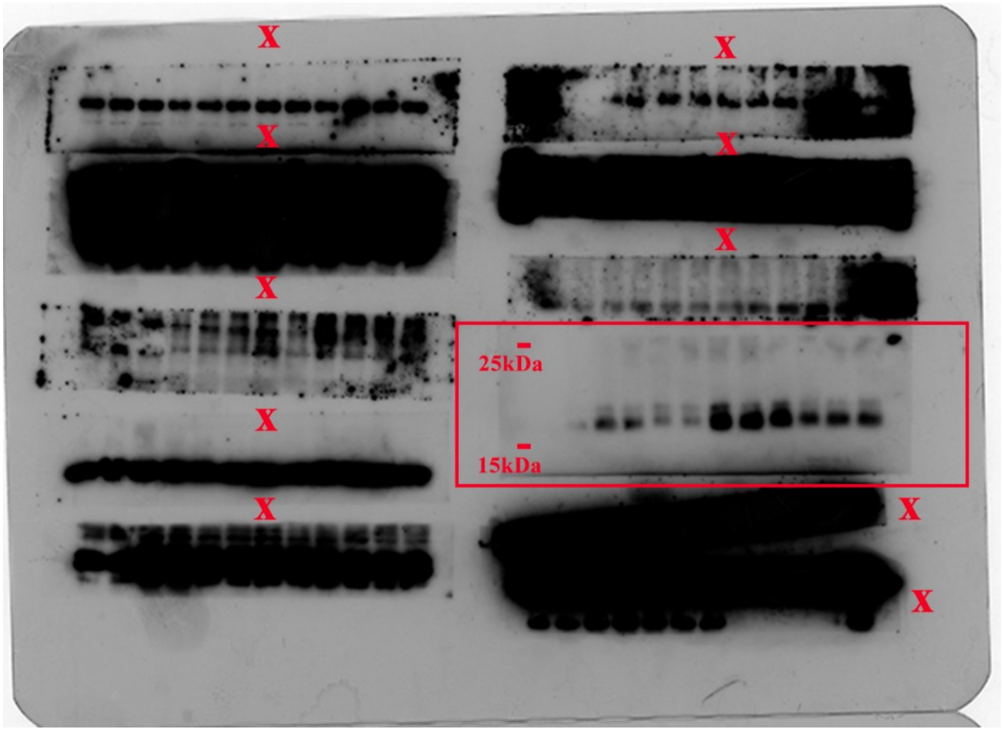

Figure 5.E- α-Tubulin (Hippocampus, 6 months)

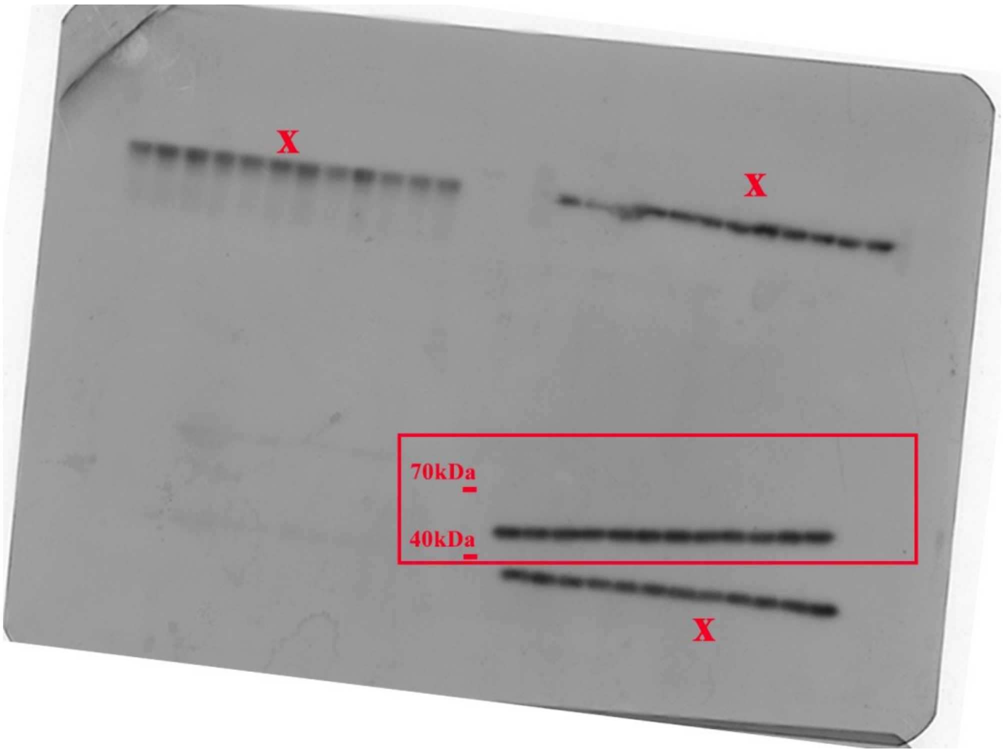

Figure 6

Figure 6.A-BACE1 (IP:Gnb5, Hippocampus)

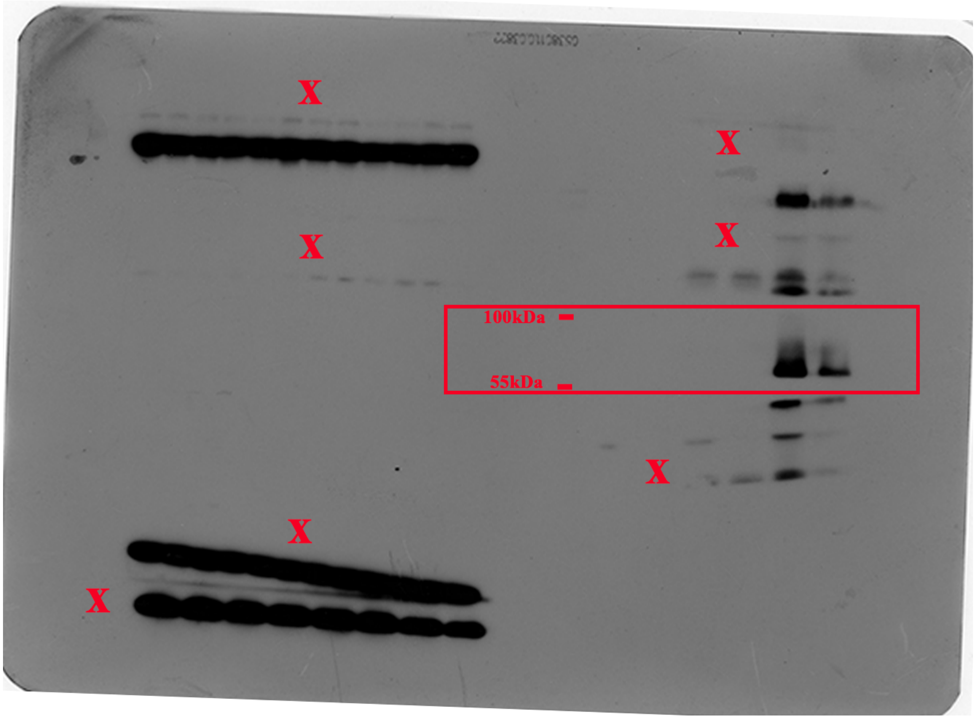

Figure 6.A-Gnb5 (IP:Gnb5, Hippocampus)

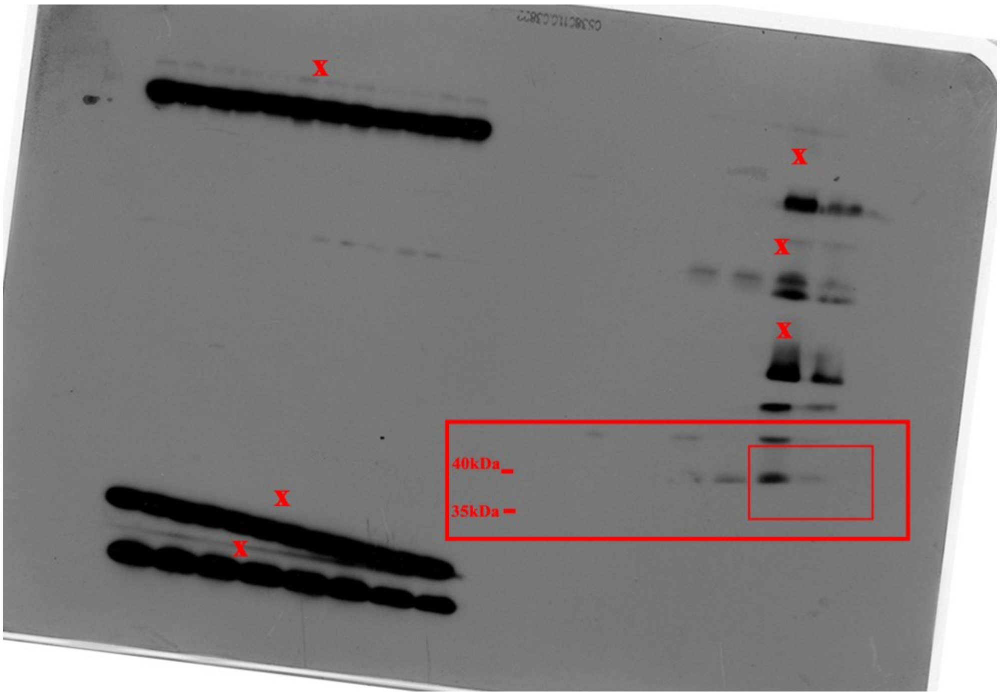

Figure 6.A-BACE1 (Input, Hippocampus)

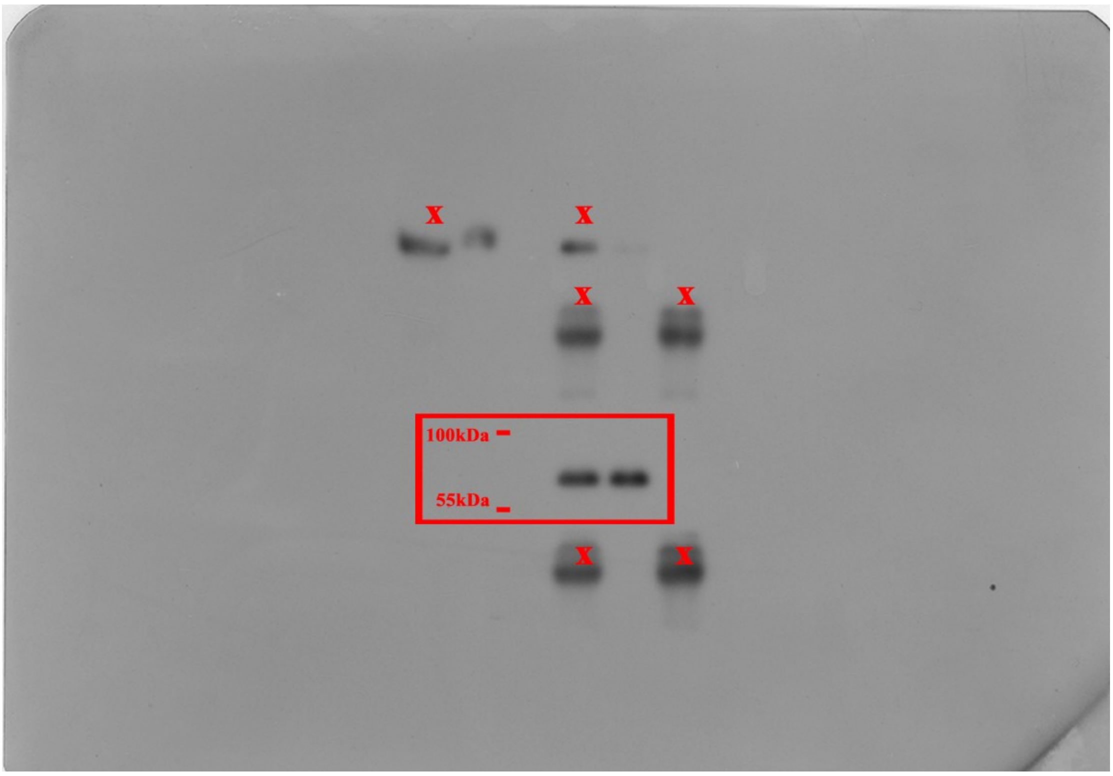

Figure 6.A-Gnb5 (Input, Hippocampus)

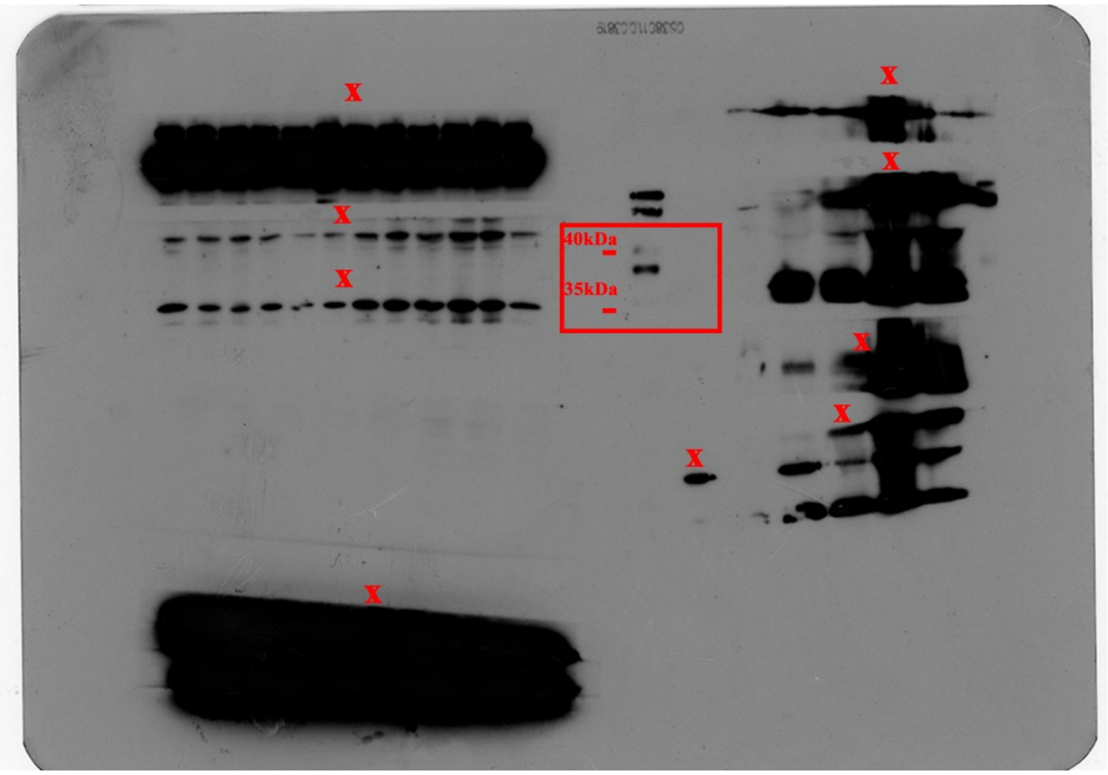

Figure 6.A- α-Tubulin (Input, Hippocampus)

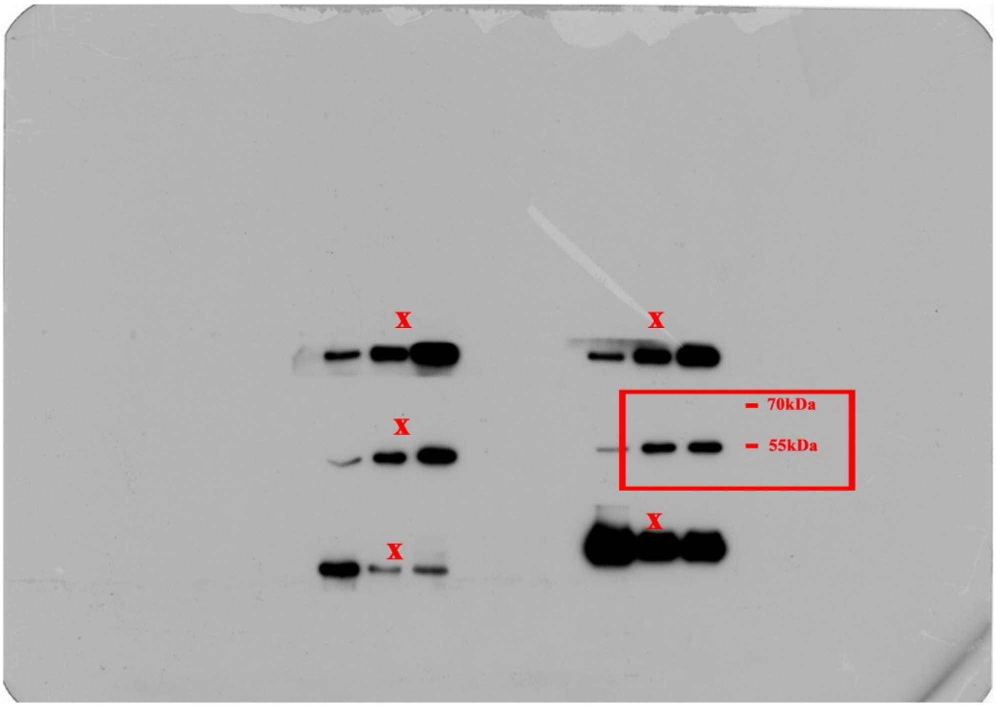

Figure 6.B- Myc-BACE1 (HEK293T cells)

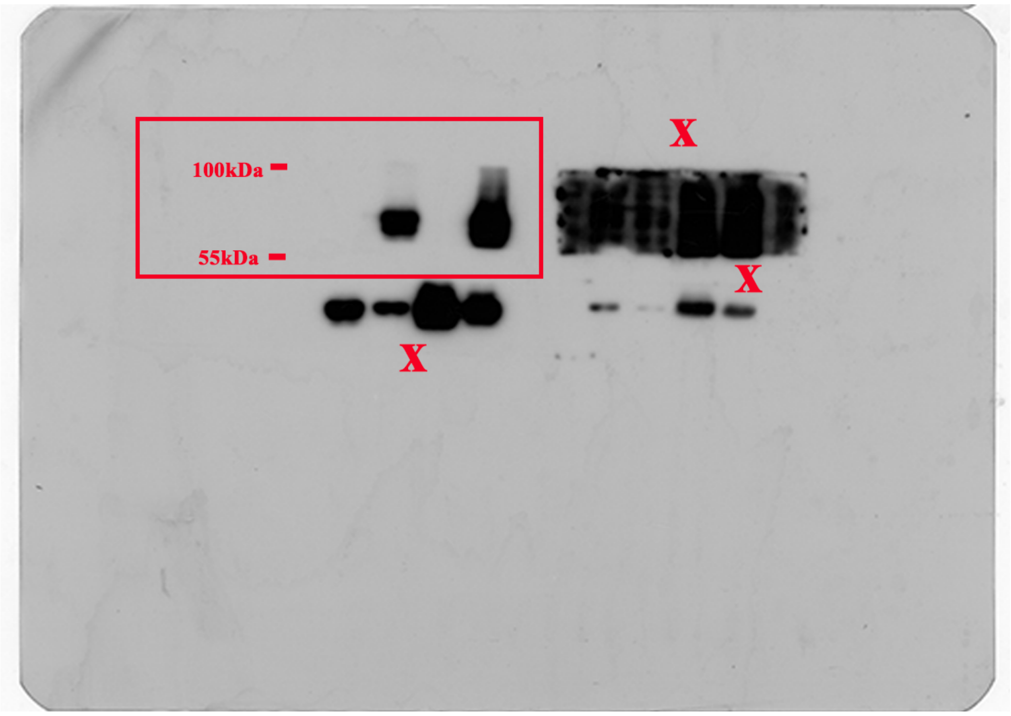

Figure 6.B- HA-Gnb5 (HEK293T cells)

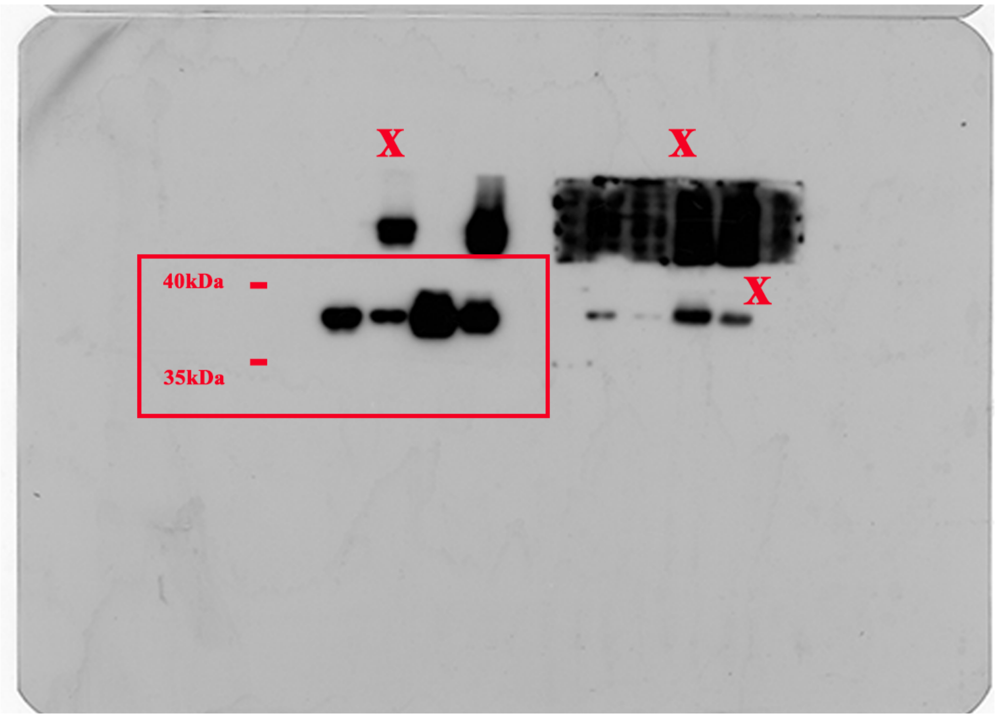

Figure 6.D-Myc (IP:HA, HEK293T cells)

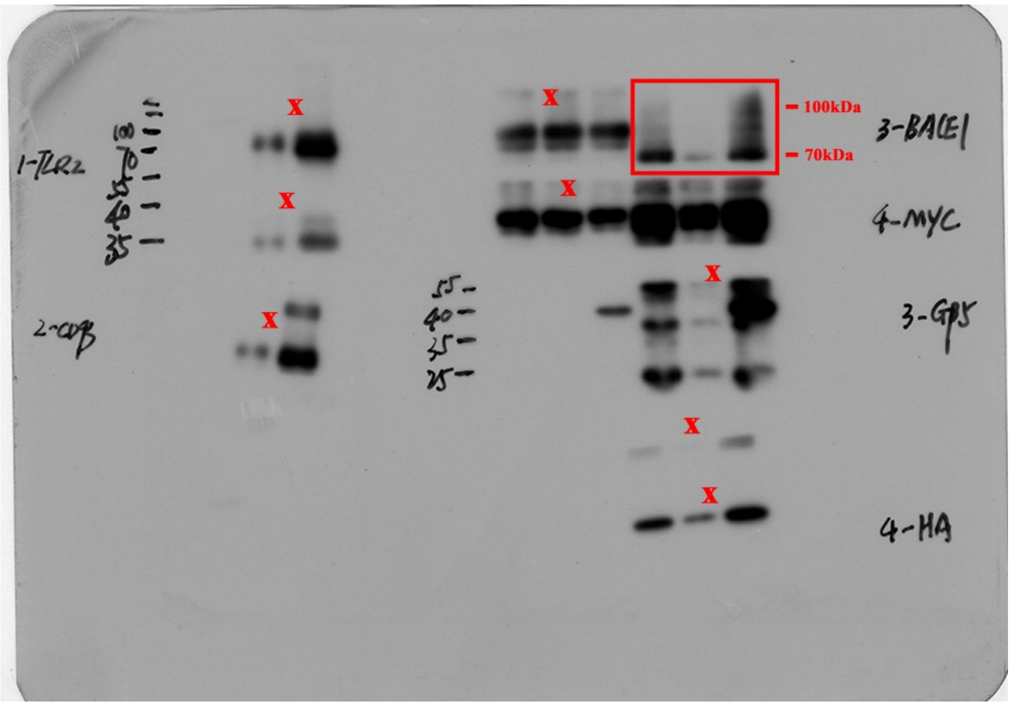

Figure 6.D-HA (IP:HA, HEK293T cells)

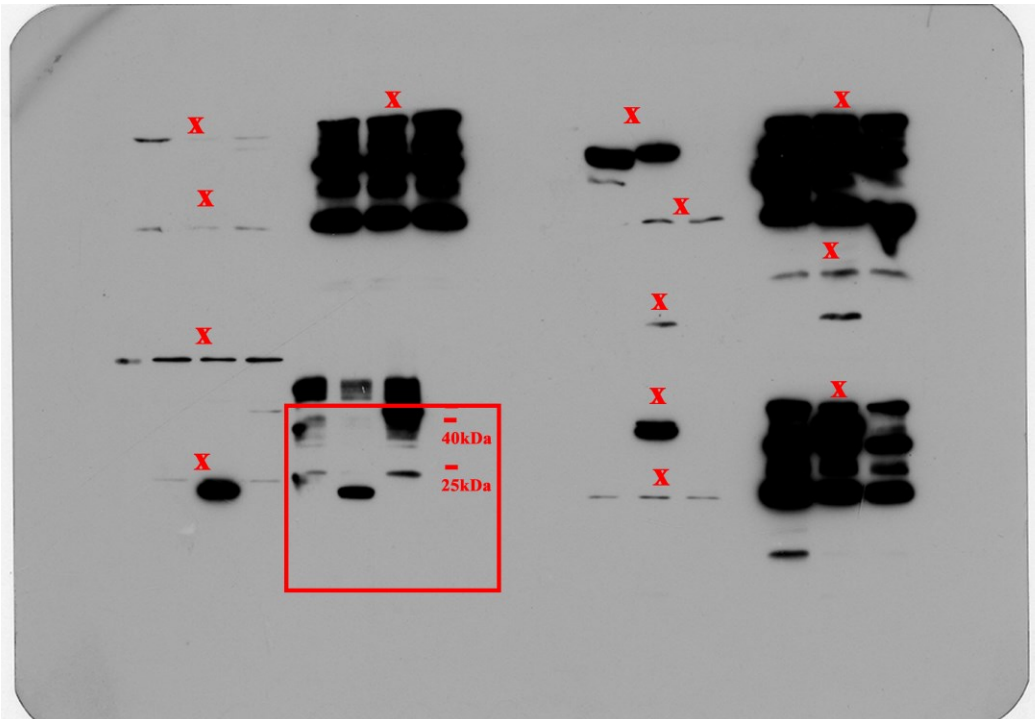

Figure 6.D-Myc (Input, HEK293T cells)

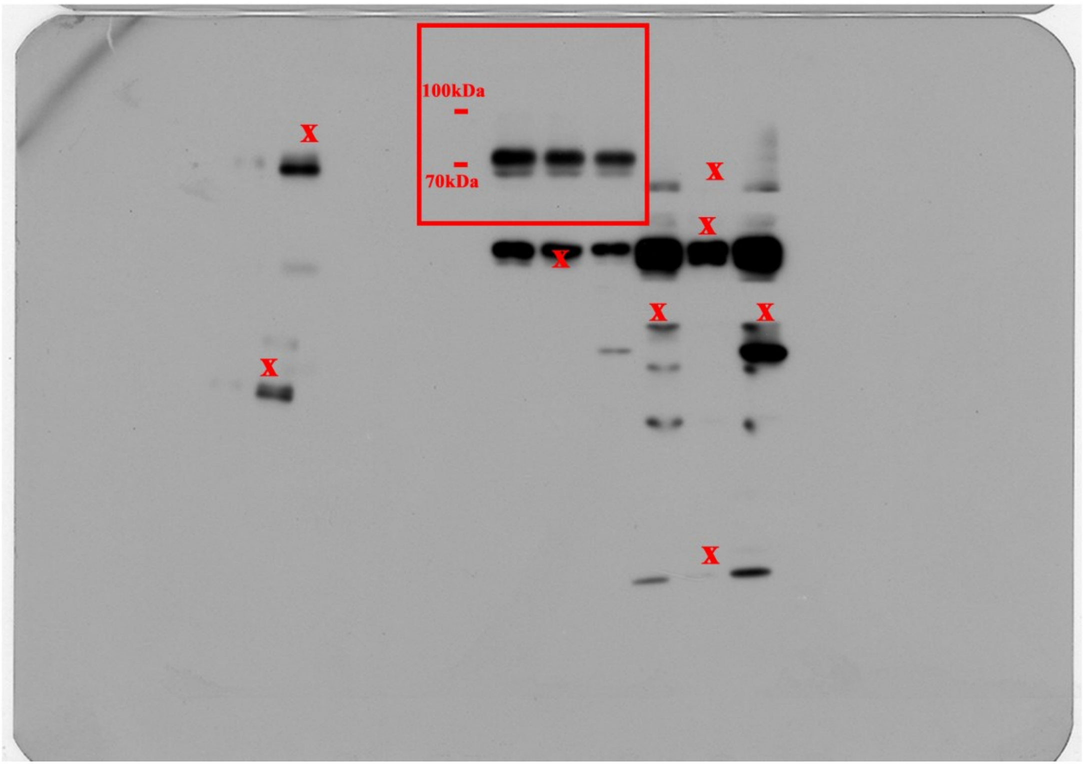

Figure 6.D-HA (Input, HEK293T cells)

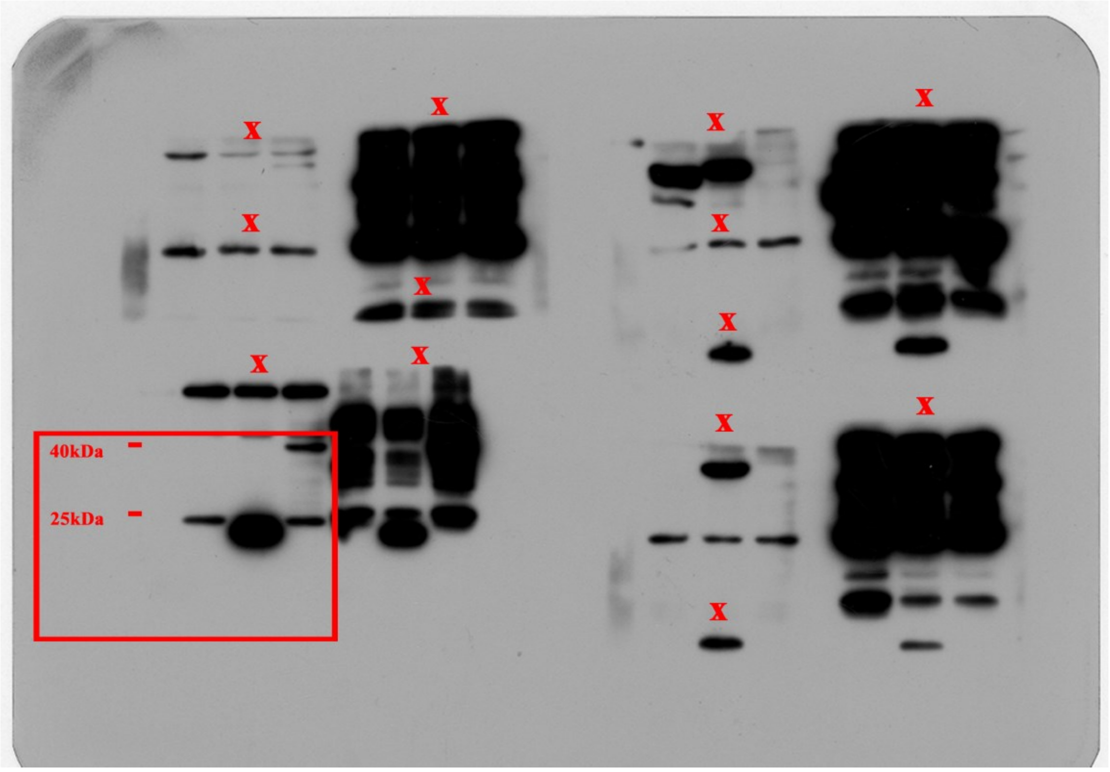

Figure 6.E-Myc (IP:HA, HEK293T cells)

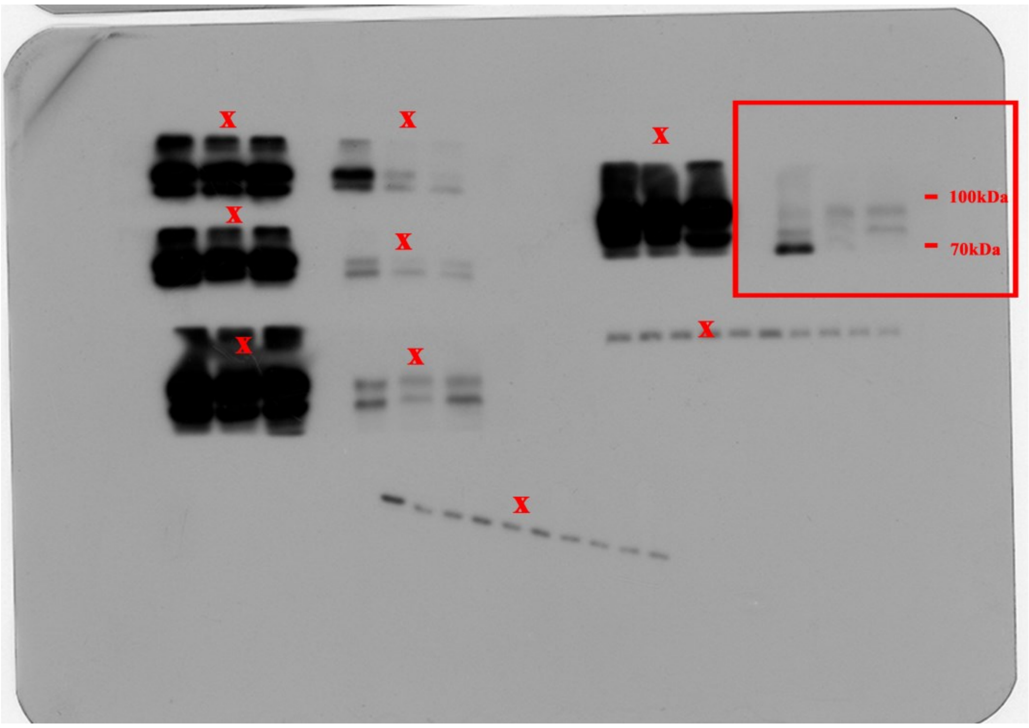

Figure 6.E-HA (IP:HA, HEK293T cells)

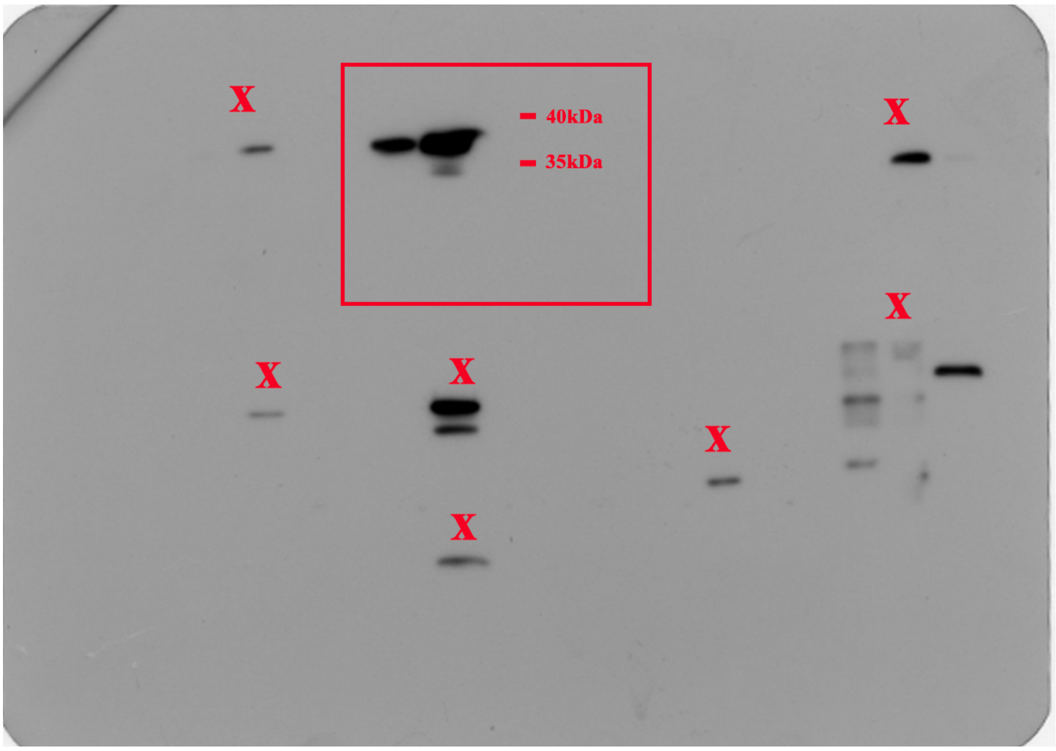

Figure 6.E-Myc (Input, HEK293T cells)

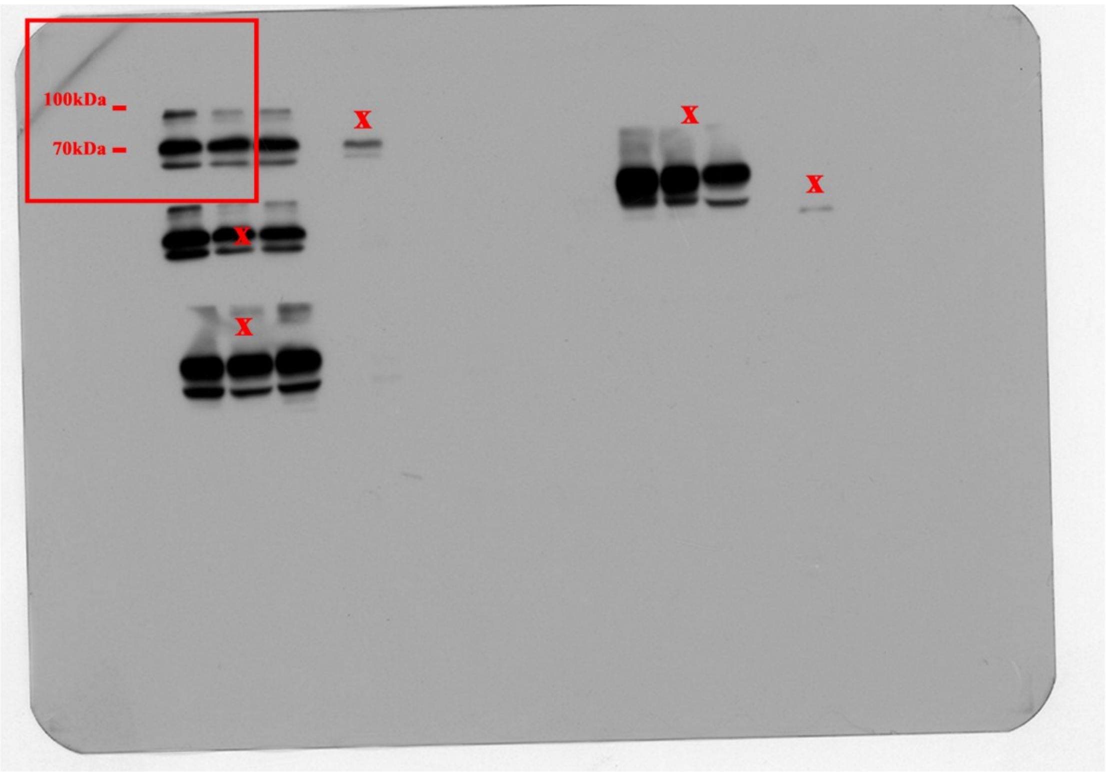

Figure 6.E-HA (Input, HEK293T cells)

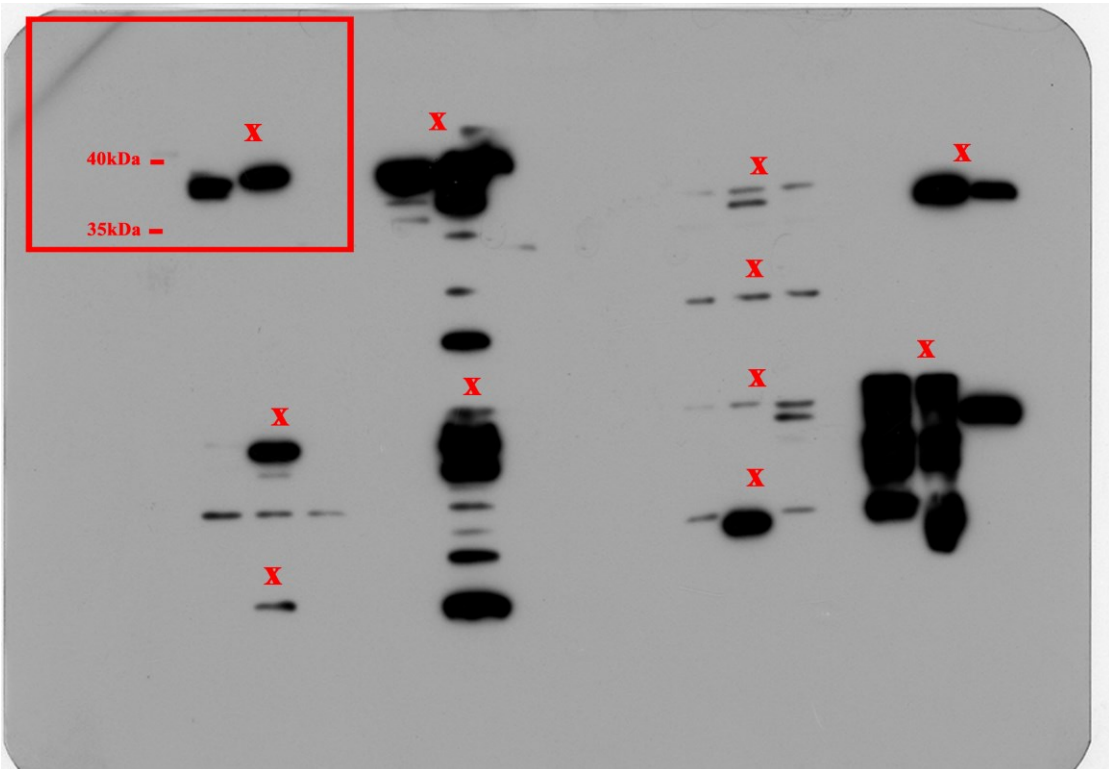

Figure 6.F-Myc (IP:HA, HEK293T cells)

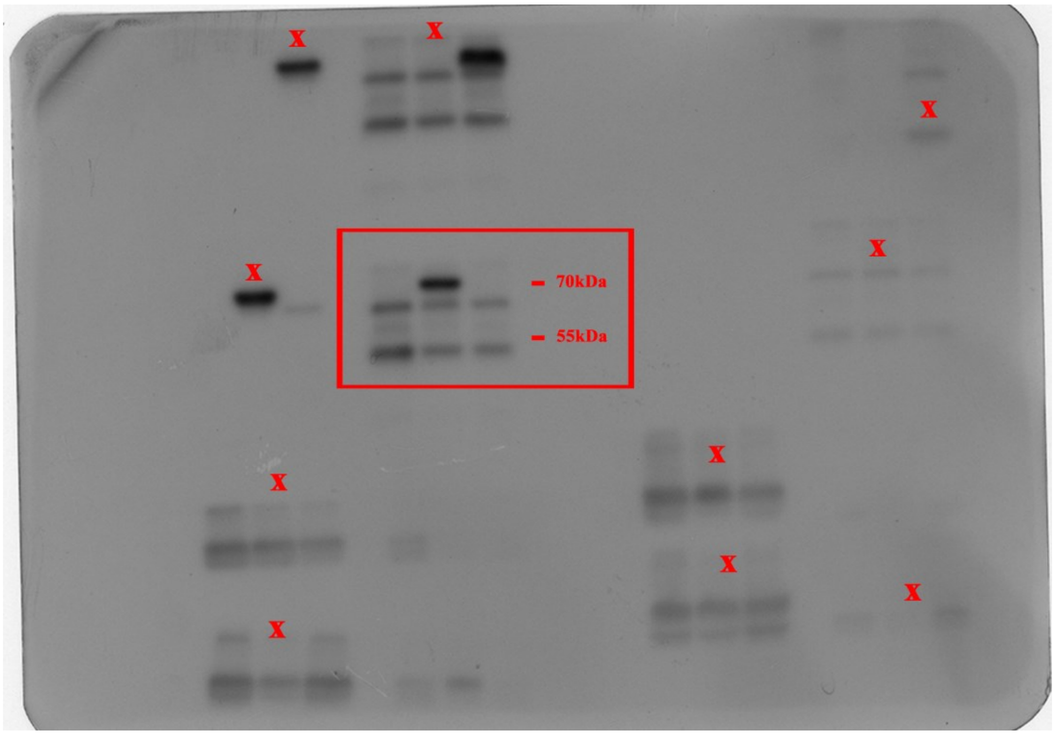

Figure 6.F-HA (IP:HA, HEK293T cells)

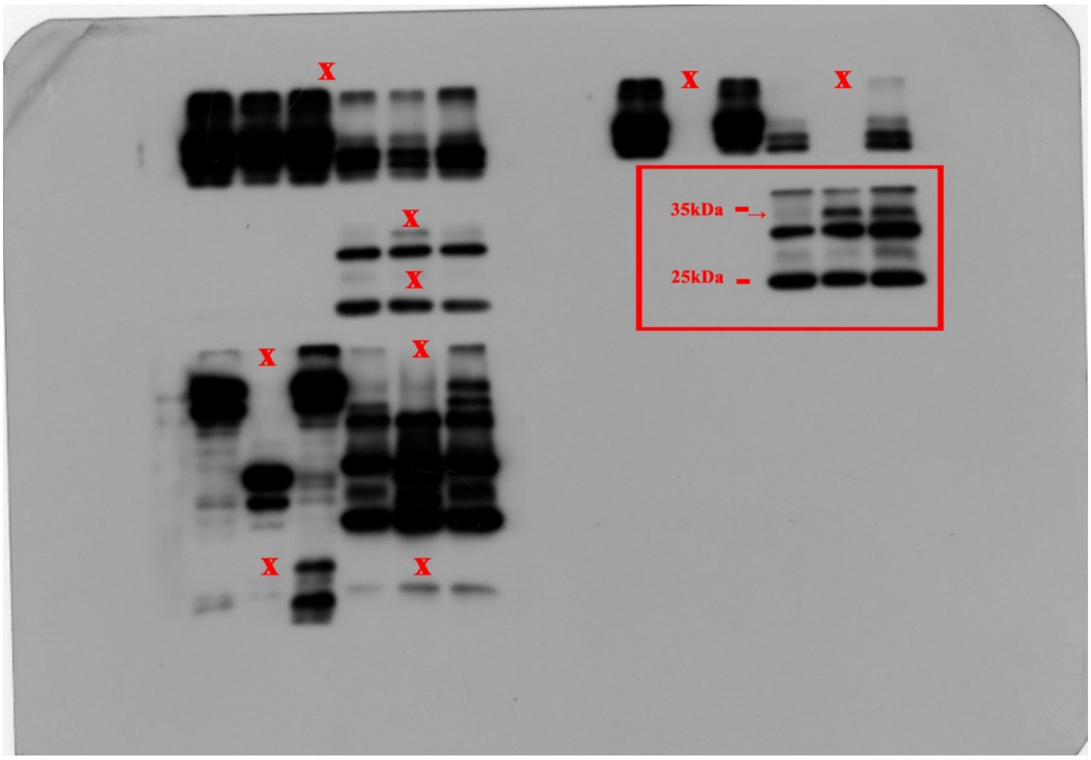

Figure 6.F-Myc (Input, HEK293T cells)

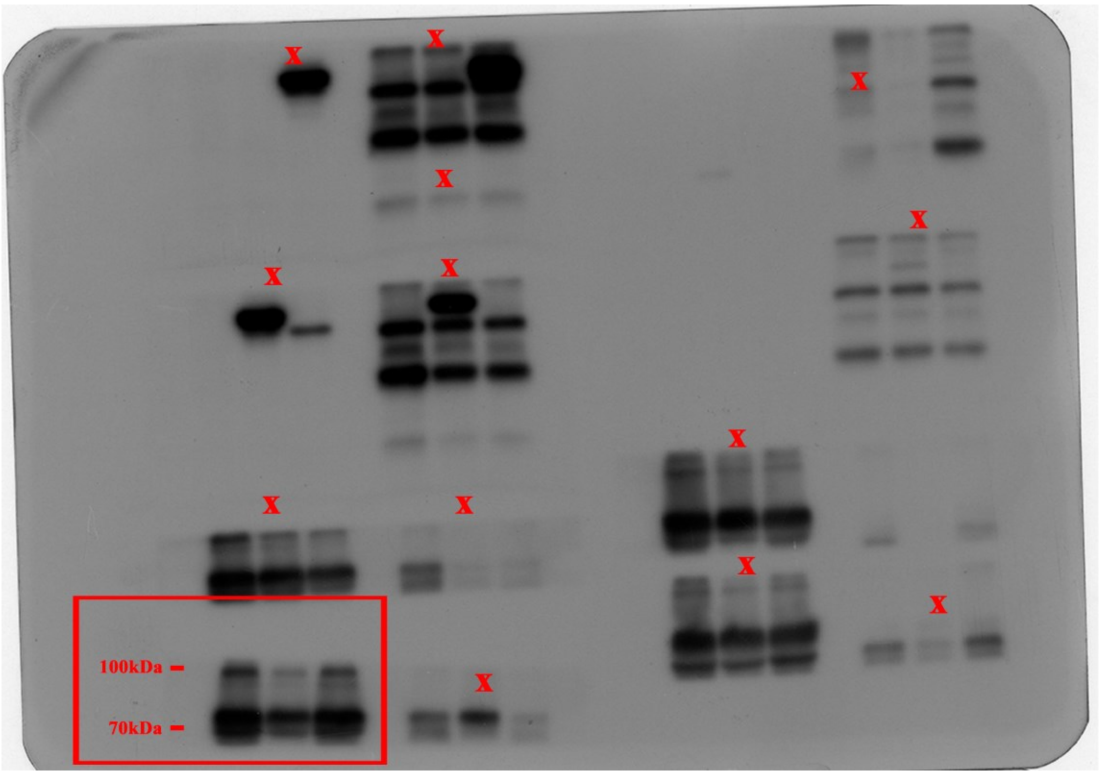

Figure 6.F-HA (Input, HEK293T cells)

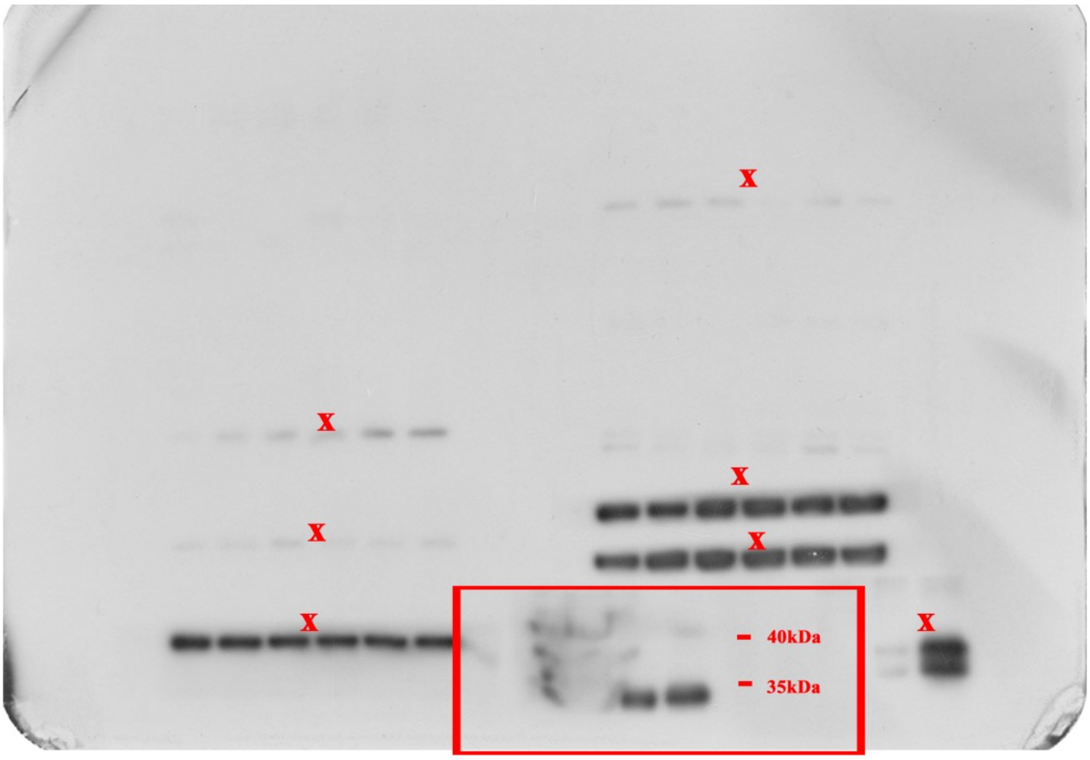

Figure 7

Figure 7.E-APP (Hippocampus, 10 months)

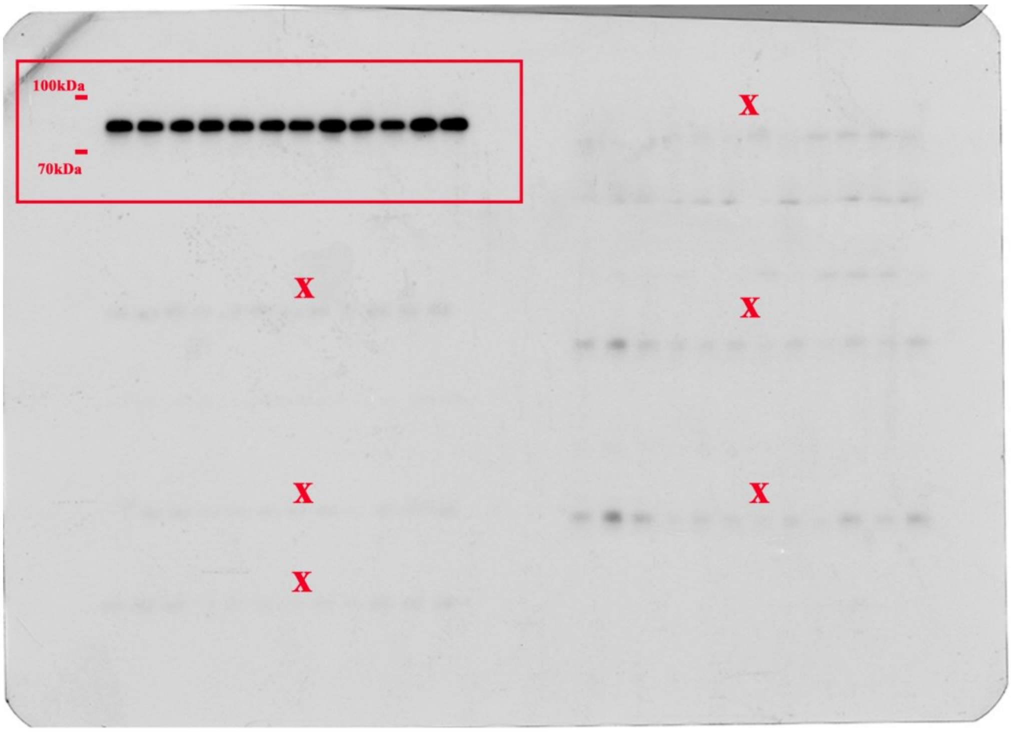

Figure 7.E-Nicastrin (Hippocampus, 10 months)

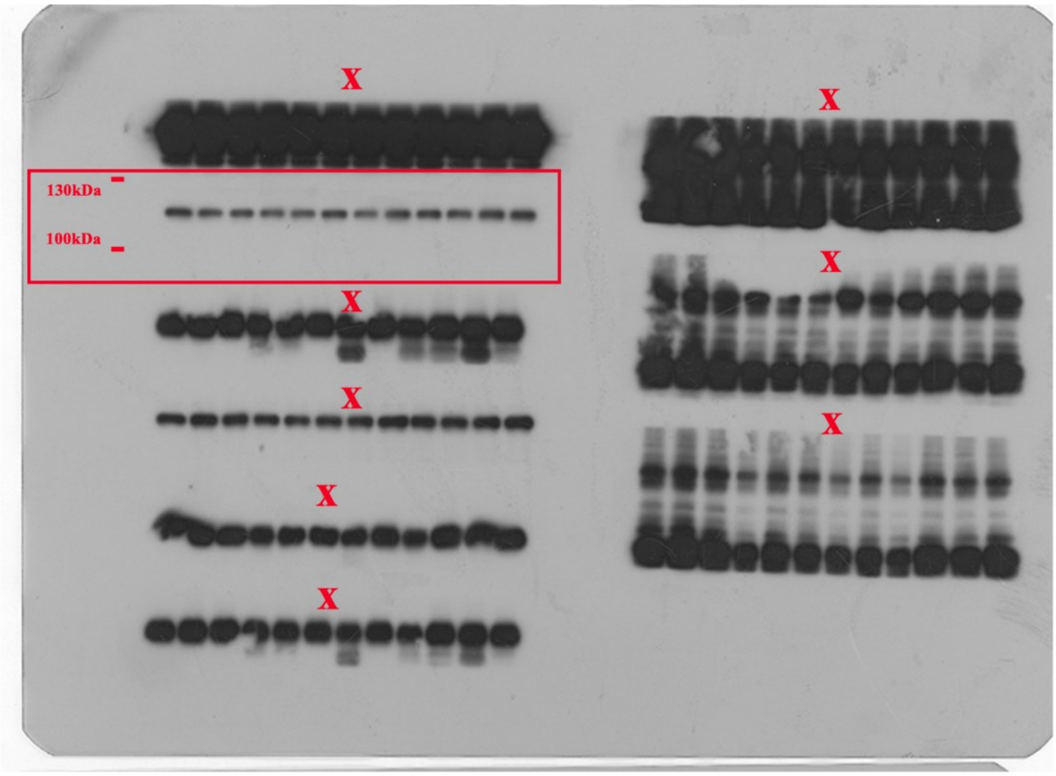

Figure 7.E-BACE1 (Hippocampus, 10 months)

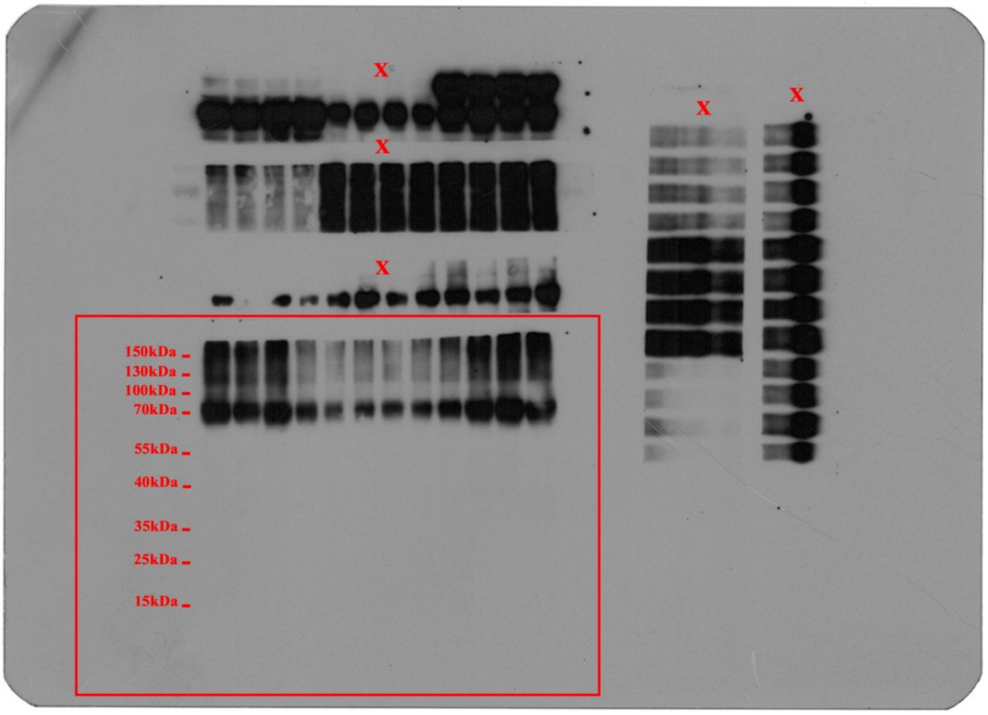

Figure 7.E-β-CTF (Hippocampus, 10 months)

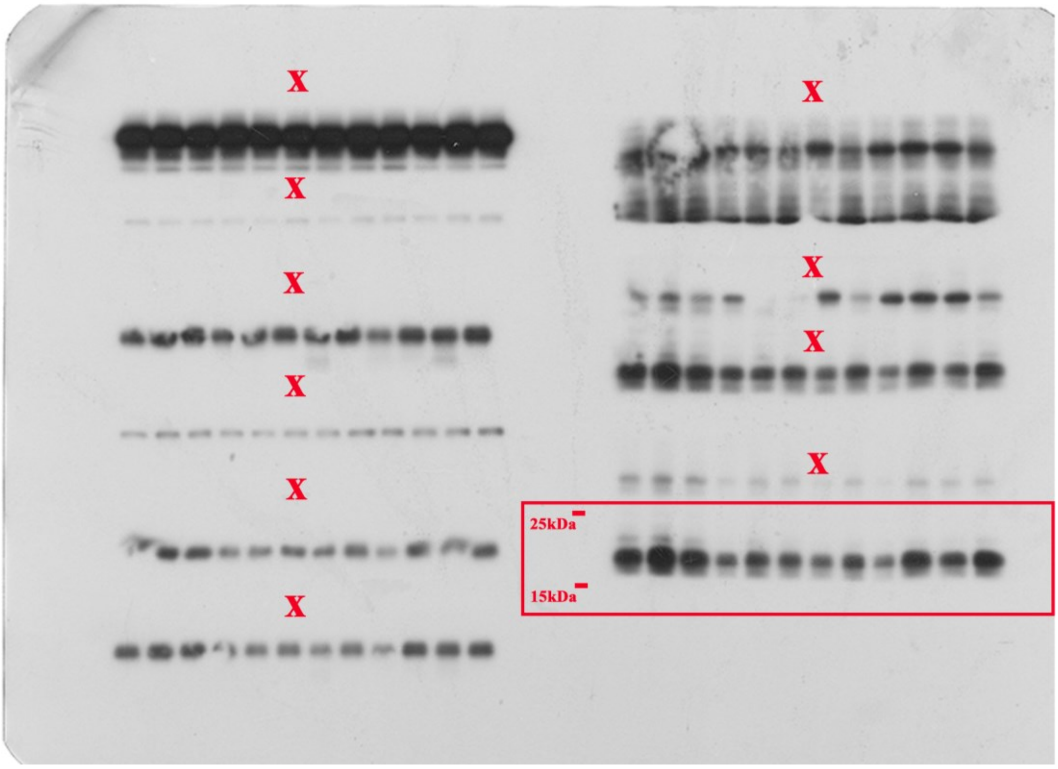

Figure 7.E-GFP (Hippocampus, 10 months)

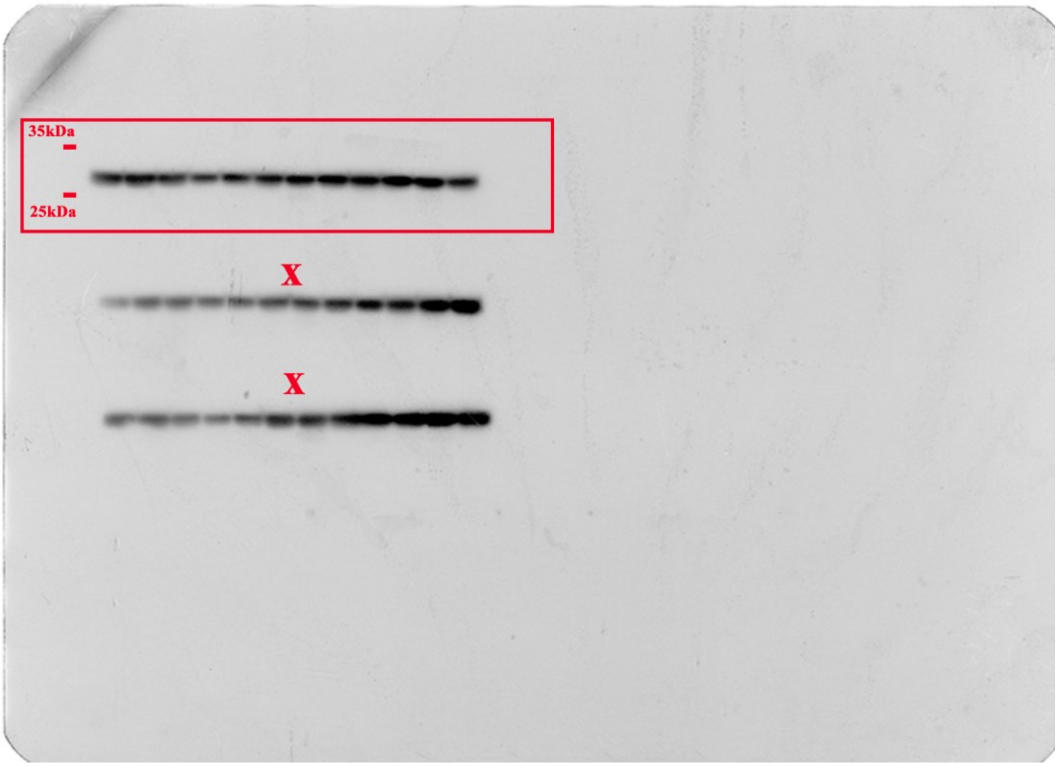

Figure 7.E-α-Tubulin (Hippocampus, 10 months)

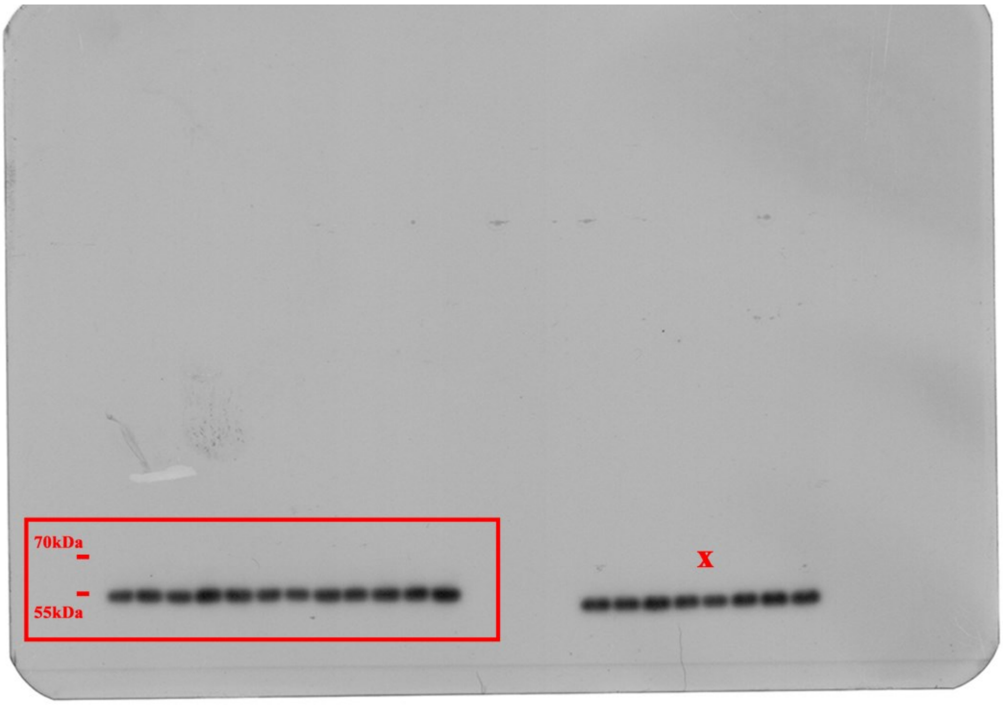

Figure S1

Figure S1.G-Gnb5 (Cortex, 3 months)

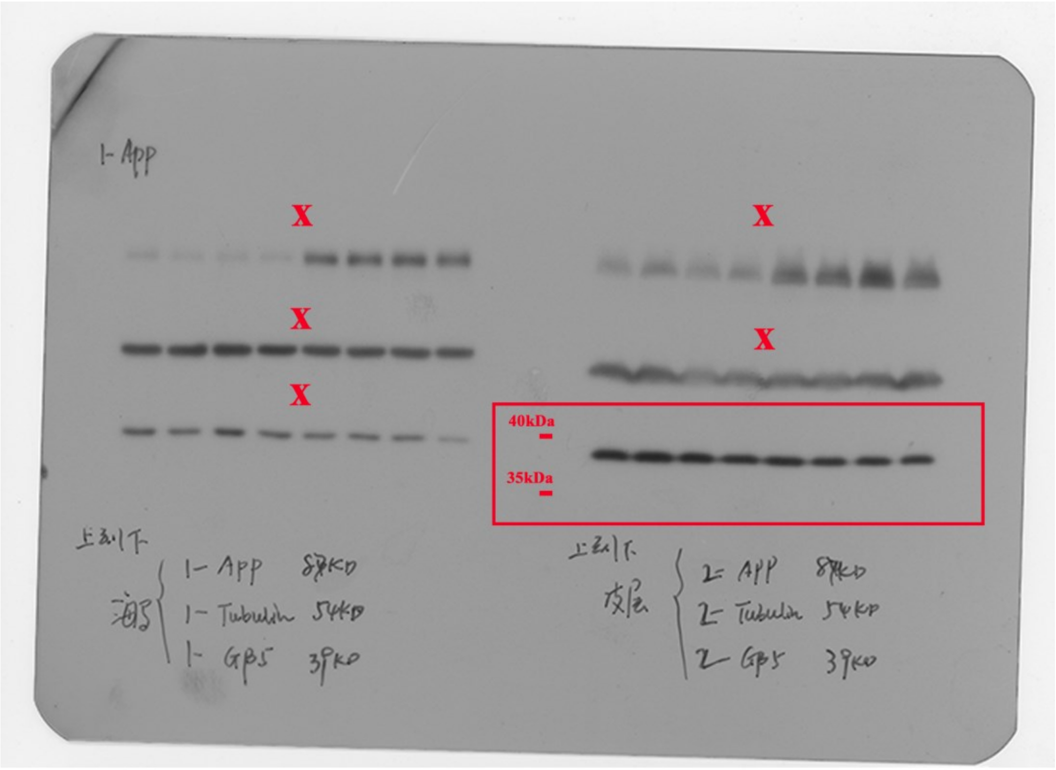

Figure S1.G-APP (Cortex, 3 months)

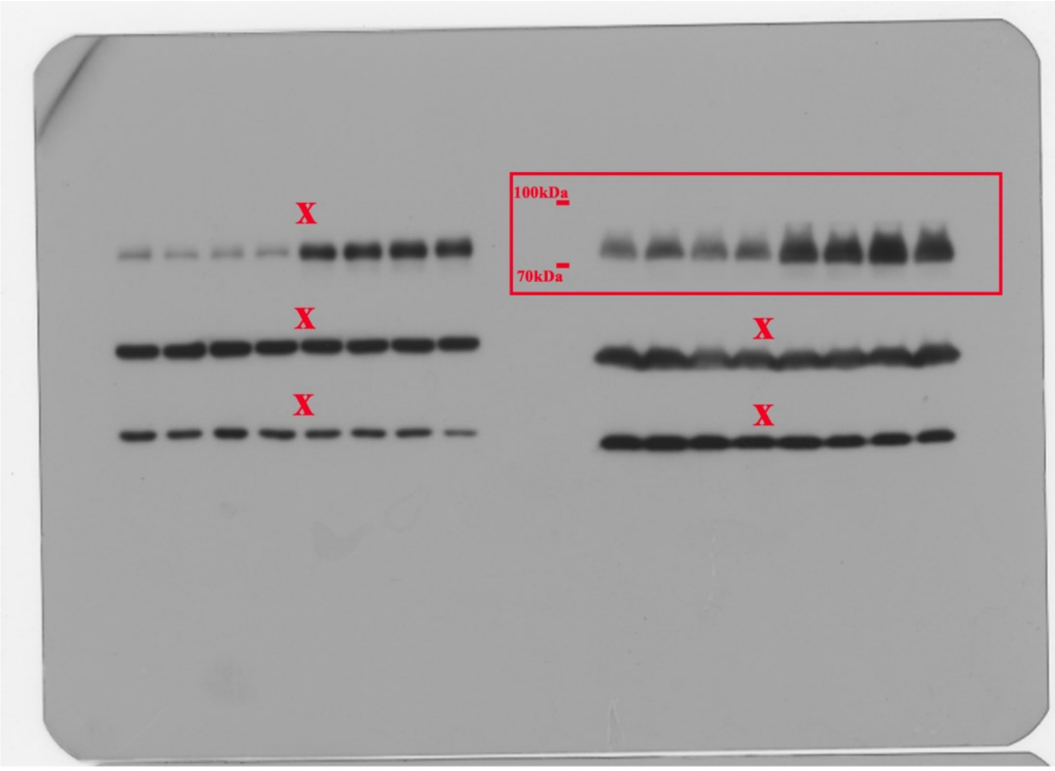

Figure S1.G-α-Tubulin (Cortex, 3 months)

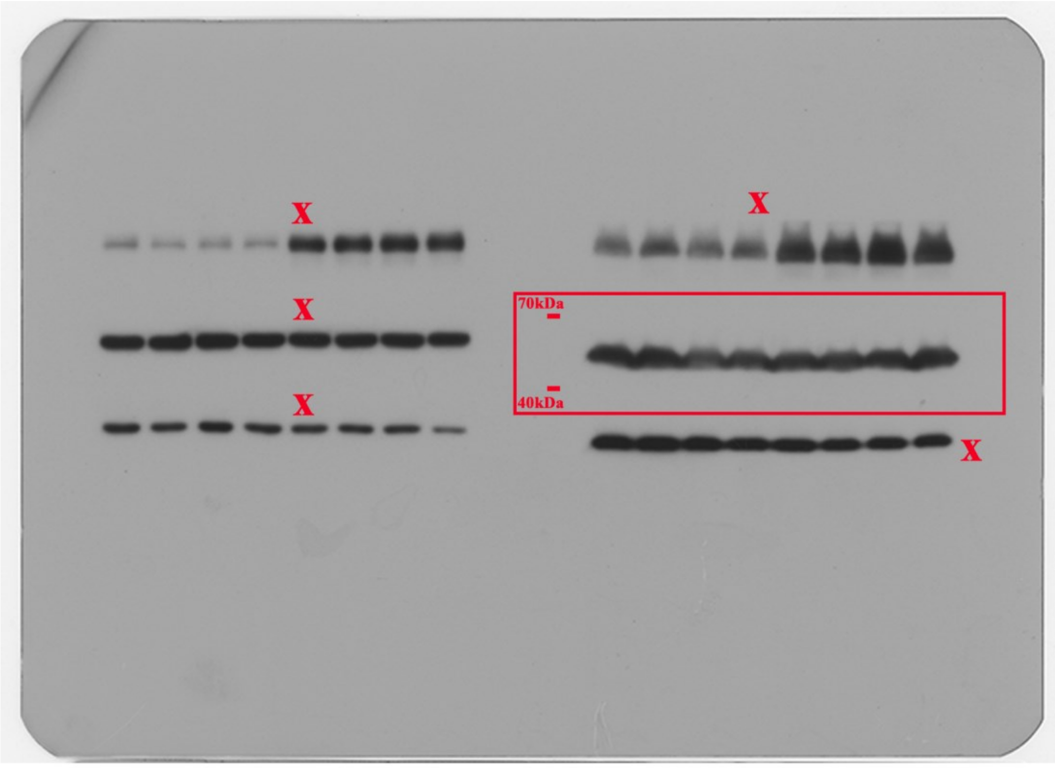

Figure S1.I-Gnb5 (Cortex, 6 months)

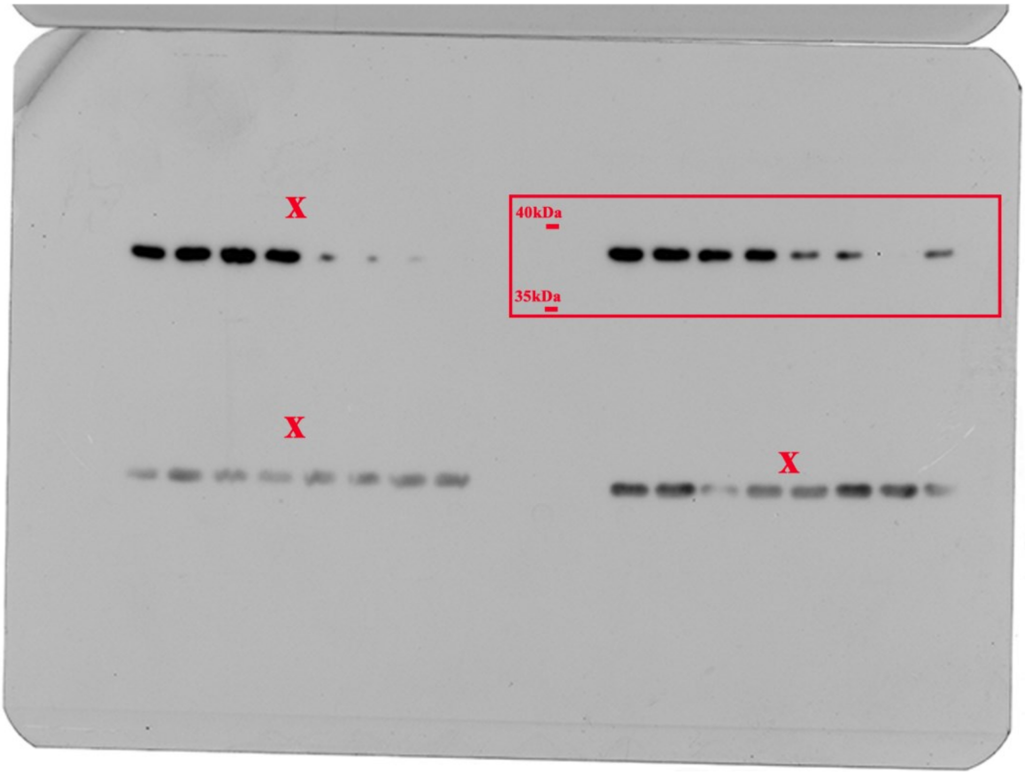

Figure S1.I-APP (Cortex, 6 months)

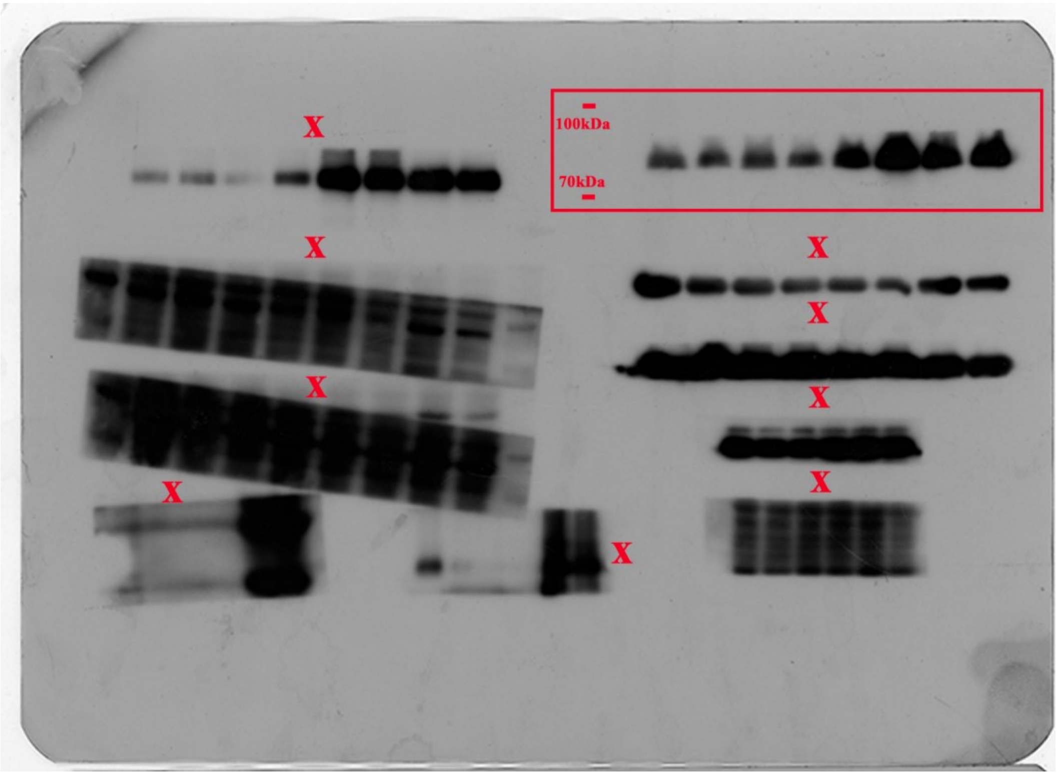

Figure S1.I-α-Tubulin (Cortex, 6 months)

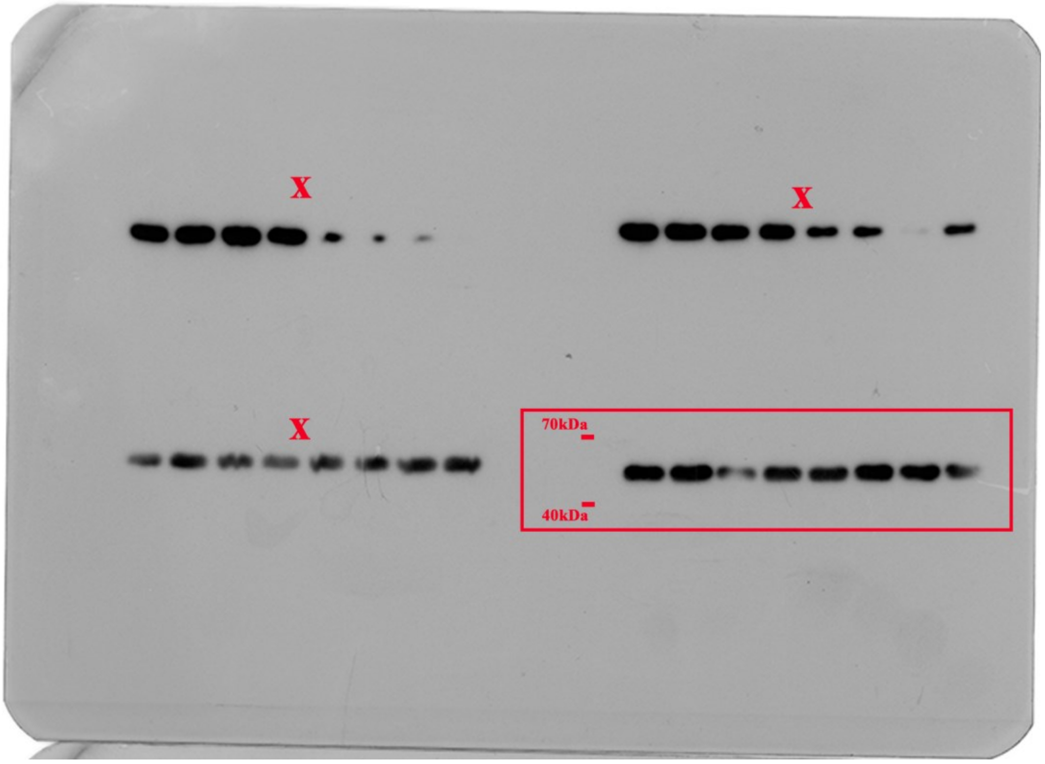

Figure S1.K-Gnb5 (Cultured primary neurons)

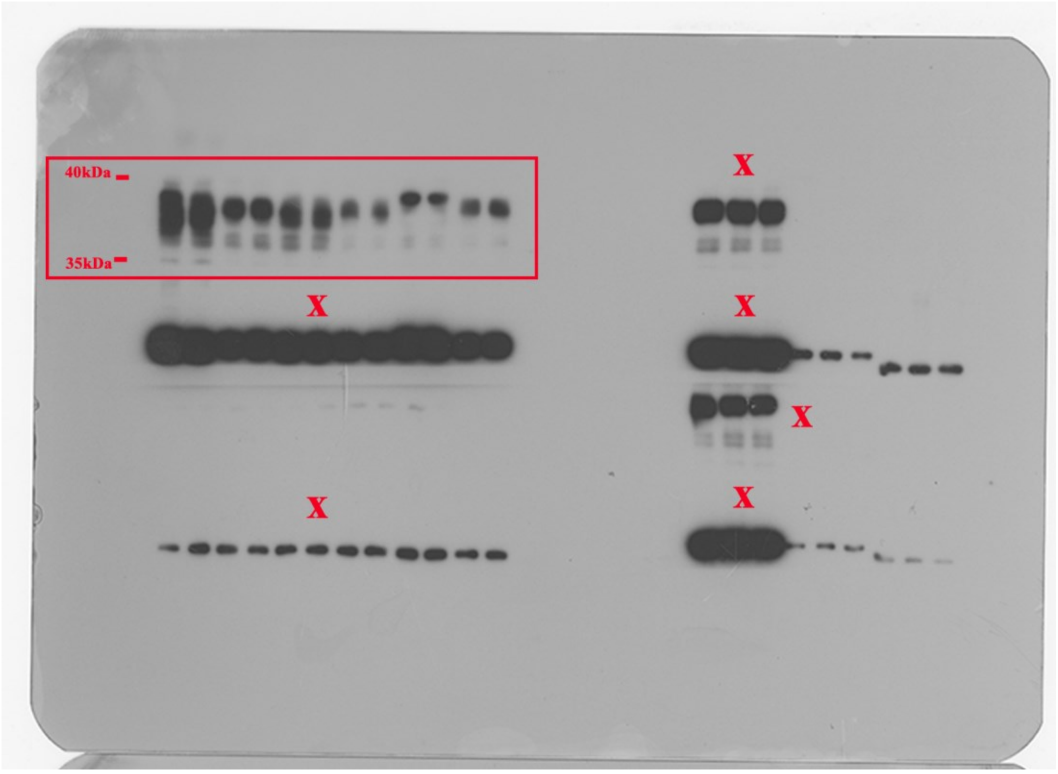

Figure S1.K-α-Tubulin (Cultured primary neurons)

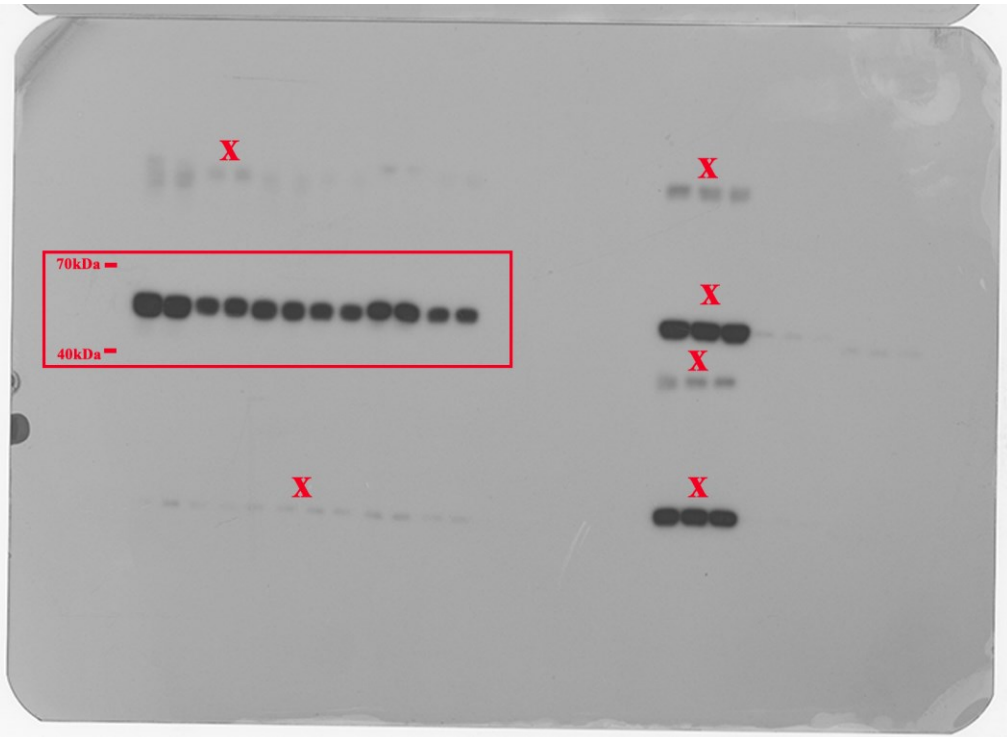

Figure S2

Figure S2.A-Gnb5 (Tissue-wide analysis)

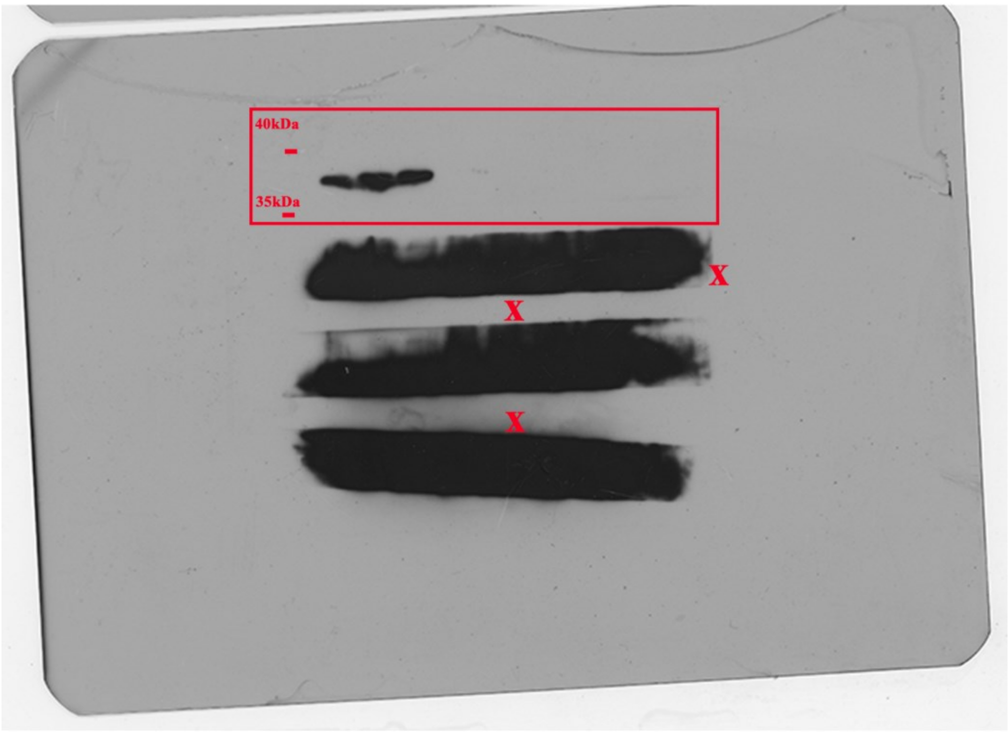

Figure S2.A-α-Tubulin (Tissue-wide analysis)

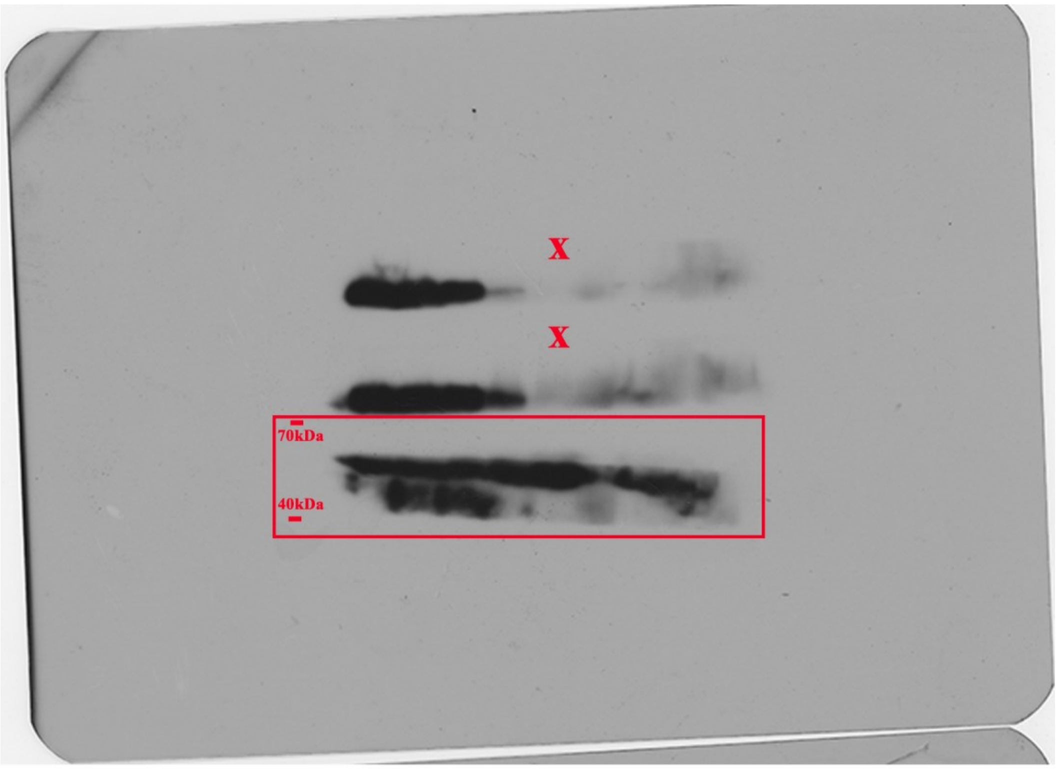

Figure S2.B-Gnb5 (Primary cultured neural cells)

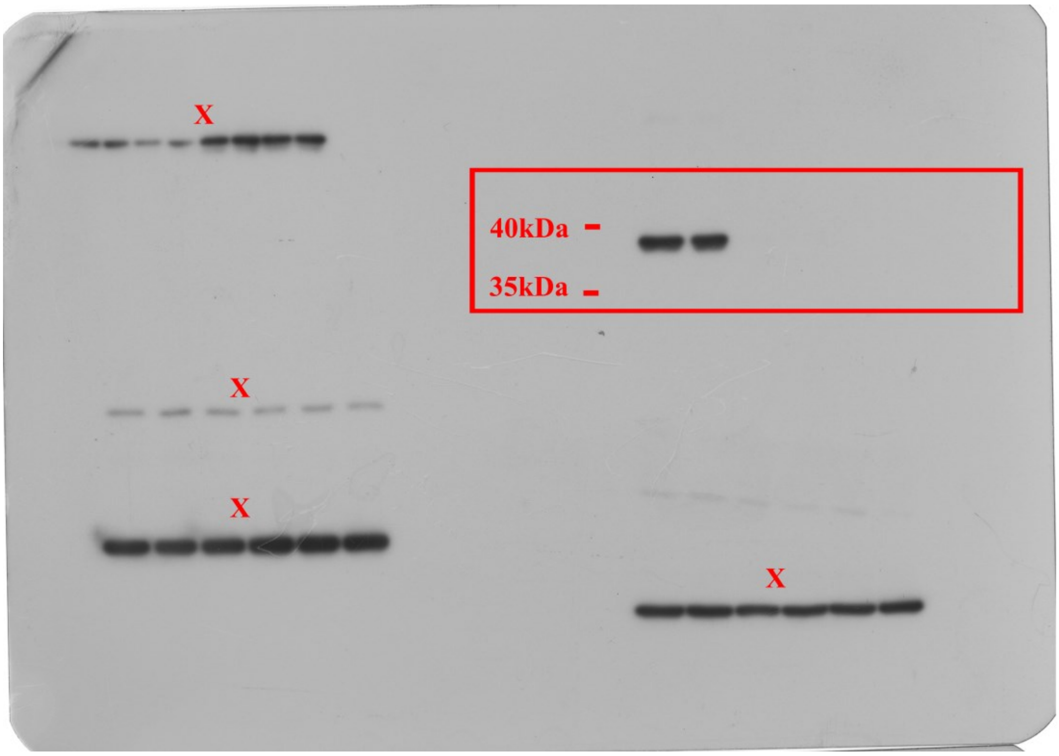

Figure S2.B-Tuj1 (Primary cultured neural cells)

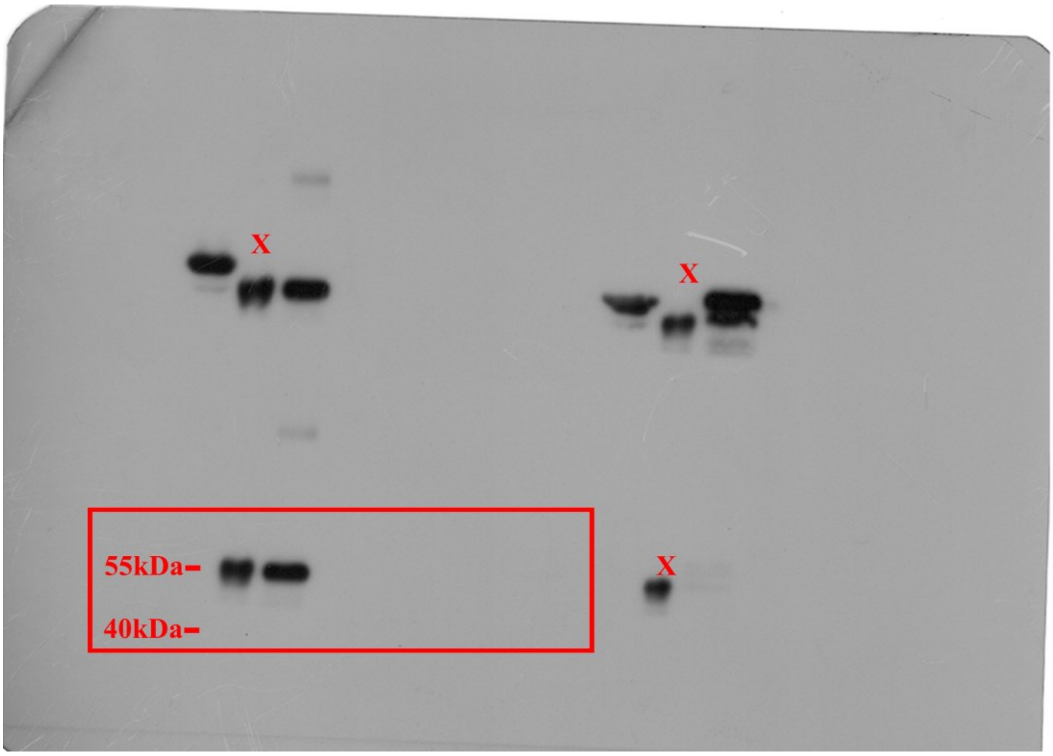

Figure S2.B-Gfap (Primary cultured neural cells)

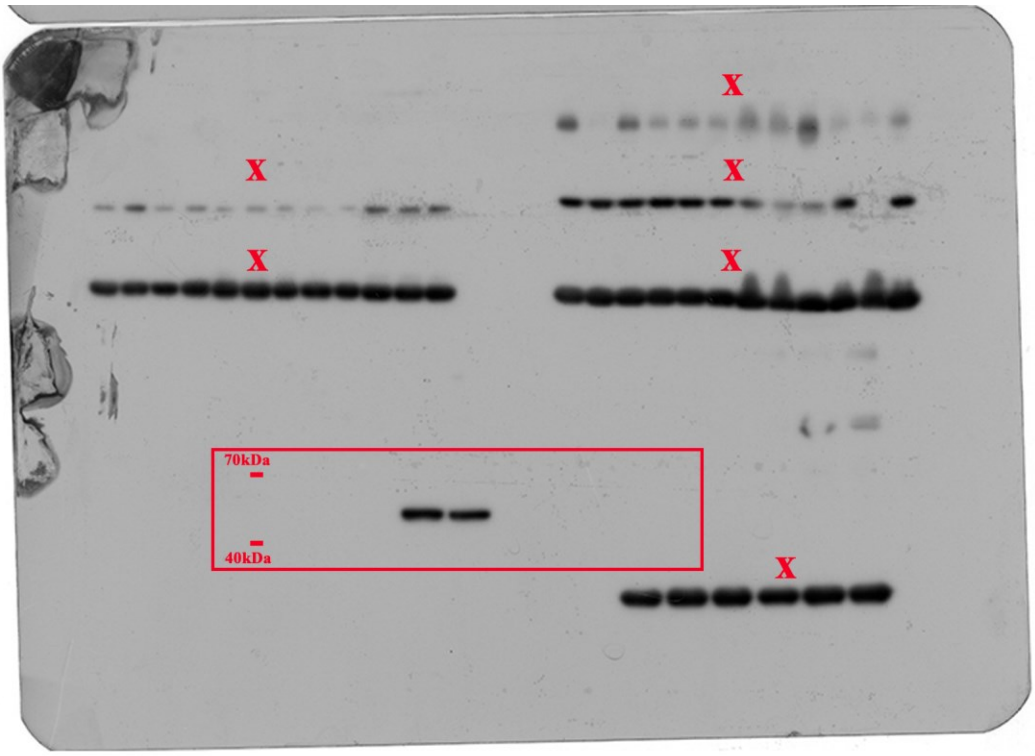

Figure S2.B-Iba1 (Primary cultured neural cells)

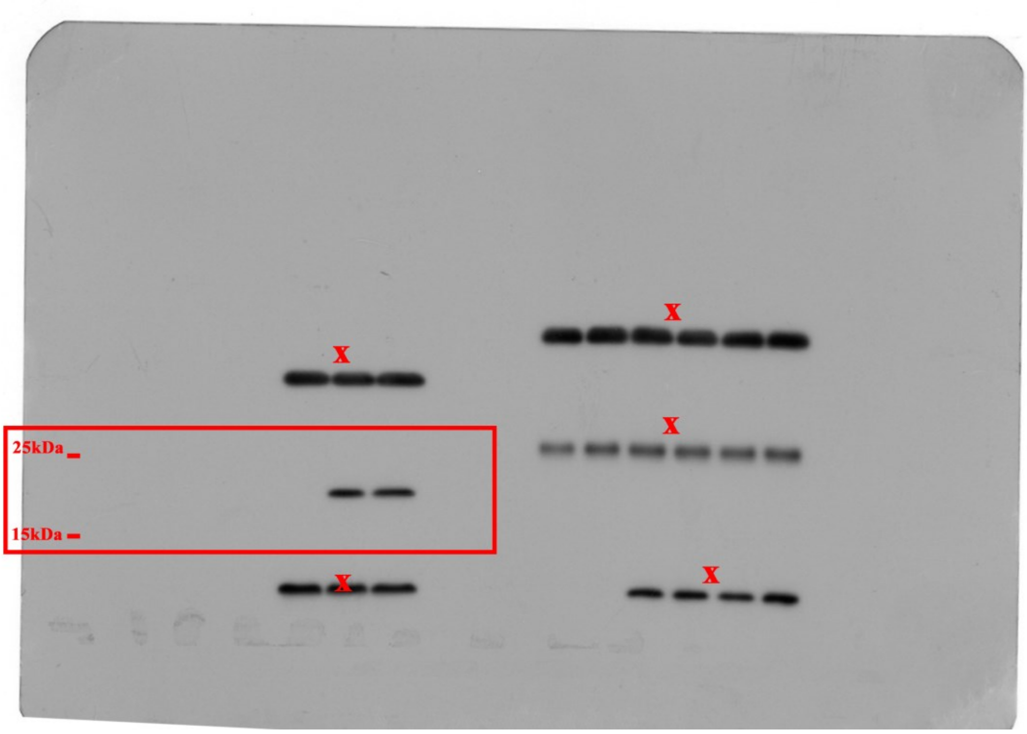

Figure S2.B-α-Tubulin (Primary cultured neural cells)

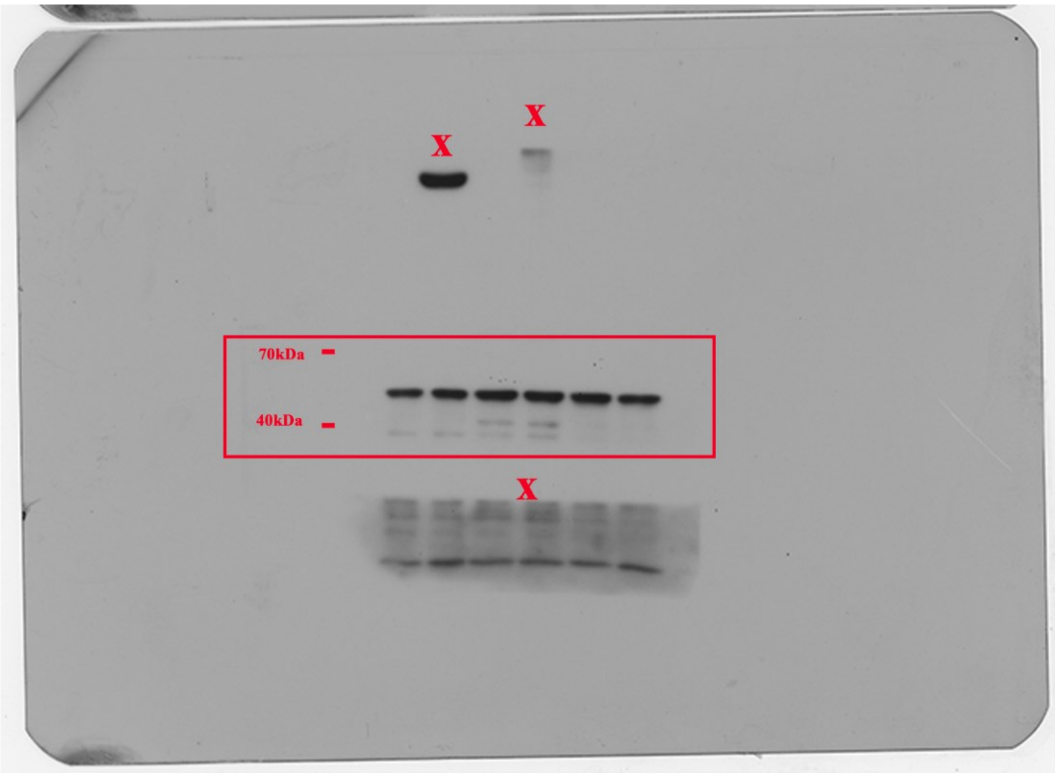

Figure S2.H-5’-arm (Genotyping)

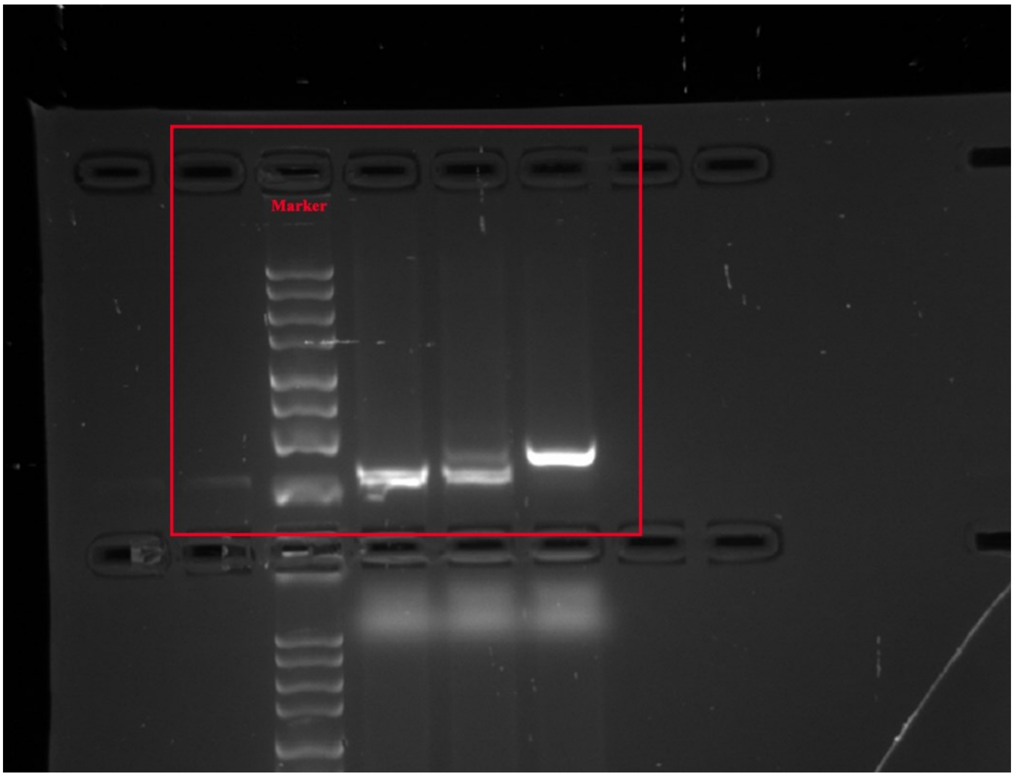

Figure S2.H-3’-arm (Genotyping)

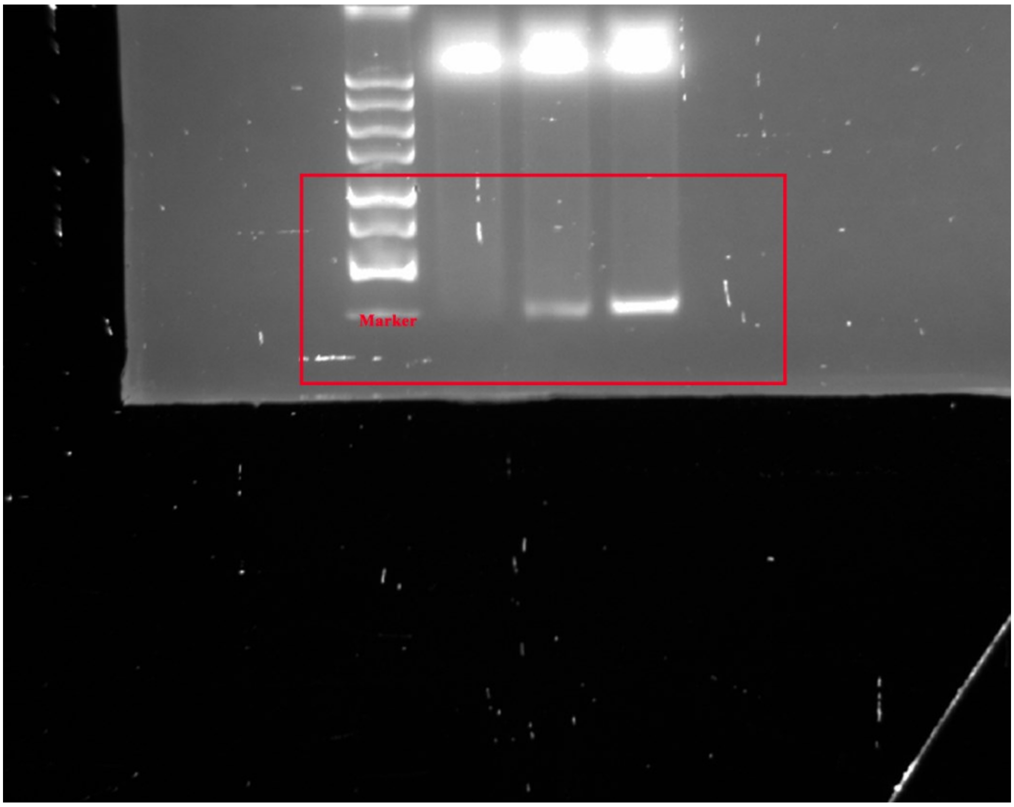

Figure S6

Figure S6.A-Rgs7 ((HEK293T cells))

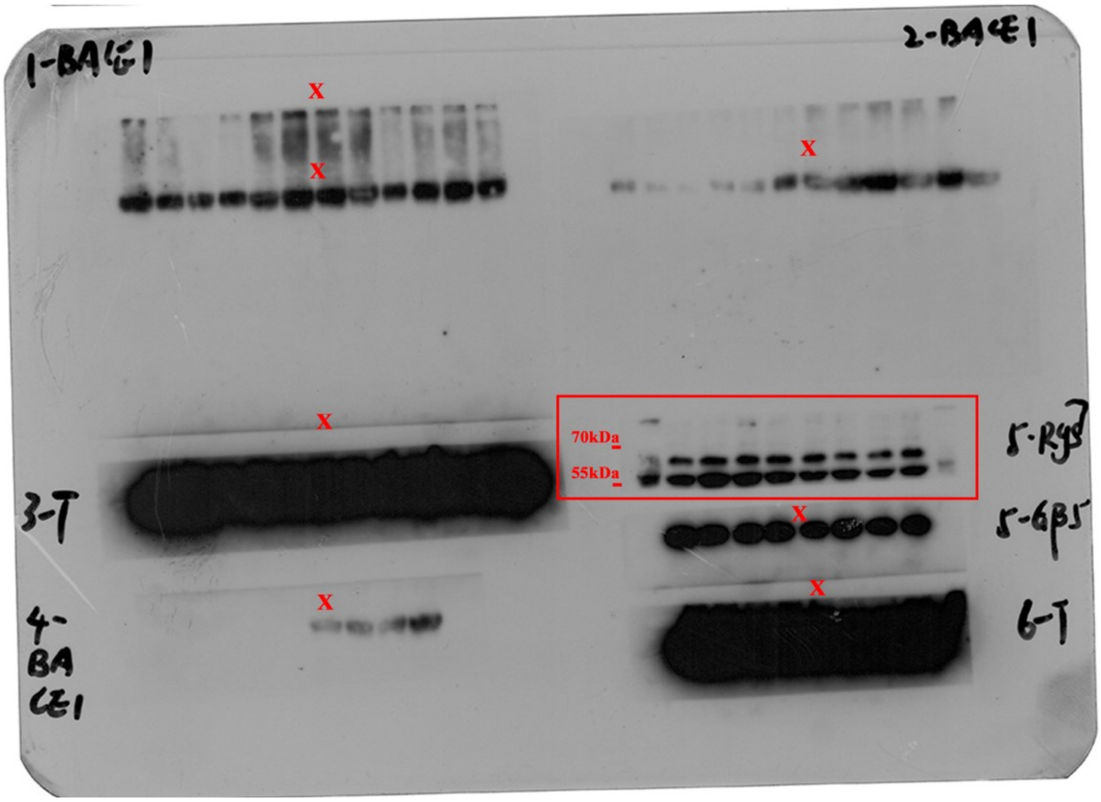

Figure S6.A-Akt (HEK293T cells)

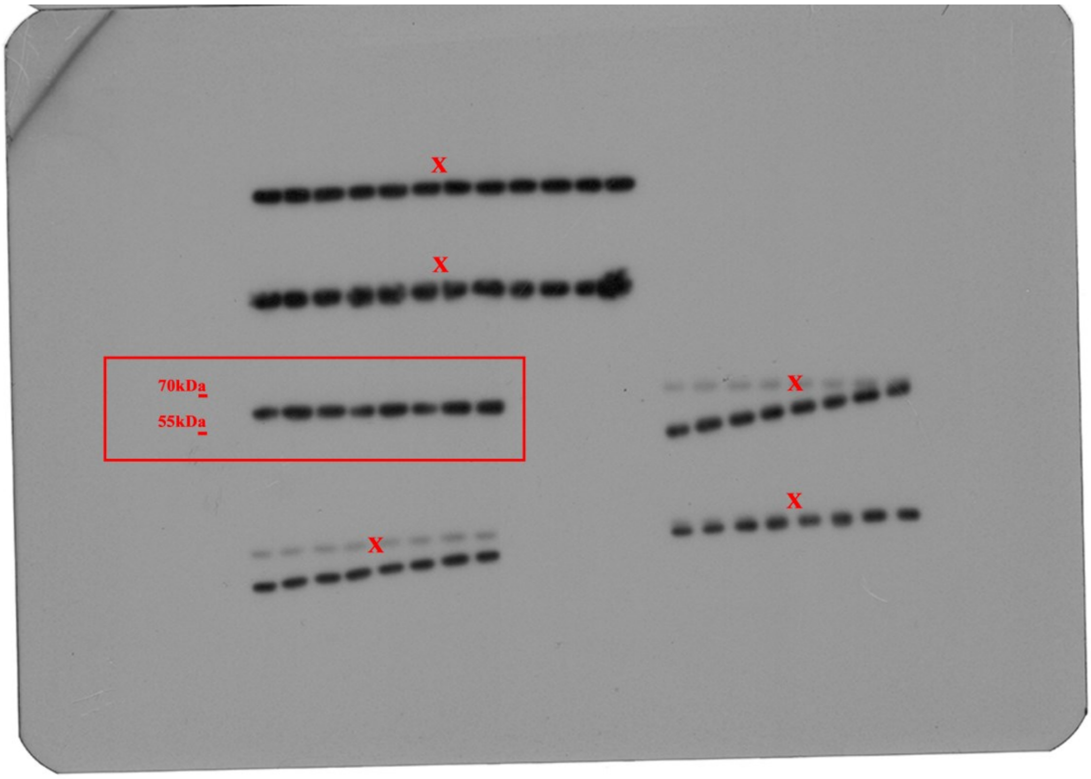

Figure S6.A-p-Akt (HEK293T cells)

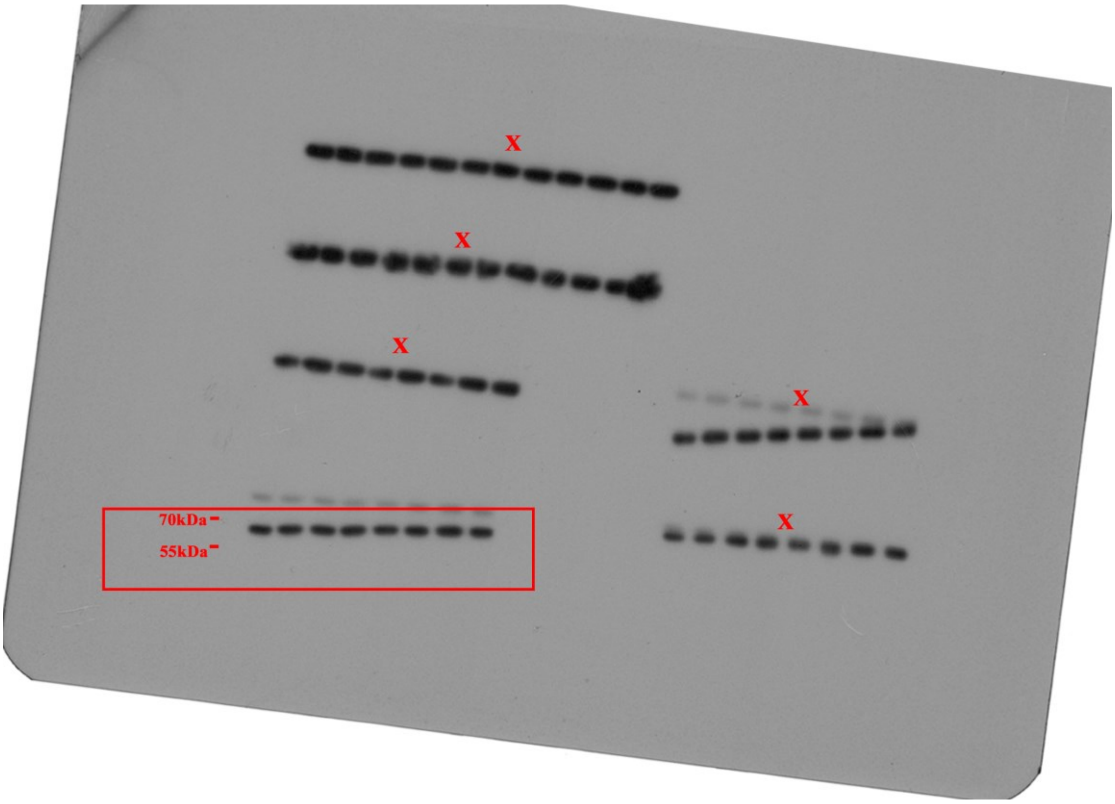

Figure S6.A-α-Tubulin (HEK293T cells)

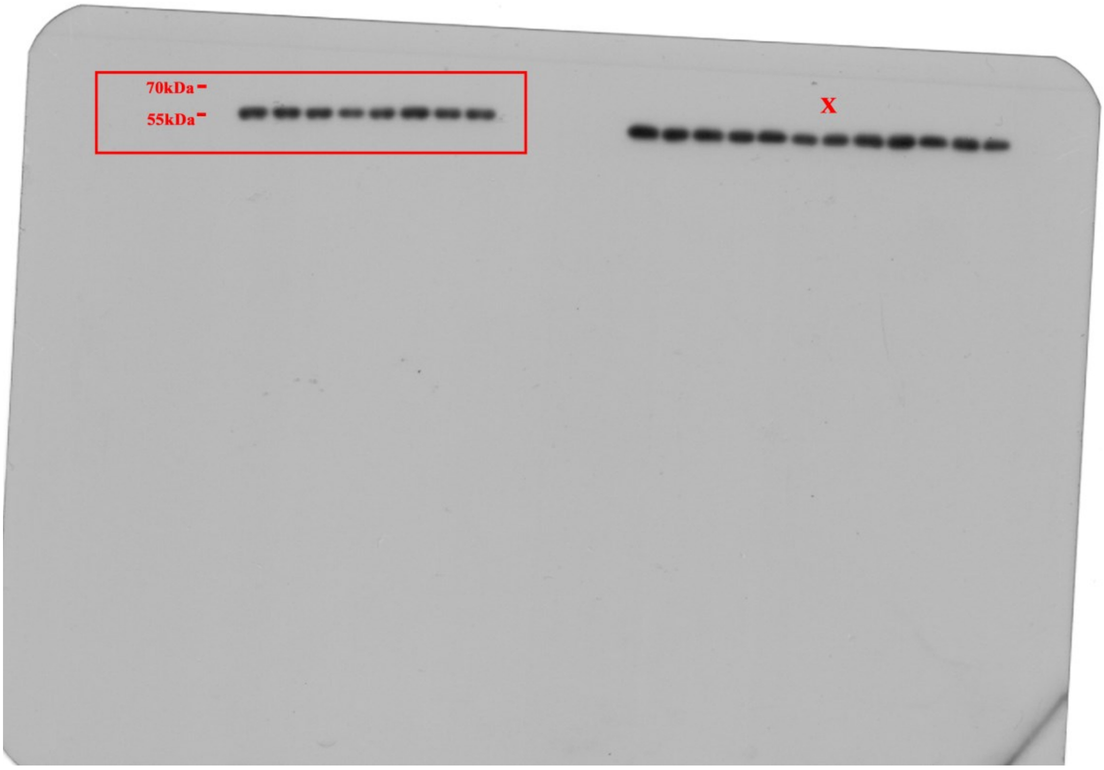

Figure S6.D-BACE1 (HEK293T cells)

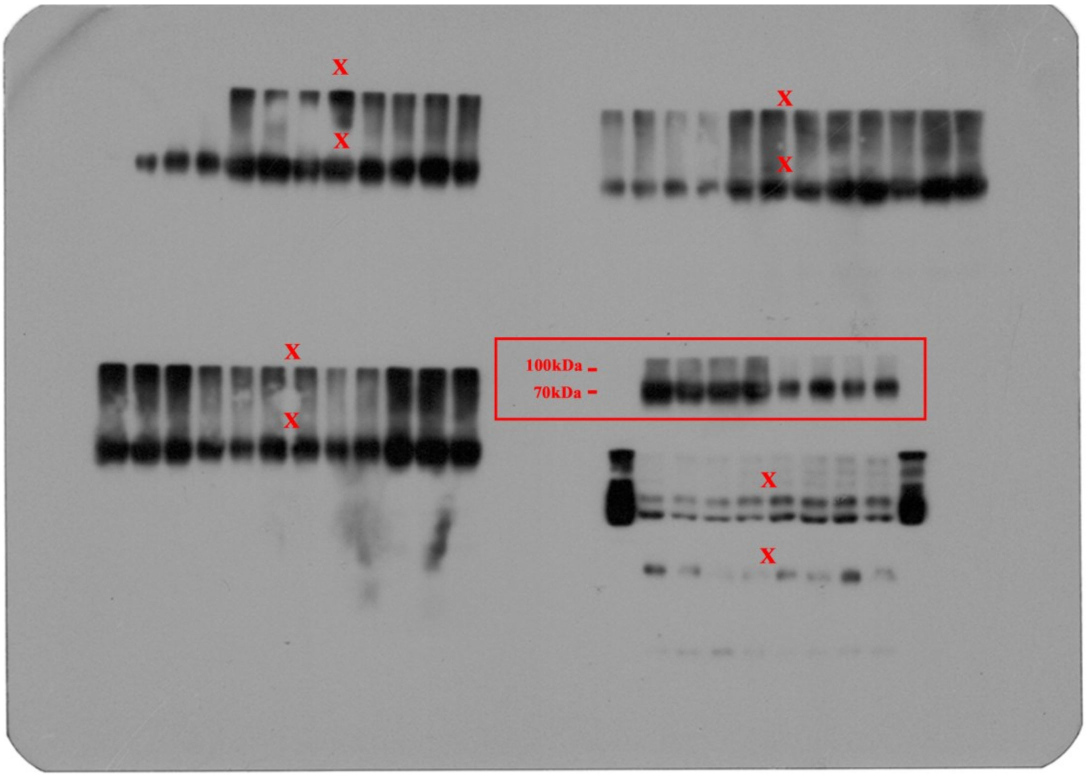

Figure S6.D-Rgs7 (HEK293T cells)

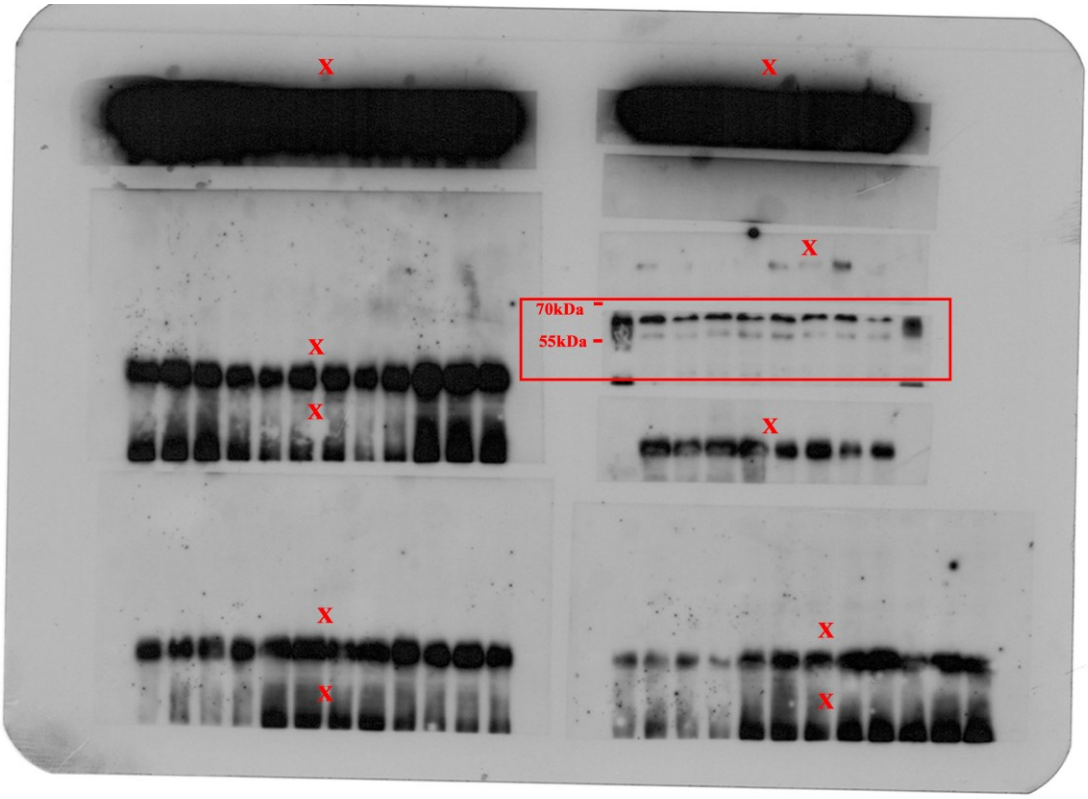

Figure S6.D-Akt (HEK293T cells)

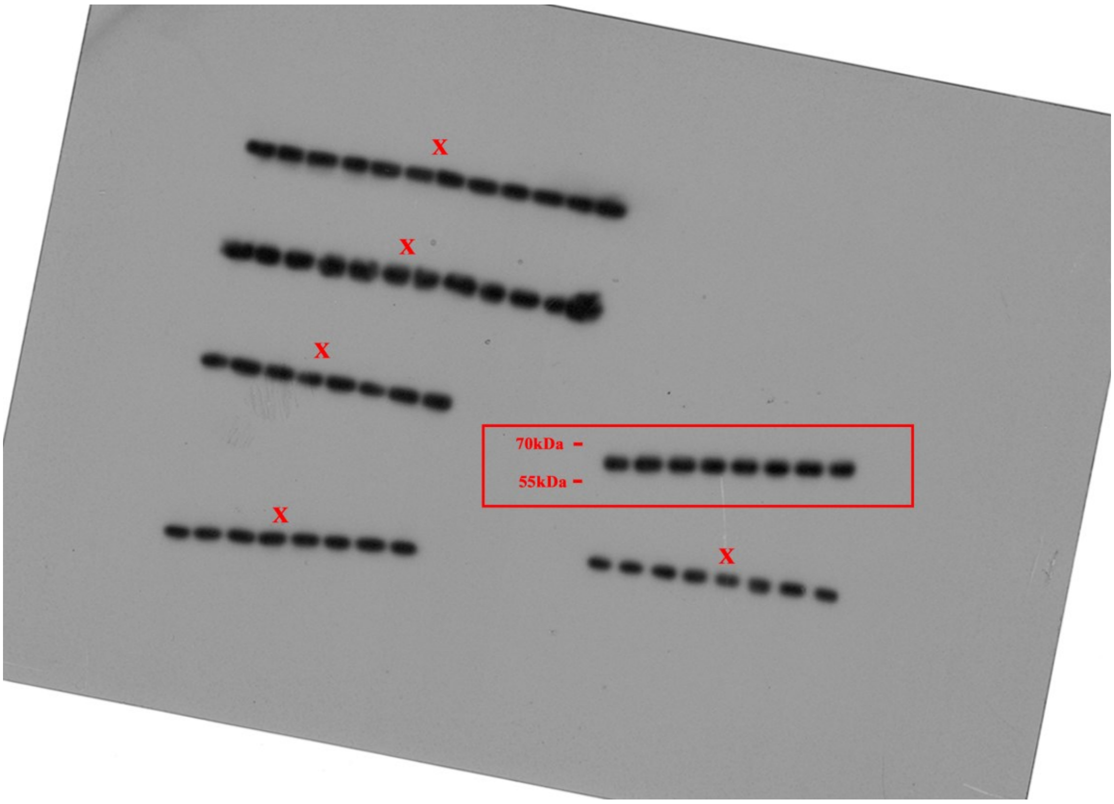

Figure S6.D-p-Akt (HEK293T cells)

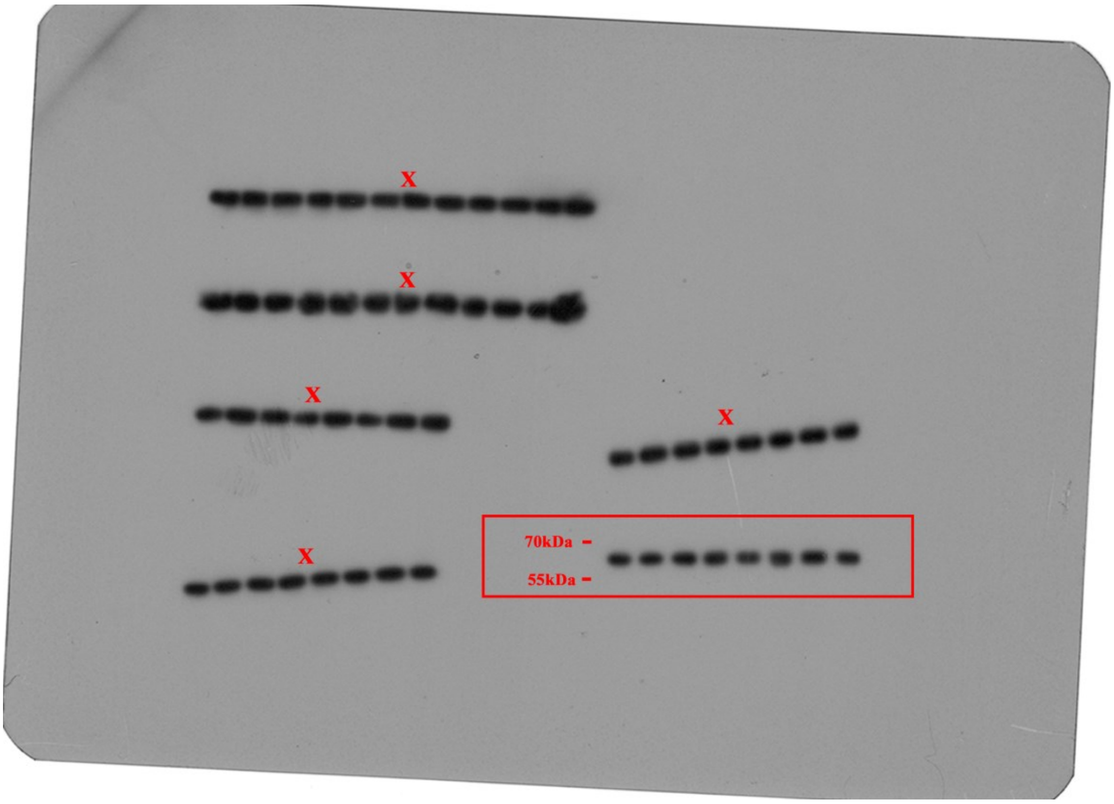

Figure S6.D-α-Tubulin (HEK293T cells)

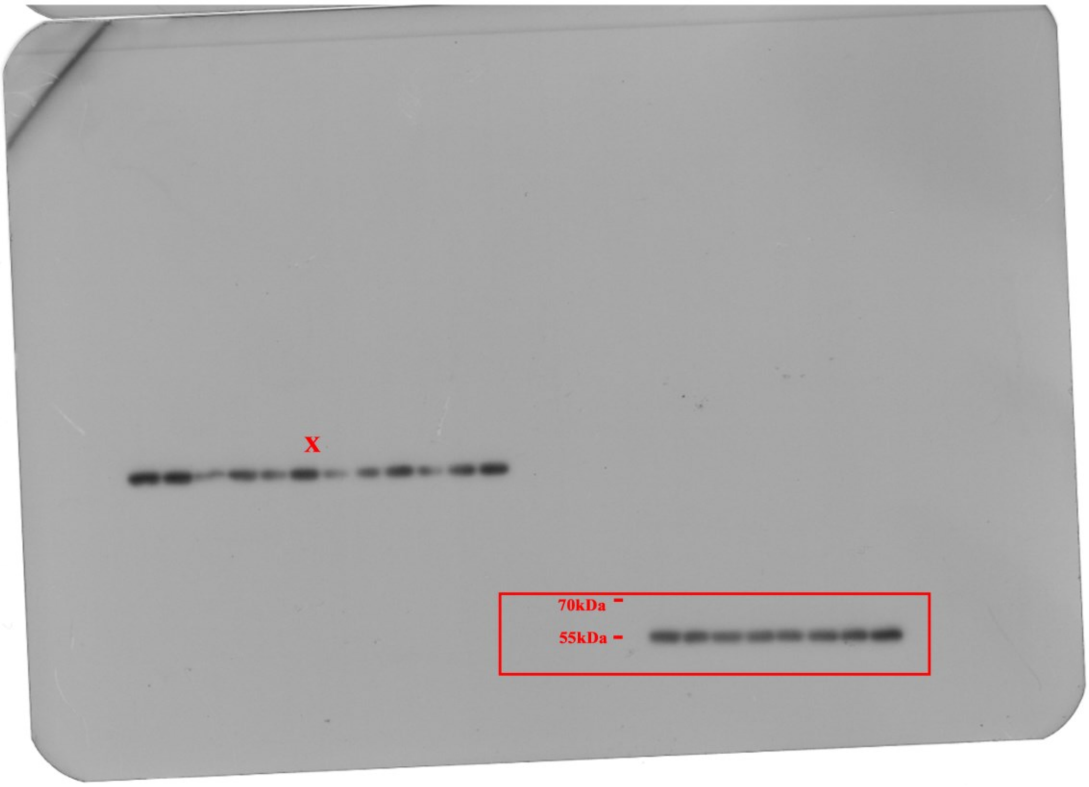

Supplement: S1 Raw Images — (PDF) [file pbio.3003259.s008.pdf]
