# Supplementary material for: Gnb5 is a negative regulator of the BACE1-mediated Aβ generation and ameliorates cognitive deficits in a mouse model of Alzheimer’s disease
Source: PLoS Biol. 2025 Jun 30;23(6):e3003259. doi: 10.1371/journal.pbio.3003259 (PMC12233908; doi:10.1371/journal.pbio.3003259)
Supplement: Attachment — Submitted filename: Response letter to reviewers.docx [file pbio.3003259.s010.docx]

**Response Letter to Reviewer Comments**

The authors would like to thank the efforts of the editorial personnel and the reviewers. The reviewer comments have been numbered and **in black**, with our responses provided **in blue**. The sections of the manuscript that were revised are marked **in red**.

Dear Dr Zhang,
> Thank you for your patience while your manuscript "Gnb5 ameliorates cognitive deficits by reducing β-amyloid deposition through BACE1 in Alzheimer's disease" was peer-reviewed at PLOS Biology. It has now been evaluated by the PLOS Biology editors, an Academic Editor with relevant expertise (who provided comments as Reviewer 1), and by several independent reviewers.
> As you will see below, the reviewers find the study potentially interesting, but they have raised a number of important concerns. The reviewers raise concerns with data presentation, statistical rigor, and mechanistic clarity and identify issues with the western blot analyses, behavioral data, and quantifications. We think these points will need to be thoroughly addressed before we can consider your paper further for publication. I note that reviewer 3 has also raised concerns about the mouse model used here and suggests that a knock in mouse model would be more relevant to Alzheimer's Disease. While we think this is an important point - we do not think that additional mouse models are needed here to validate the findings. Instead, we think that you should include explicit discussion of the limitations of the 5xFAD model. As a last point, we would like to emphasize the need to clarify the western blot data related to BACE1. As BACE1 can appear in both mature and immature forms, we think it will be important to clarify which forms are being measured in your analyses and if this has not yet been considered, you may need to add new data to flesh out these quantifications.

***Response:***

Thank you sincerely for your comprehensive feedback and for guiding us through this rigorous review process. We are deeply grateful to you and the reviewers for recognizing the potential significance of our work while providing constructive suggestions to enhance its scientific impact. And we revised and improved the manuscript as your and the reviewer’s suggestions as follows:

(1) The concerns raised regarding western blot analyses, behavioral data quantification, and mechanistic clarification are addressed in the point to point response section.

(2) Regarding the AD mouse model concerns, we incorporated an expanded discussion (page 17, lines 366-372) contextualizing the 5xFAD model's translational relevance to Alzheimer's Disease progression, explicitly addressing its strengths and limitations.

(3) Regarding the BACE1 WB analyses, we confirmed that the BACE1 (D10E5) Rabbit monoclonal antibody (#5606, Cell Signaling Technology) used in our study specifically recognizes the mature form of BACE1. In addition, we have repeated the BACE1 western blot analyses (BACE1 WB complete membrane images presented below, with molecular weight markers indicated on the left margin). The results demonstrate a distinct band at ~70 kDa, corresponding to the predicted molecular weight of mature BACE1. This combined evidence from both manufacturer validation and our experimental replication establishes the antibody's specificity for the mature BACE1 isoform.

**BACE1 WB** **complete membrane images of revised Figure 7E**

Figure Legends: Representative Western blot images showing the effects of viral-delivered full-length Gnb5, truncated fragments, and mutant truncated fragments on BACE1 expression in 5xFAD mice. The BACE1 immunoreactive bands demarcated by red rectangles have been quantitatively analyzed and presented in the revised Figure 7E.

**Point-to-point response to reviewers**

**Reviewer #1/Academic Editor:**

Gnb5 is a component of G protein-coupled receptors (GPCRs) and plays a significant role in intracellular signaling. Recent GWAS studies have indicated that Gnb5 variants are associated with the onset of Alzheimer's disease (AD). However, the precise mechanism by which Gnb5 influences AD pathology remains unclear.

This study demonstrates that Gnb5 contributes to AD pathology by regulating BACE1 expression, thereby affecting Aβ generation. Specifically, Chen et al. show that overexpression of Gnb5 improves cognitive function and reduces amyloid plaque loads in AD mouse models, while Gnb5 knockdown leads to the opposite effects. The authors also identify the specific binding domains of Gnb5 (WD-40), with which it directly interacts with BACE1. In light of these findings, it would be beneficial to provide additional evidence to further clarify these results.
***Response:***

Thank you sincerely for your thoughtful evaluation of our manuscript and we are grateful for the opportunity to enhance the clarity and impact of our findings through this revision process.

> 1. Chen et al. suggest that overexpression of Gnb5 reduces BACE1 levels, whereas knockdown of Gnb5 leads to increased BACE1 expression (Figure 5A-F). The authors are encouraged to include mRNA expression levels of BACE1 to clarify whether Gnb5 overexpression or knockdown influences BACE1 gene expression at the transcriptional level. It is required to determine whether Gnb5 affects BACE1 transcription or stability.

***Response:***

To address whether Gnb5 expression modulates BACE1 at the transcriptional level, we performed RT-PCR analysis on RNA samples from both Gnb5-overexpressing and Gnb5 conditional knockout models. Key findings include:

1. Delivery of Gnb5-AAV in 5xFAD and Wild-type mice showed no significant changes in BACE1 mRNA levels, *as shown in Response Figure 1A.*
2. Conditional deletion of Gnb5 (Gnb5^F/F^&CamKIIα-Cre, Gnb5-CCKO) in mice similarly did not alter BACE1 mRNA expression compared to Gnb5^F/F^ controls, *as shown in* *Response Figure 1B.*

**Response Figure 1:** **(Now be added to Figure S5 in the revised manuscript)**

Figure Legends: **(A)** The mRNA expression level of BACE1 in hippocampal tissues from 5xFAD and Wildtype mice following Gnb5 overexpression mediated by AAV vectors; Data are presented as mean ± SEM; Statistical significance was determined by one-way ANOVA with Holm-Šidák post hoc tests for multiple comparisons; *NS:*(P>0.05); **(B)** The mRNA expression level of BACE1 in hippocampal tissues from Gnb5-CCKO mice; Data are presented as mean ± SEM; Statistical significance was determined by two-tailed Student’s *t*-test; *NS:*(P>0.05);

> 2. The data in Figure 6 confirm that the 28-102 amino acid region of Gnb5 is the critical site for binding to BACE1. One important aspect that warrants further exploration is the potential bidirectional relationship between Gnb5 and BACE1. Specifically, the interaction between Gnb5 and BACE1 may not only regulate BACE1 expression but also modulate Gnb5 activity and its downstream signaling. It would be valuable to explore how BACE1 binding to Gnb5 influences Gnb5 activity, and whether this modulation in turn impacts downstream signaling pathways, which could further affect BACE1 expression. Investigating this feedback loop could provide a deeper understanding of the molecular mechanisms at play in AD pathology.

***Response:***

As highlighted in previous studies, Rgs7 serves as an essential binding partner of Gnb5 that participates in mediating Gnb5-dependent downstream cascades (Cites: PMID: 36996198, PMID: 34815401, PMID: 34793198). Notably, reduced Gnb5 expression levels have been shown to significantly suppress Akt signaling pathway activation, which represents a critical downstream pathway of Gnb5 (Cites: PMID: 36831001, PMID: 30719834, PMID: 30892828). Based on this, we focused on examining the potential regulatory effects of this interaction on Rgs7 and Akt expression patterns.

Given that serine 81 within the Gnb5 (28-102aa) domain constitutes the key binding site for BACE1, we performed comparative analyses of Rgs7, Akt, and p-Akt expression in samples injected with either Gnb5(28-102aa)-AAV or the S81L mutant AAV (Gnb5(28-102aa&p81L)). Western blot analyses revealed no significant alterations in Rgs7, Akt, or p-Akt protein levels following S81L mutation (*as shown in Response Figure 2.A-B*).

To further validate these findings, we designed three independent BACE1-targeting siRNAs and identified siR1-BACE1 (showing 75% knockdown efficiency, *as shown in Response Figure 2.C*) as the optimal candidate for subsequent experiments. Consistent with Gnb5 mutation studies, BACE1 knockdown in HEK293T cells for 24 hours similarly failed to induce significant changes in Rgs7, Akt, or p-Akt expression levels (*as shown in Response Figure 2.D-E*).

Based on this experimental evidence, we conclude that within our experimental system, Gnb5 primarily influences Aβ pathology in Alzheimer's disease through its regulatory effects on BACE1 expression activity, rather than via feedback modulation of its downstream effectors Rgs7 and Akt.

**Response Figure 2: (Now be added to Figure S6 in the revised manuscript)**

Figure legends: **(A)** Representative Western blot images showing the effects of viral-delivered Gnb5 truncated fragments or mutant truncated fragments on Rgs7, Akt and p-Akt expression in 5xFAD mice; **(B)** Quantitative analysis of the Western blot data shown in panel A; **(C)** qPCR analysis was performed to evaluate the knockdown efficiencies of three BACE1-targeting siRNAs in HEK293T cells. siR1-BACE1 (siRNA#1) demonstrated the most potent silencing effect and was selected for subsequent experiments **(D)** HEK293T cells transfected with siR1-BACE1 to assess the protein levels of BACE1, Rgs7, Akt, p-Akt and α-Tubulin served as a loading control; **(E)** Quantitative analysis of the Western blot data shown in panel D. Data are presented as mean ± SEM; Statistical significance was determined by two-tailed Student's t-test; NS:(P>0.05); **P< 0.01; ***P< 0.001;

> 3. Some of the western blot results, particularly for BACE1 (Figure 7E), require clarification. The authors should provide clearer images of the BACE1 western blots, as the current images may impact the accuracy of the quantification results.

***Response:***

To address this concern, we have performed new western blot analyses using freshly prepared hippocampal tissue lysates (n=3 per group). And, we successfully distinguished a clearer 70 kDa BACE1 band with enhanced clarity and signal-to-noise ratio through systematic optimization of antibody dilution (primary 1:1000, secondary 1:5000).

The updated Western blot images have been incorporated into main Figure 7E, and a normalized quantitative comparison relative to the α-Tubulin is presented in Figure 7F.

**Revised Figure 7.E-F:**

Figure legends: **(E-F)** Effect of overexpressing full-length Gnb5, truncated fragments, and mutant truncated fragment viruses on the expression of proteins related to APP cleavage process in 5xFAD. Representative western blot images **(E)** and statistical analysis of protein levels **(F)**; n=3, respectively; Data are presented as mean ± SEM; Statistical significance was determined by one-way ANOVA with Holm-Šidák post hoc tests for multiple comparisons; NS:(P>0.05); **P< 0.01; ***P< 0.001.

> 4. Given the importance of BACE1 in AD, enzymatic activity assays would provide valuable insights into its functional role. If fluorescence-based or colorimetric assays were conducted to assess BACE1 activity, including those experimental details would strengthen the study.

***Response:***

We sincerely appreciate the reviewer's insightful suggestion regarding BACE1 enzymatic activity analysis.

To address this critical gap, we have conducted fluorescence resonance energy transfer (FRET)-based BACE1 activity assays using the Beyotime Biotechnology Kit (P0362S), with full methodological details added to the *Materials and Methods* section (page 20, lines 433-448).

Our analyses revealed two critical observations:

1. 5xFAD mice exhibited significantly elevated BACE1 activity compared to controls (*p<0.0001*). Importantly, Gnb5-AAV overexpression in 5xFAD mice substantially reduced BACE1 enzymatic activity to near-normal levels (*p<0.0001 vs 5xFAD controls,* *as shown in Response Figure 3.A-B*).
2. Conversely, Gnb5 knockdown in 5xFAD mice further exacerbated BACE1 hyperactivity (*p<0.001*, *as shown in Response Figure 3.C-D*), systematically validating Gnb5's regulatory role in BACE1 function.

The newly added data not only confirm our initial conclusions but also provide a more comprehensive characterization of the Gnb5-BACE1 axis in AD pathology.

**Response Figure 3: (Now be added to Figure S5 in the revised manuscript)**

Figure Legends: **(A)** Real-time monitoring of BACE1 activity in Gnb5-overexpressing 5xFAD mice; Fluorescence intensity of BACE1-mediated fluorogenic products was measured in hippocampal tissues from 5xFAD mice receiving AAV-mediated Gnb5 overexpression; Kinetic readings were recorded at 10-minute intervals over a 60-minute assay period; **(B)** Quantitative analysis of BACE1 enzymatic activity (nmol/min/mg) from panel A; n = 6 mice/group; Data are presented as mean ± SEM; Statistical significance was determined by one-way ANOVA with Holm-Šidák post hoc tests for multiple comparisons; ****P < 0.0001; **(C)** Real-time monitoring of BACE1 activity in Gnb5-deficient 5xFAD mice; Tamoxifen-inducible Gnb5 knockdown was performed in hippocampal tissues of 5xFAD mice, with fluorescence intensity of BACE1-generated products quantified every 10 minutes for 60 minutes; **(D)** Quantitative analysis of BACE1 enzymatic activity (nmol/min/mg) from panel C; n = 4 mice/group; Data are presented as mean ± SEM; Statistical significance was determined by two-tailed Student’s *t*-test; ***P < 0.001.

> 5. The IBA1 immunohistochemistry images do not seem to fully align with the trends

shown in the corresponding quantification graph (Figure S4). If alternative images are available that could replace the current ones, it would enhance the interpretation of Gnb5's role in the pathology.

***Response:***

We sincerely appreciate the reviewer's meticulous observation and constructive feedback. To address this concern, we have:

1. Re-performed Iba1 immunohistochemistry in *Gnb5* conditional knockout mice using additional biological replicates (n=3/group).
2. Conducted blinded re-quantification with two independent investigators to ensure analytical rigor.
3. Provided updated representative images that now exhibit alignment with the statistical trends. (Revised Figure S4.C-D as showed bellowed as well)

**Revised Figure S4.C-D:**

Figure legends: **(C)** Immunostaining of Gnb5 together with Iba1 in the hippocampus of 6-month-old Gnb5^F/F^ and Gnb5 excitatory neuron (CamKIIα-Cre) conditional knockout mice; **(D)** Statistical analysis of the number of microglia, individual data points represent mean intensity values per mouse derived from 3-5 averaged fluorescence images; Data are presented as mean ± SEM; Statistical significance was determined by two-tailed Student's t-test; ****P< 0.01

> Reviewer #2: The authors show that reduced Gnb5 levels are associated with Alzheimer's disease (AD) and that overexpression of Gnb5 in an AD mouse model leads to reduced BACE1 levels and reduced Aβ-containing plaques. They also generated a conditional Gnb5 knockout mouse to characterize its effect on cognition, and they crossed it to the AD mouse model to further modulate Gnb5 levels. The generation of these transgenic mouse models is a significant strength of the study in the investigation of Gnb5 function. The role of the single mutation in Gnb5 in the interaction with BACE1 is also intriguing, however the relationship between that finding and what has already been shown about the S81L mutation in brain function was unclear. Additional concerns are detailed below:

***Response:***

Thank you sincerely for your comprehensive evaluation of our study and for acknowledging both its strengths and areas requiring further development.

> * The initial results suggest that pathogenic Aβ causes a decrease of Gnb5 in 6-month-old 5xFAD mice, but then the following figures focus on how changing Gnb5 levels affect Aβ levels. There is a circular effect of Aβ-Gnb5- Aβ that is not discussed in the manuscript. It could essentially be a chicken and egg problem in terms of what is the most important signaling mechanism involved.

***Response:***

Thank you for your exceptionally insightful critique and for identifying this crucial conceptual dimension of our work.

Our experimental data reveal two key phenomena: (1) Aβ deposition (potentially in conjunction with other factors) drives Gnb5 downregulation in 6-month-old 5xFAD mice, consistent with observations in human AD brains. (2) Subsequent mechanistic investigations reveal that both knockdown (Figure 4.D-G) and overexpression (Figure 4.A-C) of Gnb5 significantly modulate Aβ levels, suggesting a regulatory feedback mechanism. These evidence supports our hypothesis that AD progression may involve a self-reinforcing loop: Aβ pathology primarily drives Gnb5 reduction (Figure 1), which subsequently exacerbates Aβ deposition through BACE1 regulation (as shown in Figures 5-6), while accumulated Aβ may further aggravate Gnb5 downregulation (supported by in vitro evidence in Figure S1.L), ultimately establishing a vicious feedback cycle.

We fully agree that this circular relationship presents a "chicken-and-egg" dilemma regarding AD progression drivers. To address this, we added a dedicated discussion section (page 15, lines 313-322) analyzing this bidirectional regulatory feedback loop.

> * A neuronal marker should be used to establish whether or not the change in Gnb5 shown in Figure 1K is happening in neurons. The quantification and statistical analysis performed in Figure 1L should be done comparing each mouse per group, not a comparison across multiple individual images taken from each mouse.

***Response:***

We sincerely appreciate the reviewer's insightful comments and constructive suggestions.

To address this, we performed co-staining experiments in mouse brain sections using Gnb5 with cell-type specific markers: NeuN (neurons), Gfap (astrocytes), and Iba1 (microglia). *As shown in Response Figure 4.A* (now be added to Figure S2.E in the manuscript), Gnb5 demonstrates predominant neuronal localization. This finding is consistent with our qPCR and Western blot data showing higher Gnb5 expression in neuronal fractions (Figure S2.B-D). Collectively, these analyses confirm that Gnb5 is primarily expressed in neurons.

Furthermore, we performed triple labeling of NeuN, Gnb5, and Thioflavin-S (Thio-S) in brain sections from both 5xFAD and Wild-type mice. The 5xFAD group demonstrated a marked reduction in Gnb5 expression, particularly evident in the overall neuronal expression pattern, with a pronounced deficiency observed in regions containing Aβ plaques (*As shown in* *Response Figure 4.B*).

Regarding the statistical presentation, we revised the statistical approach as follows: Each data point in *Response Figure 4.C* now represents the average fluorescence intensity per mouse (n=4 mice/group, with 4-6 images analyzed per mouse). We also revised the Figure 1L.

**Response Figure 4: (Now be added to Figure S2 in the revised manuscript)**

Figure legends: **(A)** Immunofluorescence analysis showing co-localization of Gnb5 with neural cell-specific markers: neuronal marker NeuN, astrocytic marker Gfap, and microglial marker Iba1; **(B)** Representative confocal images showing triple labeling of Gnb5, NeuN, and Thioflavin-S (Thio-S) in both 5xFAD and wildtype mouse brain sections; White stars denote regions containing Aβ plaques; **(C)** Quantitative analysis of Gnb5 fluorescence intensity in 5xFAD and Wildtype groups (n = 4 mice/group). Individual data points represent mean intensity values per mouse derived from 4-6 averaged fluorescence images. Data are presented as mean ± SEM; Statistical significance was determined by two-tailed Student’s t-test; ***P< 0.001;

> * The western analyses of Gnb5 in Figure S2 are not convincing. There was no information on how the different cell types were cultured to make them pure neurons or astrocytes or microglia. That information should be added to the methods and pointed out in the results. Immunocytochemistry in cultures of immunohistochemistry in brain sections (as in Figure 1K) should be used with cell-specific markers to confirm these findings.

***Response:***

Thank you for your careful evaluation of our work and for highlighting these critical points to strengthen the rigor of our study.

We have now added comprehensive details of our procedures to the Supplementary Information section (supplementary page 3, line 65-90). Our protocols were based on established methodologies refined by our laboratory [DOI: 10.1016/j.neuron.2018.08.031].

As recommended, we performed immunofluorescence on mouse brain sections using antibodies against Gnb5 alongside cell-type-specific markers: NeuN (neurons), Gfap (astrocytes), and Iba1 (microglia). These experiments revealed a striking colocalization of Gnb5 with NeuN in neuronal populations. In contrast, minimal overlap was observed with Gfap or Iba1 signals, supporting the neuron-predominant expression pattern of Gnb5 (*As shown in Response Figure 4.A*).

> * It is important to explicitly acknowledge in the results section for Figure 2 that the Gnb5-CCKO mice showed a marked impairment in spatial learning in Figure 1F. This is consistent with their poor performance in the spatial memory test (Figure 2G-I) because the mice did not learn the target location during training. The authors do not point out this important distinction between spatial learning and spatial memory.

***Response:***

Thank you for your attentive review and for highlighting this critical distinction between spatial learning and spatial memory in interpreting our behavioral data.

We have revised the Results section (page 7, lines 147-154) to explicitly state that the impaired performance of Gnb5-CCKO mice in the Morris water maze training phase (Figure 2.F) reflects a spatial learning deficit, which directly contributes to their poor performance in the subsequent probe trials (Figure 2.G-I).

> * Freezing measurements during conditioning in Day 1 should be shown including baseline freezing for experiments in Figures 2 and 3. To establish that the effect is due to memory, it needs to be established whether the mice have freezing differences during the training period.

***Response:***

Regarding baseline freezing during fear conditioning training (Day 1), we analyzed and concluded below:

1. Gnb5 conditional knockout mice (Figure 2): *As shown in the newly added Figure 2.L*, Gnb5^F/F^&CamkIIa-Cre mice exhibited no significant difference in freezing time during Day 1 training compared to Gnb5^F/F^ controls (P=0.804, by two-tailed Student’s *t*-test).

2. Gnb5 overexpression in 5xFAD mice (Figure 3): Similarly, 5xFAD mice overexpressing Gnb5 showed comparable freezing levels to AAV-control 5xFAD mice during training (P=0.2969, by two-tailed Student’s *t*-test; *As shown in the newly added Figure 3.I*).

These data, now integrated into Results section (lines 154-156 and lines 186-187), confirm that the observed differences in post-training freezing arise from memory-specific mechanisms rather than variations in initial learning, shock sensitivity, or locomotor function.

**Revised Figure 2.L, Figure 3.I**

Figure legends: The baseline freezing measurements during conditioning in Day 1 for experiments in Figures 2.L and Figure 3.I.

> * The green and purple groups in Figure 3F have a star suggesting a statistically significant difference, but this is not convincing because they look similar and no information on statistical analyses were provided. In Figure 3G, the average % time that control mice spent in the target quadrant is about 20% which is lower than the chance rate of 25% for each target. Thus, the control mice did not have a preference for the target quandrant, and consequently, this is not an acceptable measurement of spatial memory in this experiment. Since the controls did not show intact spatial memory as measured by % time in the target quadrant, it is then impossible to evaluate memory performance in the AD mice using the data in Figure 3G.

***Response:***

**Question 1:**

We sincerely appreciate your rigorous evaluation of our work and your insightful identification of the statistical interpretation issue in Figure 3F (now Figure 3D). Upon re-examining the original dataset, we acknowledge that an inappropriate statistical method (Student's t-test) was initially employed, which resulted in erroneous single-asterisk annotations.

In accordance with your expert recommendation, we have conducted a comprehensive reanalysis using appropriate parametric testing (one-way ANOVA with post hoc comparisons). Importantly, we have both revised the statistical methodology and enhanced the experimental design by increasing the sample size (N) in this updated figure. These substantial improvements are now incorporated in the revised manuscript (current Figure 3D).

**Question 2:**

We sincerely appreciate the reviewer's insightful comments regarding Figure 3G (now renumbered as Figure 3E). In response to this concern, we would like to clarify that our Morris water maze experiments were strictly conducted in accordance with established protocols. We fully acknowledge that the control group's 20% preference for the target quadrant fell below the theoretical chance level of 25% and this could be due to the sample size.

To address this concern, we conducted an independent behavioral validation experiment with expanded sample sizes as follows: Wildtype + AAV-GFP (n=10, now total is 20), Wildtype + AAV-Gnb5-GFP (n=11, now total is 23), 5xFAD + AAV-GFP (n=12, now total is 23), 5xFAD + AAV-Gnb5-GFP (n=13, now total is 26). The behavioral testing results of the newly incorporated samples are shown in Response Figure 5. Subsequently, we performed integrated analysis of two independent experimental datasets, as presented in the *revised Figure 3D-M*. The consolidated datasets consistently demonstrate that AAV-mediated Gnb5 overexpression produces significant improvements in cognitive deficits observed in 5xFAD mice (consistent with our original findings).

**Response Figure 5:**

Figure legends: **(A-E)** MWM tests in mice; Wildtype+AAV-GFP, n=10; Wildtype+AAV-Gnb5-GFP, n=11; 5xFAD+AAV-GFP, n=12; 5xFAD+AAV-Gnb5-GFP, n=13; **(A)** Training curve in the MWM; **(B)** Percentage of time spent in the target quadrant in the MWM; **(C)** Statistical analysis of the number of entries in the target quadrant in the MWM; **(D)** Schematic diagram of mouse movement trajectory in the MWM; with quadrants marked and platform locations indicated by circles; **(E)** Movement speed of mice in the MWM; **(F)** Total distance traveled in the open field test; **(G)** Percentage of time spent in the center area during the open field test. Data are presented as mean ± SEM; Statistical significance was determined by one-way ANOVA with Holm-Šidák post hoc tests for multiple comparisons; NS:(P>0.05); *P < 0.05; **P < 0.01; ***P < 0.001. **(I-K)** The contextual fear conditioning test in mice. **(I)** Baseline freezing percentage of mice in an identical testing context**; (J)** Percentage of freezing time of mice during the Context phase; **(K)** Percentage of freezing time of mice during the Cue phase. Data are presented as mean ± SEM; Statistical significance was determined by two-tailed Student’s t-test; NS:(P>0.05); *P< 0.05;

**Revised Figure 3D-M:**

Figure legends: **(D-H)** MWM tests in mice; Wildtype+AAV-GFP, n=20; Wildtype+AAV-Gnb5-GFP, n=23; 5xFAD+AAV-GFP, n=23; 5xFAD+AAV-Gnb5-GFP, n=26; **(D)** Training curve in the MWM; **(E)** Percentage of time spent in the target quadrant in the MWM; **(F)** Statistical analysis of the number of entries in the target quadrant in the MWM; **(H)** Movement speed of mice in the MWM; **(I-K)** The contextual fear conditioning test in mice. **(I)** Baseline freezing percentage of mice in an identical testing context**; (J)** Percentage of freezing time of mice during the Context phase; **(K)** Percentage of freezing time of mice during the Cue phase; Data are presented as mean ± SEM; Statistical significance was determined by two-tailed Student’s t-test; NS:(P>0.05); ***P< 0.001; **(L-M)** The open field test in mice. **(L)** Total distance traveled in the open field test; **(M)** Percentage of time spent in the center area during the open field test. Data are presented as mean ± SEM; Statistical significance was determined by one-way ANOVA with Holm-Šidák post hoc tests for multiple comparisons; NS:(P>0.05); *P < 0.05; ***P < 0.001; ****P < 0.0001.

> * Figure S3 and S4 are missing from the results. They should be described in the results, not in the Discussion. The SNAP25 in Figure 3 is way too saturated for reliable quantification. It looks like Gnb5 caused an abnormal reduction of the GluN1 subunit of NMDA receptors and Syntaxin compared to control mice. Thus, it should not be concluded that the "…overexpression of Gnb5 in AD mice mitigated the abnormal expression of these synaptic proteins".

***Response:***

Regarding your concerns about the presentation of data in Figure S3/S4 and the synaptic protein alterations in Figure 3A, we would like to provide the following clarification: In this study, we primarily focused on demonstrating the therapeutic effects of Gnb5 overexpression on cognitive behavioral phenotypes and Aβ deposition in 5xFAD mice. The synaptic protein analysis was initially included as a supplementary investigation to explore potential secondary effects of Gnb5 overexpression. However, as correctly noted in your review, the results revealed partial restoration of synaptic proteins accompanied by some unexpected expression patterns that deviate from normal physiological levels.

After careful consideration, we agree that these findings require more systematic investigation into the underlying pathological mechanisms. To maintain focus on the central theme of Aβ plaque modulation and enhance the clarity of presentation, we have decided to remove this data from the current manuscript. This will allow readers to better concentrate on the primary findings while avoiding potential confusion from incomplete mechanistic explanations.

We fully acknowledge the importance of understanding synaptic function alterations in AD pathology, and this valuable suggestion has been incorporated into our future research plans. Our team is currently designing comprehensive experiments to specifically investigate the molecular mechanisms by which Gnb5 regulates synaptic plasticity, which will form the basis of our next research article.

> * The tubulin blot in Figure 5A is markedly uneven which is concerning because it was used for normalization in the quantification.

***Response:***

To address this concern, we repeated the Western blot analysis using freshly prepared hippocampal tissue lysates (n=4 per group) following standardized experimental protocols. All target proteins in Figure 5A along with α-Tubulin were detected, and protein quantification was re-performed. The updated representative Western blot images and corresponding statistical analyses have been incorporated into Revised Figures 5A and 5B. Importantly, this independent replication yielded experimental outcomes consistent with our original observations, thereby confirming the initial conclusions.

**Revised Figure 5A-B:**

Figure legends: **(A-B)** Overexpression of Gnb5-AAV in 5xFAD mice affects the expression of proteins related to APP cleavage (BACE1, β-CTF, Nicastrin and APP); **(A)** Representative Western blot data; **(B)** Statistical analysis of protein levels; n=4, respectively; Data are presented as mean ± SEM; Statistical significance was determined by one-way ANOVA with Holm-Šidák post hoc tests for multiple comparisons; *NS:*(P>0.05); ****P< 0.01; ***P< 0.001; ****P< 0.0001;

> * A major issue that needs to be addressed in this manuscript is that the type of statistical analyses used (for example one-way ANOVA or t-test) needs to be added to the figure legends for every individual graph that shows statistics.

***Response:***

We have now incorporated the analytical details (e.g., one-way ANOVA, t-test) into each relevant figure legend.

> * It is unclear what the following sentence means, and it needs to be clarified with more information. "Interestingly, the cognitive function related mutation site (p.Ser81Leu (S81L)) on Gnb5 also locates in this region [18, 19]"

***Response:***

We sincerely appreciate the reviewers' insightful feedback and regret any lack of clarity in our original presentation. To address the concerns raised, we provide the following clarifications:

Firstly, emerging evidence has established a connection between the Gnb5 p.Ser81Leu (S81L) mutation and cognitive functions. As demonstrated by Lodder et al. (DOI: 10.1016/j.ajhg.2016.06.025) in the American Journal of Human Genetics, this mutation causes an autosomal-recessive multisystem syndrome characterized by sinus bradycardia and cognitive disability. Furthermore, Shamseldin et al. (DOI: 10.1186/s13059-016-1061-6) in Genome Biology reported that the same mutation leads to a novel neuropsychiatric disorder presenting with attention deficit hyperactivity disorder and severely impaired language development and cognitive function. These findings collectively suggest the mutation's significant impact on neurological functions.

Moreover, we cited two studies confirming that the p.Ser81Leu mutation in Gnb5 resides within its first WD-repeat domain (references [18] and [19]; structural diagrams from these studies are now included in Figure [A/B] below). To improve clarity and precision, we have revised the sentence to: *"**Notably, the p.Ser81Leu mutation in Gnb5, which has been implicated in cognitive function alterations [18,19], is localized within the first WD-repeat domain of Gnb5 (specifically, the 28-102aa region)."* (page 12, lines 259-261)

**Figure A: (DOI: 10.1186/s13059-016-1061-6)**

**Figure B: (DOI: 10.3389/fgene.2018.00626)**

> * The groups in Figure 7C and D are not explicitly labeled. The graphs need a legend. Mice should be labeled as 5xFAD.

***Response:***

Thank you sincerely for your meticulous review.

We revised the figures as your suggestions:

1.	Added explicit group labels to both panels

2.	Standardized nomenclature to "5xFAD" throughout the figure and legend.

> * It is also unclear what the sentence means in lines 253-255 of the Discussion. Indeed, there is limited success for that strategy. Wouldn't this suggest that this strategy should not be developed as treatments for AD? In general, the Discussion section could include more information relating the findings to previous literature instead of restating results.

***Response:***

We appreciate your critical assessment of this section and apologize for the lack of clarity in our original phrasing (lines 253-255).

To better convey our intended message, we have rephrased the contentious statement as: "*In this context, our study identifies Gnb5, a brain-enriched Gβ subunit of the GPCR complex, as a novel negative regulator of BACE1-mediated Aβ generation and a critical modulator of AD progression.* " (page 14, lines 302-304)

Additionally, in response to your suggestion, we have revised the Discussion section to better integrate our findings with previous literature, rather than simply restating our results. Specifically, we have added new comparisons with relevant studies and further contextualized how our findings advance the current understanding of GPCR signaling, BACE1 regulation, and Alzheimer's disease pathology. (page 14, lines 307-312; page 16, lines 333-338)

> * The claim that "with heterozygosity of Gnb5 shown to exacerbate… in AD model mice" is incorrect. The two studies cited are human subjects studies, which show correlative effects. They did not show that Gnb5 exacerbates disease.

***Response:***

Thank you for bringing this important issue to our attention.

Regarding the first cited study (Zhang et al., AJHG 2024), we wish to emphasize that the authors not only identified GNB5 as an AD risk gene through innovative genomic constraint analysis but also conducted functional validation in AD model mice. Their experimental evidence demonstrates that Gnb5 haploinsufficiency exacerbates both amyloid plaque deposition and neurofibrillary tangle formation in these models. We have attached relevant figure panels from their publication for your reference and believe these findings robustly support our original statement about Gnb5 heterozygosity exacerbating AD pathology.

Concerning the second citation (Bayraktar et al., IJMS 2021), we agree with your assessment that this human transcriptomic study provides correlative rather than causative evidence. While their network analysis identified GNB5 as part of a synaptic gene network showing AD-associated dysregulation, we acknowledge that this does not establish direct involvement in disease pathogenesis. We have therefore removed this reference from our revised manuscript.

Based on this, we have modified the relevant text (page 14, lines 307-310): “*Recent genetic studies have implicated Gnb5 as a candidate risk gene for AD, with experimental evidence showing that heterozygous Gnb5 enhances amyloid plaque formation and neurofibrillary tangle development in AD model mice.*”

**Relevant figure: (DOI: 10.1016/j.ajhg.2024.01.005)**

> * The NOR test was in the methods but not in the paper.

***Response:***

Thank you for your careful review and for identifying this discrepancy in our manuscript. we have removed all references to the NOR test from the Methods section in the revised manuscript.

> * There are many grammatical errors throughout the manuscript including Lines 81-83,101, 146-148, 181-182, 293-297, Figure 7C and D y-axes. There were many more grammatical errors in the methods section which is poorly written with important missing information.

***Response:***

Thank you for your meticulous review and for identifying the grammatical inconsistencies and methodological gaps in our manuscript. We sincerely apologize for these oversights. Your feedback has been instrumental in improving the clarity, rigor, and reproducibility of our work. We have thoroughly addressed grammatical errors throughout the manuscript, including but not limited to:

- Lines 81-83: Revised to “*The mechanism involves Gnb5 binding to BACE1 and regulating its expression. The first WD repeat domain contains serine 81, which is essential for this interaction.*”
- Line 101(now: line 104): Revised to “*To further characterize the association of Gnb5 with late-onset Alzheimer’s disease (LOAD), we analyzed a large dataset (GSE44772)[29] containing extensive clinical data from AD patients. Our analysis confirmed that Gnb5 expression was reduced in the brain tissues of AD patients (Figure 1.F).*”
- Lines 146-148 (now: lines 166-169): Revised to “*Given the downregulation of Gnb5 in AD and its negative correlation with Braak stages (Figure S1.B), along with evidence that Gnb5 deficiency in excitatory neurons causes memory deficits (Figure 2), we sought to evaluate whether overexpressing Gnb5 could mitigate cognitive deficits in 5xFAD mice.*”
- Lines 181-182 (now: lines 206-207): Revised to “*The inducible Cre system avoids any developmental deficits that might arise from embryonic Gnb5 deletion.*”
- Lines 293-297 (now: lines 333-338): Revised to “*Therefore, identifying novel regulatory factors of BACE1 in a more selective and controlled manner could enhance the efficacy and safety of AD treatments that target BACE1 activity. Given its predominant neuronal expression, Gnb5 represents an attractive candidate for selectively modulating BACE1 activity within the brain, potentially minimizing off-target effects in peripheral tissues*.”
- Figure 7C/D y-axes: Revised to “*Aβ Plaque Load (% area)*”, “*Plaque number*”

And, we have thoroughly revised the Methods section to address grammatical inaccuracies and have provided additional details to ensure comprehensive documentation of our experimental procedures.

> * Minor: One of the images in Figure 4A is flipped in the opposite orientation.
***Response:***

Thank you. We have revised Figure 4A to correct the orientation of the Wildtype+AAV-GFP panel as your suggestion.

> Reviewer #3: This is in many ways an interesting paper but it leaves the reader with almost more questions than answers. There are many G-Protein-mediated responses in which beta subunits are involved. It is not clear whether the authors are suggesting that all the changes suggested in this paper to be caused by loss of or overexpression of this particular beta subunit are mediated via the same initial ligands/receptors. Alternatively, as changing the level of this beta subunit will affect many different pathways it could have a wide range of independent effects. Whether changing the level of this beta subunits sets off a chain of reactions related to BACE activity or a series of different unconnected effects is unclear. No doubt loss of or overexpression of this subunit may also affect many other phenomena.

***Response:***

We thank the reviewer for their thoughtful and constructive feedback. We agree that modulation of a G protein β subunit may influence multiple signaling pathways, given the widespread involvement of Gβγ dimers in GPCR-mediated signaling. In our study, we specifically focused on G protein subunit β5 (Gnb5), which we identified as significantly downregulated in both AD patient brains and AD mouse models.

While many Gβ subunits can participate in a variety of signaling cascades, our findings suggest that Gnb5 plays a selective and functionally important role in modulating BACE1 expression and Aβ generation, which are key contributors to Alzheimer’s disease (AD) pathogenesis. Specifically, we demonstrate that Gnb5 interacts directly with BACE1 and negatively regulates its expression. This interaction is functionally significant, as hippocampal overexpression of Gnb5 in 5xFAD mice reduces Aβ deposition and improves cognitive function. Furthermore, we show that this regulatory effect depends on the first WD domain of Gnb5 and the integrity of the Ser81 residue.

To address the potential influence on BACE1 activity, we specifically investigated whether alterations in Gnb5 levels could modulate BACE1 enzymatic activity (as addressed in our response to Comment 4 from Reviewer 1). Our experimental data demonstrated that Gnb5 expression significantly influences BACE1 activity levels, with the quantitative results presented in *Response Figure 3A-D* below.

We acknowledge the reviewer’s concern regarding whether the observed effects are mediated through a single upstream GPCR-ligand interaction or reflect broader consequences of Gnb5 dysregulation. Based on our current data, we do not conclude that all phenotypes are mediated through a single ligand/receptor pair. Instead, we propose that Gnb5 may act at a signaling hub where it exerts a selective influence on BACE1, likely through protein-protein interaction and possibly post-transcriptional regulation, independent of canonical GPCR activation.

Base on this, we have revised the Discussion section (page 17, lines 350–355).

**Response Figure 3: (Now be added to Figure S5 in the revised manuscript)**

Figure Legends: **(A)** Real-time monitoring of BACE1 activity in Gnb5-overexpressing 5xFAD mice; Fluorescence intensity of BACE1-mediated fluorogenic products was measured in hippocampal tissues from 5xFAD mice receiving AAV-mediated Gnb5 overexpression; Kinetic readings were recorded at 10-minute intervals over a 60-minute assay period; **(B)** Quantitative analysis of BACE1 enzymatic activity (nmol/min/mg) from panel A; n = 6 mice/group; Data are presented as mean ± SEM; Statistical significance was determined by one-way ANOVA with Holm-Šidák post hoc tests for multiple comparisons; ****P < 0.0001; **(C)** Real-time monitoring of BACE1 activity in Gnb5-deficient 5xFAD mice; Tamoxifen-inducible Gnb5 knockdown was performed in hippocampal tissues of 5xFAD mice, with fluorescence intensity of BACE1-generated products quantified every 10 minutes for 60 minutes; **(D)** Quantitative analysis of BACE1 enzymatic activity (nmol/min/mg) from panel C; n = 4 mice/group; Data are presented as mean ± SEM; Statistical significance was determined by two-tailed Student’s *t*-test; ***P < 0.001.

> In Alzheimer's disease there is a loss of synapses within and in the immediate vicinity of plaques and so one of the proteins or local expression systems affected may well be this beta subunit. It is notable that various alpha and at least one gamma subunit are also down. This is illustrated in Fig 1 K-L in which measuring the fluorescence in a field covered by a plaque in the 5xFAD mouse almost wipes out any Gnb5 as it may will be lost with the loss of synapses in that region whereas, whole tissue analysis with Western blots is less extreme. Indeed in the left hand panel of 1K the holes are visible where there are plaques but the background levels are not so different between WT and 5xFAD. Note that for the statistics shown in Fig 1L this is also pseudo replication. The sample size should be n=4 with separate observations from the same mouse averaged to give a single data point. Fig 1 suggests that the main beta subunits in the G-proteins of lost synapses is probably Gnb5 and that likely it is expressed locally within synapses.

***Response:***

We thank the reviewer for this insightful comment and the careful examination of Figure 1K–L. We agree with the reviewer that synapse loss in and around Aβ plaques may contribute to the apparent reduction of Gnb5 signal observed in the 5xFAD mouse brain. Indeed, our immunofluorescence reveals a marked depletion of Gnb5 signal in plaque-associated regions. We also analyzed the other regions which is not Aβ plaques deposition, and found that the Gnb5 is also reducing.

We also acknowledge the reviewer’s observation that whole-tissue Western blot analysis shows a less dramatic reduction in Gnb5 protein levels, as it reflects the average from entire brain regions including both plaque-containing and plaque-free areas. To this end, we performed triple labeling of NeuN, Gnb5, and Thioflavin-S (Thio-S) in brain sections from both 5xFAD and wild-type mice. The 5xFAD group demonstrated a significant reduction in Gnb5 expression that was evident both in Aβ plaque-containing and plaque-free regions (*As shown in* *Response Figure 4.B*).

Regarding the statistical analysis in Figure 1L. The original figure included multiple fluorescence images from the same mouse as independent data points, which indeed constitutes pseudo-replication. We have now reanalyzed the data by averaging measurements from multiple fluorescence images within each mouse, and using individual mice as the biological replicates (n=4 per group). The revised quantification is shown in the updated Figure 1L and reflects this correction. Statistical significance remains consistent with our original findings.

**Response Figure 4B &** **Revised Figure 1L:**

Figure Legends: **(Response Figure 4B)** Representative confocal images showing triple labeling of Gnb5, NeuN, and Thioflavin-S (Thio-S) in both 5xFAD and wildtype mouse brain sections; White stars denote regions containing Aβ plaques; **(Revised Figure 1L)** Quantitative analysis of Gnb5 fluorescence intensity in 5xFAD and Wildtype groups (n = 4 mice/group). Individual data points represent mean intensity values per mouse derived from 4-6 averaged fluorescence images. Data are presented as mean ± SEM; Statistical significance was determined by two-tailed Student’s t-test; ***P< 0.001;

> The observation that altering various G-protein-mediated pathways by altering the level of available subunits affects behaviour, is not surprising but there is little evidence here that this has anything to do with BACE or Alzheimer's disease. That some of these behaviours occur in the 5xFAD mice is probably unrelated to Alzheimer's disease. Like several overexpression models 5xFAD show various behavioural deficits but this is most likely a factor of overexpression of APP and may largely relate to the alpha side of the APP pathways. Note that the statement starting line 172 "The accumulation of Aβ in the brain is a hallmark of AD and is strongly associated with cognitive decline in patients ....." is clearly incorrect. Plaques are not well correlated with cognitive decline and indeed in human diagnosis only occurs once there is a substantial load of neurofibrillary tangles. Hence substantial cognitive deficits in mice with plaques but no tangles is unlikely to be related to the cognitive deficits of Alzheimer's disease. Overall, now that knock in technology is a decade old with mouse knock in models readily available, the ongoing use of transgenic overexpressing mice is rather unfortunate and limiting.

***Response:***

We thank the reviewer for raising these important points regarding the disease relevance of our findings and the use of the 5xFAD model. We would like to address each concern as follows:

**1. Relevance of Gnb5 to BACE1 and AD Pathology:**

While we agree that behavioral effects resulting from modulation of G-protein subunit levels may not be surprising in isolation, our study provides direct mechanistic evidence linking Gnb5 to BACE1 regulation. Specifically, we demonstrate that Gnb5 interacts with BACE1 and modulates its protein levels, thereby influencing Aβ generation. These findings go beyond a general effect on GPCR signaling or behavior, and establish a novel, functionally significant connection between a specific G-protein β subunit and the amyloidogenic processing pathway of APP. Furthermore, manipulation of Gnb5 levels in the hippocampus led to consistent changes in both Aβ burden and cognitive performance, strengthening the link between Gnb5, BACE1, and AD-related pathology.

**2. On the Use of 5xFAD Mice:**

We acknowledge the limitations of the 5xFAD model and the broader concerns regarding overexpression-based transgenic models. However, 5xFAD remains one of the most widely used and well-characterized amyloidogenic models, particularly suitable for investigating Aβ-dependent pathologies such as BACE1 regulation and plaque formation. In addition, we have updated the Discussion section (page 17, lines 366–372) to incorporate these points and appropriately frame the interpretation and limitations of our findings.

**3.Aβ and Cognitive Decline:**

We appreciate the reviewer’s correction regarding the complexity of the relationship between Aβ plaque burden and cognitive deficits. We have revised the sentence starting on line 197-198 to more accurately reflect the current understanding of AD pathology, now reading: “*Aβ accumulation remains a key initiating factor in the amyloid cascade hypothesis and contributes to synaptic dysfunction and neurotoxicity in animal models.*”

> In terms of the plaque data in Figure 4. Firstly, this is again a classic case of pseudoreplication. The sample size is 3, not around 18 as illustrated here. The results from the sections from the same mouse should be averaged to give a single value for each mouse. It is impossible to tell here how consistent the results within any mouse are. For example, the lowest values in the purple column in Fig 4B could all come from one mouse and the highes values in the green column from another with the remaining 4 mice (2 from each group having similar overlapping values). Similarly in figs 4F&G it is very likely that, particularly in the blue columns that the data fall into the 3 groups of the individual mice with one mouse tending to skew the data to a higher mean.

***Response:***

We thank the reviewer for the careful evaluation of our data presentation in Figure 4 and for pointing out the concern regarding pseudoreplication. We agree that using individual fluorescence images from the same mouse as independent data points without proper averaging can inflate the apparent sample size and lead to biased statistical interpretation.

In response to this valid concern, we have now re-analyzed all plaque burden and area quantification data in Figures 4B, 4F, and 4G, such that:

- Data from multiple fluorescence images within the same animal were first averaged,
- Then each mouse was treated as a single biological replicate (n=4 per group),
- And all statistical tests were re-performed accordingly.

We have updated the figures and statistical legends to reflect these corrected analyses. Despite the smaller apparent sample size, the trends remain consistent and the statistical significance is preserved in the key comparisons, indicating that the original conclusions are not an artifact of pseudoreplication.

**Revised Figure 4B/C/F/G:**

Figure Legends: **(B)** Quantification of Aβ plaques number; **(C)** Quantification of Aβ plaques size; **(F)** Quantification of Aβ plaque size; **(G)** Quantification of Aβ plaque number; Individual data points represent mean values of per mouse derived from 3-5 fluorescence images. Data are presented as mean ± SEM; Statistical significance was determined by one-way ANOVA with Holm-Šidák post hoc tests for multiple comparisons; **P< 0.01; ***P< 0.001; ****P< 0.0001.

> It is interesting that a beta subunit of a G-protein coimmunoprecipitates with BACE1. In this case Figs 5 and 6 the HEK cell experiment seem to suggest a direct binding of a particular domain of this G-protein subunit although how, in a physiological system, this would then relate to the activation of the G-protein by a set ligand or just a direct effect of this protein is unclear. Is the Ser81 of the beta subunit essential for the normal activation of the G-protein in the observations in Fig 7 or is this an effect related to the binding to BACE observed in HEK cells? Note the problem of pseudoreplication is again present in Fig 7 C&D.

***Response:***

We thank the reviewer for the insightful comments regarding the biochemical interaction between Gnb5 and BACE1 and its mechanistic interpretation. Below we addressed each point in detail:

**1. On the physiological relevance of Gnb5–BACE1 interaction and the role of classical G-protein activation:**

Our co-immunoprecipitation and domain mapping experiments in HEK293 cells (Figure 6) indicate the interaction between Gnb5 and BACE1, primarily mediated by the first WD domain of Gnb5. While HEK cells do not recapitulate the full physiological context of neuronal GPCR signaling, the direct binding observed suggests a ligand-independent interaction, rather than one that requires canonical G-protein activation via GPCRs. Notably, Gnb5 is part of the Gβ5–RGS complex, which is atypical in that it often acts in GPCR-independent cellular processes (Cites: PMID: 21511947, PMID: 18204463). Thus, we propose that the effect of Gnb5 on BACE1 may reflect a non-canonical scaffolding or regulatory function, rather than a downstream consequence of receptor-mediated G-protein activation.

**2. On the role of Ser81 in Gnb5 function:**

We appreciate the reviewer’s insightful question regarding whether the Ser81 residue is essential for canonical G-protein activation or is specifically involved in BACE1 binding. A similar point was also raised by Reviewer 1 (Comment 2), and we have provided a detailed response accordingly (as shown in Response Figure 2.A-B below). Our current data indicate that Ser81 is critical for Gnb5’s ability to regulate BACE1, as the S81L mutant failed to reduce BACE1 expression or Aβ deposition in 5xFAD mice. While we cannot entirely rule out a broader role of Ser81 in G-protein signaling, the fact that this residue is located near the BACE1 interaction interface, combined with its selective impact on the Gnb5–BACE1 pathway in our functional assays, strongly suggests that the observed effects are primarily due to disrupted binding with BACE1 rather than impaired canonical G-protein activation.

**3. On pseudoreplication in Figure 7C&D:**
We have now reanalyzed the data in Figure 7C&D by:

- Averaging all measurements from multiple sections within the same animal,
- Treating each mouse as one biological replicate (n=4 per group),
- And updating the figure legend and statistics accordingly.

**Response Figure 2.A-B:**

Figure legends: **(A)** Hippocampal tissue of 5xFAD mice injected with Gnb5(28-102aa)-AAV or Gnb5(28-102aa&p81L)-AAV viruses to assess the protein levels of Rgs7, Akt, p-Akt and α-Tubulin served as a loading control; **(B)** Quantitative analysis of the western blot data shown in panel A. Data are presented as mean ± SEM; Statistical significance was determined by two-tailed Student's t-test; *NS:*(P>0.05).

**Revised Figure 7C&D:**

Figure Legends: **(C-D)** Quantification of Aβ plaques size **(C)** or Aβ plaques number **(D)**; Individual data points represent mean values of per mouse derived from 3-5 fluorescence images; Data are presented as mean ± SEM; Statistical significance was determined by one-way ANOVA with Holm-Šidák post hoc tests for multiple comparisons; **P< 0.01; ***P< 0.001; ****P< 0.0001.

> Overall, there are some interesting findings in this study but it is difficult to interpret the significance of these findings or the internal relationship between them, without relating the effects of altering a particular beta subunit to the specific pathways that mediate the changes seen.
***Response:***

We thank the reviewer for this important comment. We agree that clarifying the pathway specificity of the observed effects is essential to fully interpret the functional relevance of Gnb5 in Alzheimer’s disease pathology.

In our current study, we aimed to dissect the role of Gnb5 in the regulation of BACE1 and Aβ pathology. Our findings point to interaction between Gnb5 and BACE1 (Figures 6), and functional rescue experiments using AAV-mediated hippocampal delivery or domain mutants (Figure 7) further support a mechanistic link. These data suggest that at least part of Gnb5’s effect on Aβ production is mediated through modulation of BACE1, rather than indirect or broad G-protein signaling pathways.

We acknowledge, however, that Gnb5 is also a component of multiple GPCR signaling complexes and that altering its levels may influence additional pathways. While our current data prioritize the BACE1 axis as a key downstream effector, we recognize that other Gnb5-associated signaling networks may also contribute to the phenotypes observed. In the revised manuscript, we have expanded the Discussion section (lines 350–365) to highlight this point and discuss other potential signaling mechanisms.

In summary, our study delineates a "Gnb5-BACE1-Aβ" regulatory axis in 5xFAD mice that fundamentally influences cognitive behavioral outcomes. We deeply appreciate your guidance in prompting this clarification and remain fully available to provide any additional information or clarification required.
