# Supplementary material for: Gnb5 is a negative regulator of the BACE1-mediated Aβ generation and ameliorates cognitive deficits in a mouse model of Alzheimer’s disease
Source: PLoS Biol. 2025 Jun 30;23(6):e3003259. doi: 10.1371/journal.pbio.3003259 (PMC12233908; doi:10.1371/journal.pbio.3003259)
Supplement: Attachment — Submitted filename: Response_letter_to_reviewers_auresp_3.docx [file pbio.3003259.s011.docx]

**Response Letter to Reviewer Comments**

The authors would like to thank the efforts of the editorial personnel and the reviewers. The reviewer comments have been numbered and **in black**, with our responses provided **in blue**. The sections of the manuscript that were revised are marked **in red**.

> Dear Dr Zhang,
> Thank you for your patience while we considered your revised manuscript "Gnb5 ameliorates cognitive deficits by reducing β-amyloid deposition through BACE1 in Alzheimer's disease" for publication as a Research Article at PLOS Biology. This revised version of your manuscript has been evaluated by the PLOS Biology editors, the Academic Editor and the original reviewers.
> The reviews are appended below, and you will see that all three reviewers think the paper has been strengthened in the revision. However, reviewer 3 highlights that the study has not conclusively shown that the effects of Gnb5 on cognition are mediated by its modulation of BACE1, and we think this is an important point that should be addressed with textual changes.
> Based on the reviews, we would like to invite you to revise your study, to address reviewer 3's last concerns. We think that it would still be OK for you to lay out the case for why you think the effects of Gnb5 on cognition are likely mediated by changes in BACE1 in the discussion section, but we think that you should also explicitly acknowledge reviewer 3's point in the limitations section, and include a more detailed discussion of potential alternative mechanisms, such as classical G-protein signaling pathways, that might contribute to the cognitive effects observed. We also think that the claims in the title and abstract will need to be toned down. For example, to address reviewer 3's concerns, we think the title could be changed to: "Gnb5 is a negative regulator of the BACE1-mediated Aβ generation and ameliorates cognitive deficits in a mouse model of Alzheimer's disease"
> As you address these items, please take this last chance to review your reference list to ensure that it is complete and correct. If you have cited papers that have been retracted, please include the rationale for doing so in the manuscript text, or remove these references and replace them with relevant current references. Any changes to the reference list should be mentioned in the cover letter that accompanies your revised manuscript.
> In addition to these revisions, you will need to complete some formatting changes, which you will receive in a follow up email. A member of our team will be in touch with a set of requests shortly.
> We expect to receive your revised manuscript within two weeks.
***Response:***

Thank you sincerely for your comprehensive feedback and expert guidance throughout the review process.

**Study limitations clarification:** Regarding Reviewer 3's valuable feedback, we have expanded the Discussion section to clearly point out the limitations of the mechanism (page 17, lines 369-377 in the revised manuscript).

**Title modification:** We are pleased to incorporate the suggested title refinement: "Gnb5 is a negative regulator of the BACE1-mediated Aβ generation and ameliorates cognitive deficits in a mouse model of Alzheimer's disease". Moreover, the abstract has also been revised to tone down the implied direct mechanistic link between Gnb5 and BACE1. Specifically, we now state:

“*Mechanistically, we demonstrated that Gnb5 interacts with BACE1, modulating its expression and* ***potentially*** *influencing Aβ generation.*”

**Reference list review:** We have carefully reviewed the reference list to ensure its accuracy and completeness. We confirm that no retracted papers are cited. Six new citations have been noted in the revised cover letter.

> ------------------------------------------------------------------------
> Reviewer remarks:
> Comments from the Academic Editor (who was originally Reviewer #1): Most of my concerns have been addressed adequately. 
***Response:***
We sincerely thank the Academic Editor for their careful review and constructive feedback throughout the revision process. We are pleased to hear that most of the concerns have been adequately addressed. We remain committed to ensuring the clarity, rigor, and completeness of our work, and we greatly appreciate your guidance in improving the manuscript.

> Reviewer #2: The authors have adequately addressed the concerns.
***Response:***
We would like to extend our sincere gratitude for the time and expertise you have invested in evaluating our work. Your rigorous assessment and constructive feedback during the revision process have significantly enhanced both the scientific rigor and clarity of our manuscript.

> Reviewer #3: In most cases the changes made and responses from the authors address my concerns well. The one point that I think still needs clarification is the assumption that the behavioural effects seen are related to the BACE1 modulation or indeed to Alzheimer's disease. There is a logical disconnect in the authors response.
> 1. It is true that they have demonstrated that the overexpression of GnB5 reduces amyloid beta production through BACE1 and that this is probably through an non canonical pathway rather than classic g-protein modulation. Moreover overexpression of GnB5 decreases amyloid load.
> 2. They have also demonstrated that there is a decrease in GnB5 in the 5xFAD mice and that knocking out GnB5 causes behavioural deficits while overexpression of GnB5 in 5xFAD mice rescues the behavioural deficits.
> However they have not demonstrated that the behavioural changes are due to the BACE1 effects, rather than due to some other classical G-protein mediated pathway. The behavioural changes seem to be dependent on GnB5 levels but this would affect many pathways and so it cannot be assumed that just because BACE1 is one of the pathways affected that the behavioural changes are due to modulation of BACE1.
> This needs to be clarified in the discussion and the title should be changed accordingly
***Response:***

We thank Reviewer #3 for their thoughtful and constructive feedback. We appreciate the recognition that our revisions have addressed most of the concerns raised in the initial review. We also acknowledge and fully agree with the reviewer’s point that, although our data support a role for Gβ5 (Gnb5) in modulating BACE1 and amyloid-beta production, the causal link between these molecular effects and the observed behavioral outcomes remains indirect and could involve additional pathways.

As correctly noted, Gnb5 is involved in multiple signaling cascades, including classical G-protein-mediated pathways. Therefore, while our results demonstrate that Gβ5 overexpression rescues cognitive deficits in 5xFAD mice, we cannot definitively conclude that these effects are mediated solely via modulation of BACE1 activity. We agree this is an important distinction that should be made clearer.

To address this, we revised the Discussion section to explicitly note the possibility that behavioral rescue may involve BACE1-independent mechanisms, potentially including classical G-protein signaling or other non-canonical interactions of Gβ5. We will also clarify that while our data support an association between Gβ5 and reduced amyloid pathology, the behavioral outcomes are more broadly influenced by Gβ5-dependent signaling and are not necessarily attributable to BACE1 modulation alone.

The revised section of the Discussion now reads as follows:

“*Moreover, while our data demonstrate that Gnb5 interacts with BACE1 and is associated with reduced Aβ production, the cognitive improvements observed in 5xFAD mice are unlikely to be solely attributed to BACE1 modulation. G protein activation, including that mediated by Gnb5, modulates multiple intracellular signaling pathways that involve second messengers such as cAMP and calcium, as well as downstream kinases like PKA and PKC [54, 55]. These pathways may independently contribute to the cognitive improvements observed [56-59], suggesting that Gnb5 may influence AD-related phenotypes through both BACE1-dependent and BACE1-independent mechanisms.*” (page 17, lines 369-377)

Furthermore, in response to the suggestion regarding the manuscript title, we agree that the current title may imply a more direct mechanistic link than our data can support.

We revised the title as “Gnb5 is a negative regulator of the BACE1-mediated Aβ generation and ameliorates cognitive deficits in a mouse model of Alzheimer's disease” to reflect the broader scope of the findings and avoid overstating the role of BACE1.

Six additional references:

*54.	Zhang M, Chen T, Lu X, Lan X, Chen Z, Lu S. G protein-coupled receptors (GPCRs): advances in structures, mechanisms, and drug discovery. Signal Transduct Target Ther. 2024;9(1):88.*

*55.	Liu S, Anderson PJ, Rajagopal S, Lefkowitz RJ, Rockman HA. G Protein-Coupled Receptors: A Century of Research and Discovery. Circ Res. 2024;135(1):174-97.*

*56.	Ning Z, Zhong X, Wu Y, Wang Y, Hu D, Wang K, et al. beta-asarone improves cognitive impairment and alleviates autophagy in mice with vascular dementia via the cAMP/PKA/CREB pathway. Phytomedicine. 2024;123:155215.*

*57.	Abjorsbraten KS, Skaaraas G, Cunen C, Bjornstad DM, Binder KMG, Bojarskaite L, et al. Impaired astrocytic Ca(2+) signaling in awake-behaving Alzheimer's disease transgenic mice. Elife. 2022;11.*

*58.	Pereda D, Al-Osta I, Okorocha AE, Easton A, Hartell NA. Changes in presynaptic calcium signalling accompany age-related deficits in hippocampal LTP and cognitive impairment. Aging Cell. 2019;18(5):e13008.*

*59.	Alkon DL, Sun MK, Nelson TJ. PKC signaling deficits: a mechanistic hypothesis for the origins of Alzheimer's disease. Trends Pharmacol Sci. 2007;28(2):51-60.*
